# Deciphering the evolution of Deception Island’s magmatic system

**DOI:** 10.1038/s41598-018-36188-4

**Published:** 2019-01-23

**Authors:** A. Geyer, A. M. Álvarez-Valero, G. Gisbert, M. Aulinas, D. Hernández-Barreña, A. Lobo, J. Marti

**Affiliations:** 1 Institute of Earth Sciences Jaume Almera, ICTJA, CSIC, Lluis Sole i Sabaris s/n, 08028 Barcelona, Spain; 2 Departamento de Geología, Universidad de Salamanca, 37008 Salamanca, Spain; 3 Instituto de Geociencias, CSIC-UCM, Severo Ochoa 7, 28040 Madrid, Spain; 4 Departament de Mineralogia, Petrologia i Geologia Aplicada, University of Barcelona, Marti Franques s/n, 08028 Barcelona, Spain

## Abstract

Deception Island (South Shetland Islands) is one of the most active volcanoes in Antarctica, with more than 20 explosive eruptive events registered over the past two centuries. Recent eruptions (1967, 1969, and 1970) and the volcanic unrest episodes that happened in 1992, 1999, and 2014–2015 demonstrate that the occurrence of future volcanic activity is a valid and pressing concern for scientists, technical and logistic personnel, and tourists, that are visiting or working on or near the island. We present a unifying evolutionary model of the magmatic system beneath Deception Island by integrating new petrologic and geochemical results with an exhaustive database of previous studies in the region. Our results reveal the existence of a complex plumbing system composed of several shallow magma chambers (≤10 km depth) fed by magmas raised directly from the mantle, or from a magma accumulation zone located at the crust-mantle boundary (15–20 km depth). Understanding the current state of the island’s magmatic system, and its potential evolution in the future, is fundamental to increase the effectiveness of interpreting monitoring data during volcanic unrest periods and hence, for future eruption forecasting.

## Introduction

**Figure 1:**
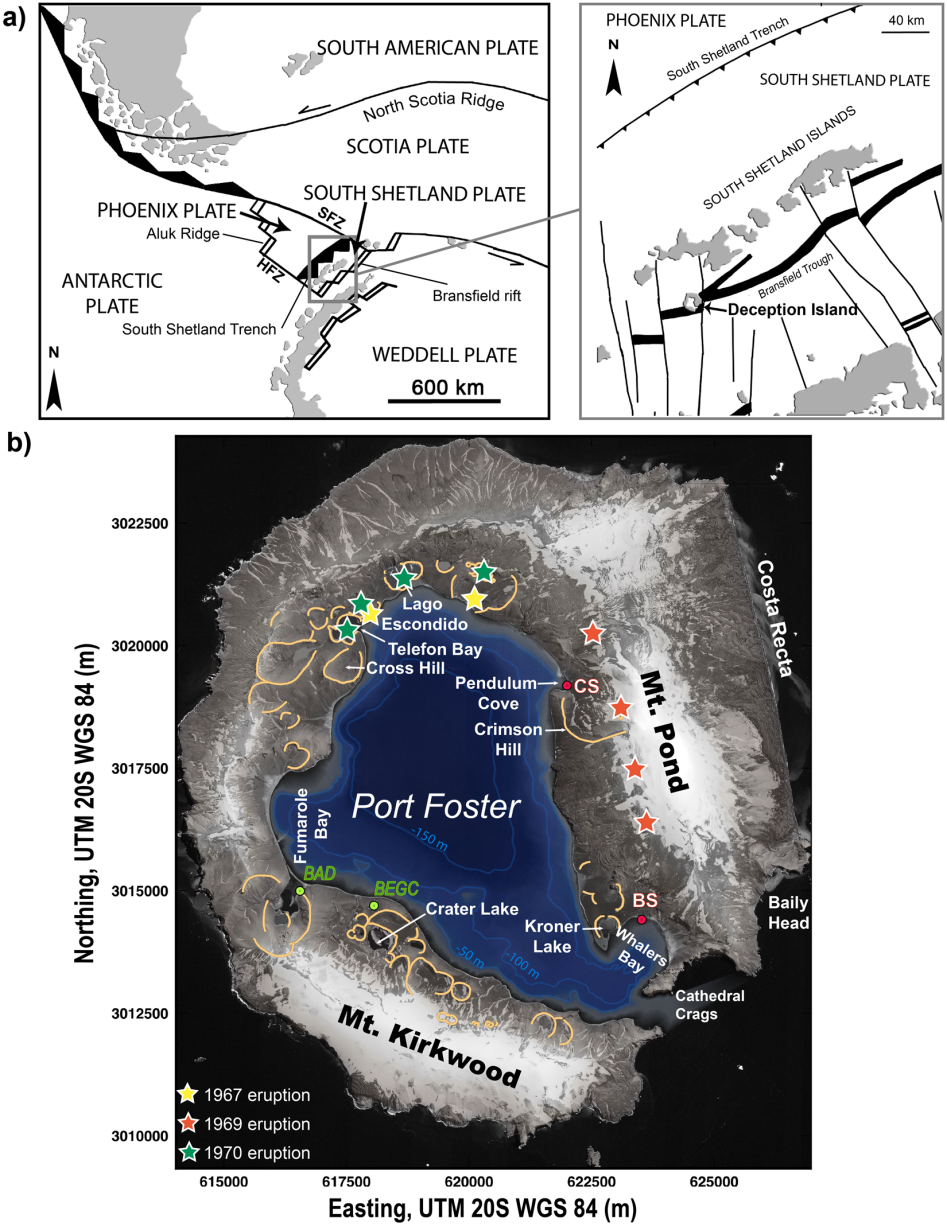
(**a**) Simplified regional tectonic map and location of the South Shetland Islands (modified from Marti *et al*.^36^). HFZ Hero Fracture Zone, SFZ Shetland Fracture Zone. (**b**) Deception Island orthophotomap (data obtained from Spatial Data Infrastructure for Deception Island SIMAC, Torrecillas *et al*.^81^) where active and destroyed scientific stations, post-caldera volcanic craters (orange lines) and the sites of the most recent volcanic eruptions (colored stars) are indicated. BAD Argentinian Base “Decepción”; BEGC Spanish Base “Gabriel de Castilla”; BS British Base (destroyed); CS Chilean Base (destroyed). This figure was generated with QGIS software version 2.18 Las Palmas (available at: www.qgis.org). Final layout was obtained with Adobe Illustrator CC 2015.3.1 (Copyright © 1987–2016 Adobe Systems Incorporated and its licensors).

Deception Island (DI), discovered in 1820, is amongst the most active volcanoes in Antarctica with a record of over 20 explosive eruptions in the last two centuries^1–3^. Located in the South Shetland Islands at the spreading centre of the Bransfield Strait marginal basin (Fig. 1a), the island currently hosts two scientific stations operating yearly during the austral summer season and is one of the most popular touristic destinations in Antarctica with over 15,000 visitors per year (IAATO, *International Association of Antarctica Tour Operators*, *2018*).

Deception Island’s historic volcanic events, generally small in volume (e.g., < 0.1 km^3^), have been concentrated in periods of great activity (e.g., 1906–1912, 1818–1828), followed by decades of dormancy (e.g., 1912–1967)^3^. However, the usual presence of DI tephras in distal (> 500 km distance) marine sediments^4^ and ice cores^5,6^ hints that several past eruptions may have been significantly larger and more violent (VEI > 4–5). The recorded historical volcanic activity, the recently experienced eruptions (1967, 1969, and 1970) and the unrest episodes happened in 1992, 1999^7^, and 2014–2015^8^ categorise DI as a very active volcano. Therefore, the occurrence of future volcanic activity would become a serious cause for concern for scientists (see for instance the destruction of the recent Chilean and British scientific bases), technical and logistic personnel, and tourists, staying on the island or nearby.

To a much greater extent, results of numerical simulations using meteorological and atmospheric transport models estimate that volcanic ash emitted by even a moderate eruption occurring today in DI could potentially encircle the southern hemisphere, leading to significant economic losses and consequences for global aviation safety^9^. Indeed, results obtained indicate that the volcanic ash clouds could reach up to tropical latitudes, such as the Atlantic coast of South America, South Africa and/or Oceania. In general, the highest ash concentrations in the atmosphere (> 100 g /m^2^) would be mainly found over the Atlantic Ocean, the Scotia and the Weddell seas during the first 48 h after the eruption start^9^. However, a residual small amount of ash (0.1–1 g /m^2^) may potentially remain in the atmosphere up to over a week after the eruption onset. Ash concentrations above the flight safety thresholds (0.2–2 mg /m^3^) may be observed over South Africa and, in some cases, also over southern Australia or even over austral Patagonia, affecting international and domestic flying routes, in addition to flights connecting Africa with South America and Australia^9^.

Important efforts have been made to understand the magmatic and volcanic evolution of DI, the nature of the underlying magmatic sources, and their relation to the geodynamic setting (e.g.,^3,10–27^). However, a detailed evolutionary model of the island’s magma plumbing system has never been provided. As a consequence, even if an eruption on DI is certain to occur in the near future, the timescale and characteristics of that volcanic activity still remain unclear^3^. During volcanic unrest periods, this lack of knowledge considerably diminishes the effectiveness of interpreting recorded monitoring data. This reduces the capacity of envisaging the potential outcome scenarios, which may also include new eruptions.

In this paper, we propose a new and all-encompassing evolutionary model of DI’s magmatic system following an interdisciplinary approach that combines petrological and geochemical data (Supplementary Materials 1–6) with geophysical observations (Supplementary Material 7), detailed Pressure-Temperature (P-T) estimates, and fractional crystallization modelling (Supplementary Material 8). For this purpose, we have created a comprehensive geochemical database of DI’s rock samples including new analytical results (Supplementary Material 1) and an exhaustive review of published data (e.g.,^3,13–15,27–30^) (Supplementary Materials 2 and 3). Finally, we assessed the major element concentrations through Linear Discriminant Analysis (LDA)^31,32^ to gain extra support for the proposed model (Supplementary Material 8). The conclusions are crucial to comprehending the past, present, and future states of the magmatic system of DI, as well as post-caldera activity of other restless volcanic caldera systems with similar characteristics. This will significantly improve the capacity for decoding monitoring data recorded during a volcanic crisis and hence, will serve to inform future eruption forecasts.

## Deception Island: Geological overview

DI is a composite volcano with a basal diameter of 30 km and rising 1,400 m from the seafloor to a maximum height of 540 m above sea level^33^. The emerged part of the volcano leads to a horseshoe-shaped 15-km-diameter island, whose central part is occupied by a sea-flooded volcanic collapse caldera (Port Foster) with dimensions of about 6 × 10 km, and a maximum water depth of 190 m (Fig. 1). The normal magnetic polarity of all DI’s exposed rocks indicates that these are younger than 0.75 Ma^34^, and K-Ar data^35^ suggest that most of the subaerial part of the island was built in the last 0.2 Ma. The correlation between DI’s *in situ* deposits and its tephra layers found elsewhere in the region implies that exposed rocks appear to be younger than 100 ka^3,36^.

DI is located near the intersection between the extension of the Hero Fracture Zone and the south-western end of the Bransfield Strait (BS). The latter consists of a NE–SW oriented, 500-km-long and 100-km-wide, extensional basin that separates the South Shetland continental microplate from the Bransfield Platform^37–39^ (Fig. 1a). The formation of the Bransfield Rift, a Late Cenozoic extensional structure (15–20 km wide)^40^, has been interpreted to be the consequence of back-arc extension linked to subduction of the Phoenix Plate beneath the Antarctic Plate^41^. Today, slab subduction is still on-going at the South Shetland trench, as indicated by seismicity^42^, but at very low velocities (estimated convergence rates range between 2.5 and 7.5 mm/a for the last 2 Ma^43^*)*. This complex regional geodynamics, a combination of subduction and back-arc spreading processes, has conditioned timing and composition of magmatism in the region^29,44,45^ (Supplementary Material 6). Quaternary magmatism, strongly connected to rifting and back-arc basin formation, is mostly concentrated at Deception, Penguin, and Bridgeman Islands^45–47^.

**Figure 2:**
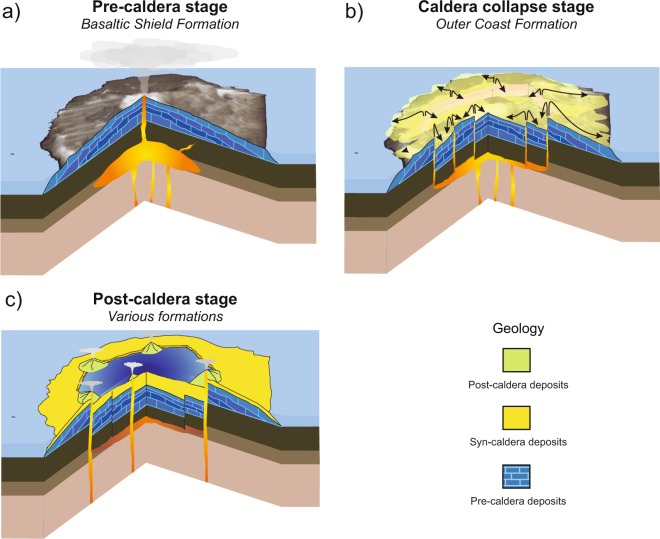
Simplified sketch illustrating the different stages of Deception Island’s evolution (modified from Martí *et al*.^36^). This figure was generated with QGIS software version 2.18 Las Palmas (available at: www.qgis.org). Final layout was obtained with Adobe Illustrator CC 2015.3.1 (Copyright © 1987–2016 Adobe Systems Incorporated and its licensors).

**Figure 3:**
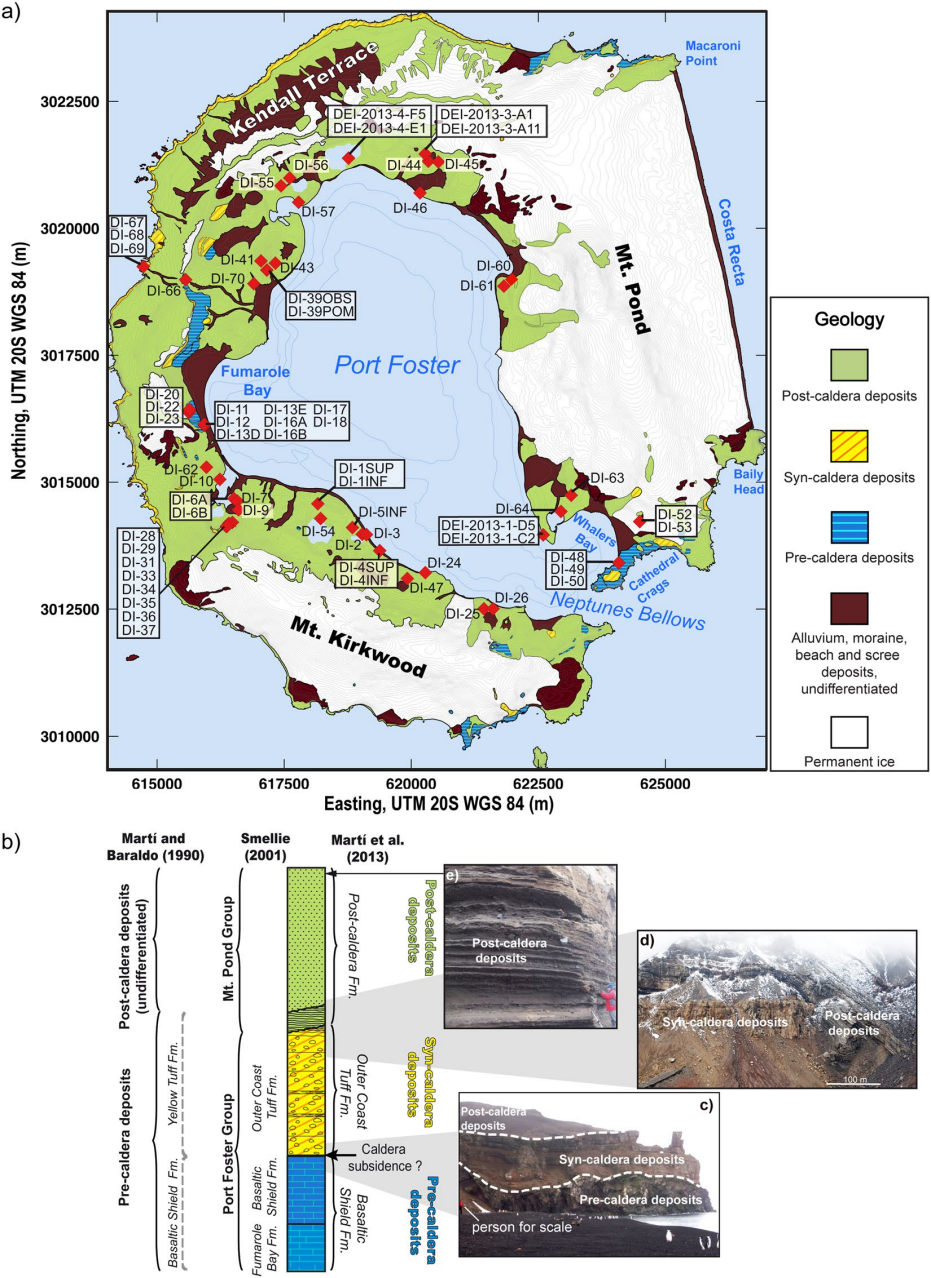
(**a**) Simplified geological map of Deception Island (modified from Martí *et al*.^36^) and location of the analysed samples. Synthetic stratigraphic section of Deception Island indicating the divisions proposed in previous studies at the sides of the stratigraphic log (on the left). Photographic view of (**c**) Cathedral Crags (looking SE), (**d**) Vapour Cole (looking S) and (**e**) inland craters of the 1970 eruption (looking NW). Data obtained from Spatial Data Infrastructure for Deception Island SIMAC, Torrecillas *et al*.^81^. This figure was generated with QGIS software version 2.18 Las Palmas (available at: www.qgis.org). Final layout was obtained with Adobe Illustrator CC 2015.3.1 (Copyright © 1987–2016 Adobe Systems Incorporated and its licensors).

DI’s volcanic evolution is marked by a caldera collapse, which took place between 8,300 and ∼3,980 years BC^48,49^. The pre-caldera evolutionary stage was characterized by the formation of multiple coalesced shoaling seamounts and a subaerial volcanic shield^3^ (Figs. 2a and 3). The main syn-caldera depositional unit, known as the Outer Coast Tuff Formation (OCTF), mainly corresponds to pyroclastic density current deposits (including mostly basaltic-andesitic ignimbrites and surges) that are several tens of meters thick (Fig. 3c). The morphological features of DI (e.g., the existence of a depression in the centre of the island, the apparent circular shape of the caldera rim, the location of post-caldera vents along the edge of the depression, etc.) support a piston-like collapse model, either along ring faults or a series of regionally induced intersecting faults, following a major eruption^36^ (Fig. 2b). Indeed, it has been estimated that over 60 km^3^ of magma erupted during the caldera event, classifying DI as a medium-sized caldera with similar dimensions as Krakatoa or Santorini^50^.

The post-caldera phase, which includes the recent historical eruptions (1829–1970), comprises at least 70 scattered eruptive vents inside the caldera, except one located along the structural borders of the caldera itself ^3,36^ (Figs. 1 and 2c). Recent post-caldera volcanic activity on DI mostly consists of small volume eruptions (e.g., <0.1 km^3^)^3,24,36^ with variable degrees of explosivity depending on the water amount and source (i.e., aquifer, sea, ice melting, etc.) that interacted with the rising or erupting magma^3,15,51,52^.

## Deception Island’s magmatic system: A unifying evolutionary model

Numerous studies have been carried out seeking full comprehension of DI’s magmatic picture (e.g.,^22,24,28,53–55^). We present the first interdisciplinary approach to outline a unifying evolutionary model of the island’s magma plumbing system by combining petrological, geochemical, and geophysical data.

**Figure 4:**
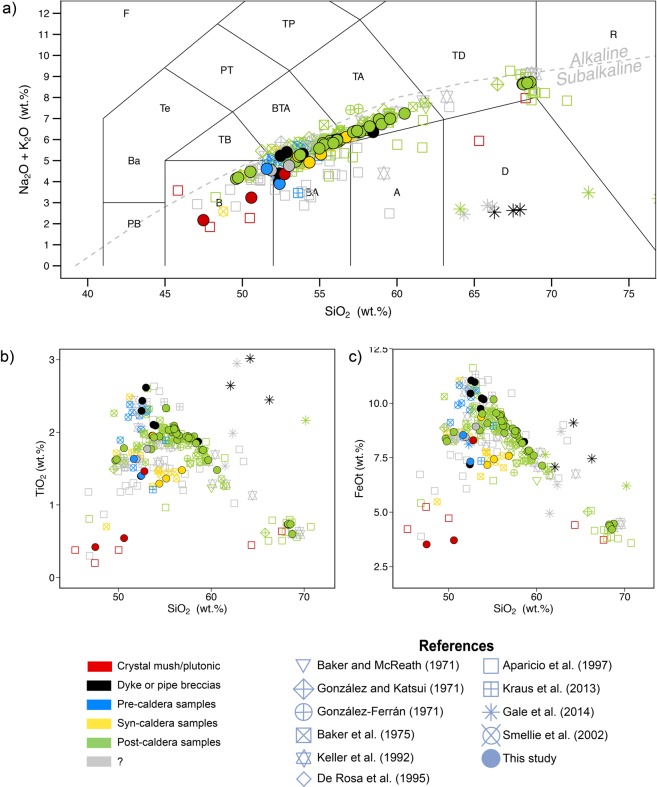
(**a**) Total Alkali vs. Silica diagram (TAS)^82^ for the rock samples considered in this work (see Supplementary Materials 1–2 for details on composition and exact latitude-longitude coordinates of the rock samples). Major elements normalized to 100% (anhydrous) with Fe distributed from FeO to Fe_2_O_3_ following Middlemost^83^. Grey dashed line discriminates between the alkaline and subalkaline fields^84^. TiO_2_ (**b**) and FeOt (**c**) vs. SiO_2_ content Harker Diagrams for the rock samples considered in this work. See Supplementary Materials 1–2 for details on composition and exact latitude-longitude coordinates of the rock samples). This figure was generated with RStudio Version 1.0.143 (https://www.rstudio.com/) using ggplot2 package Version 2.1.9000 (http://www.ggplot2.org)^85^, a plotting system for R. Final layout of this figure was achieved using Adobe Illustrator CC 2015.3.1 (Copyright © 1987–2016 Adobe Systems Incorporated and its licensors).

Geochemical data show that DI’s magmas range from basaltic to trachydacitic and rhyolitic compositions defining a distinctive alkalinity-increasing differentiation trend produced by unusually high Na_2_O contents (between 2–8 wt.% Na_2_O) (Fig. 4a, Supplementary Materials 5 and 6). Compositionally, this feature forces DI magmas to deviate from the normal active arc andesite-rhyolite associations in the circum-Pacific areas^10^, rather having Na/K ratios similar to mid-oceanic ridge basalts^3^ (Supplementary Material 6). As revealed by regional geochemical data, DI’s magma signature indicates a mantle source similar to the one feeding the Bransfield Rift areas of subalkaline composition and with little subduction influence (i.e., depleted N-MORB mantle with minor subduction component contribution; Supplementary Material 6). Its higher alkalinity and incompatible trace element enrichment (i.e., higher Nb/Zr ratios) compared to those of the Bransfield Rift may suggest a lower partial melting contribution (Supplementary Material 6). This in turn is consistent with the marginal location of DI relative to both the rift (lower extension/decompression in DI) and the subduction-dominated arc (lower water content in the mantle source of DI magmas).

Pre-caldera magmas are among the less evolved on DI, with compositions ranging from basaltic to basaltic-andesitic and basaltic-trachyandesitic (Fig. 4a, Supplementary Material 5). Pressure estimates on the pre-caldera samples (e.g., DI-12, DI-50) indicate that, during this stage, some of these magmas ascended directly from pressures >6.5 kbar (i.e., depths ≥ 25 km, assuming an average crust density of 2650 kg/m^3^), which suggests a mantle origin (Moho depth beneath DI is between 15 and 20 km deep^56^). The estimated stagnation depths of further evolved pre-caldera magmas (between 15 and 20 km depth, P ∼ 4–5 kbar) reveal the accumulation of magmatic material at the crust-mantle boundary (R1, Figs. S8–2) (e.g., DI-23, DI-48), similar to other volcanic areas^57–60^.

**Figure 5:**
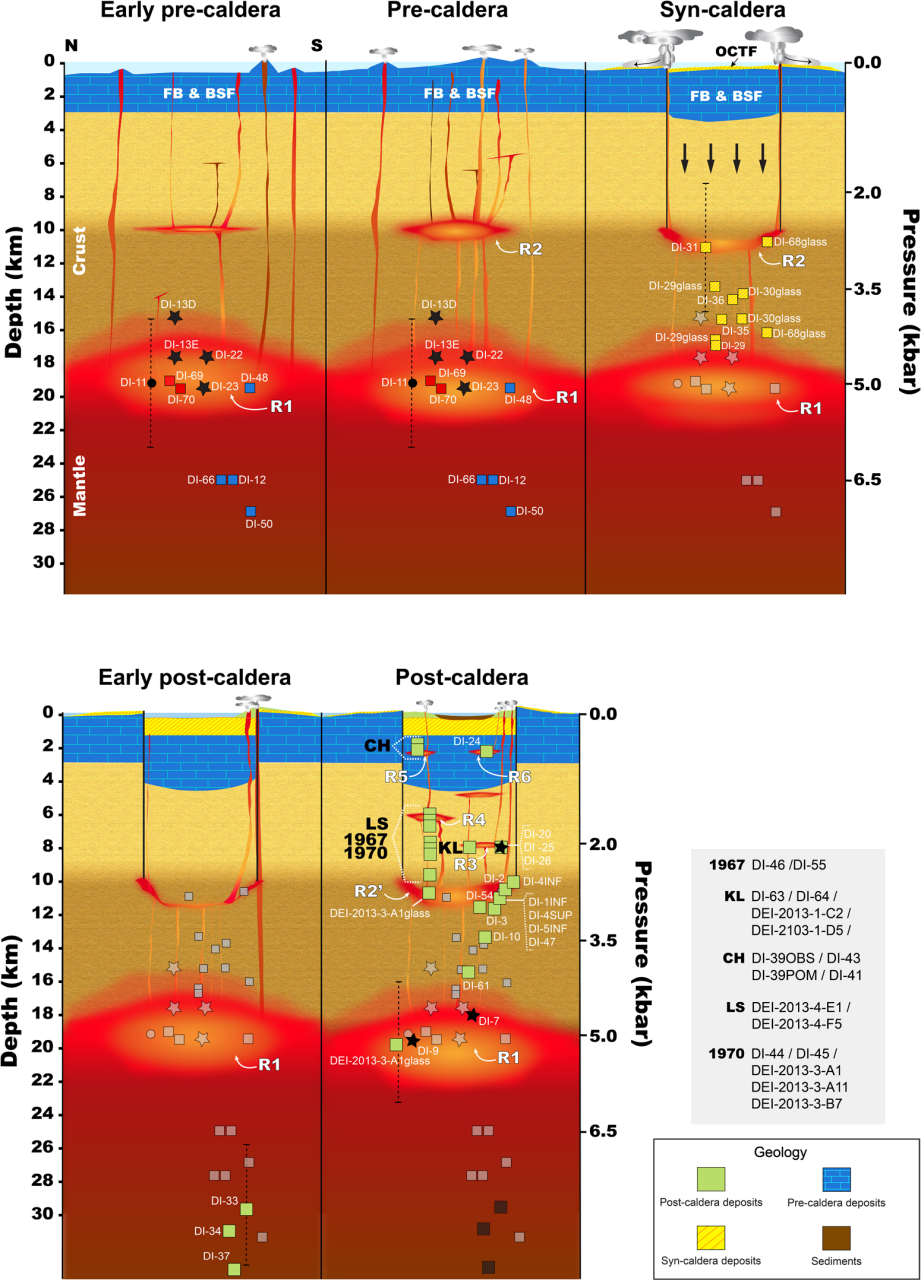
Conceptual model of the magmatic system of Deception Island based on P-T estimates. See text for more details. CH Cross Hill eruption; KL Kroner Lake eruption; LS Lago Escondido eruption.  This figure was generated with Adobe Illustrator CC 2015.3.1 (Copyright © 1987–2016 Adobe Systems Incorporated and its licensors).

Magmas erupted during the caldera-forming event (i.e., syn-caldera magmas, OCTF samples) group into (Figs. 4b,c, Supplementary Material 5 and 8): (i) a main compositional cluster that comprises most of the samples that deviate from the principal chemical DI trends and that corresponds to the “second magma series” proposed by Smellie *et al*.^3^, and ii) within the main DI geochemical trends at < 55 wt.% SiO_2_. Pressure estimates of OCTF samples reveal a syn-caldera magma provenance depth from 11 to 19 km (Fig. 5). This depth range correlates to the main cluster samples, whose mineral assemblage either (i) equilibrated at ∼3 kbar (e.g., DI-31, DI-68), or; ii) incompletely equilibrated—because of a later arrival—at ∼3.5–5 kbar (e.g., DI-35, DI-36) prior to eruption.

Considering this, we assert that rocks included in the main compositional cluster correspond to magmas stagnated in a shallow magma reservoir (R2, ∼10–11 km depth, P ∼ 3 kbar), which was directly responsible for the caldera-forming event. The accumulation depth of this shallower R2 reservoir was most likely promoted by the contact between the upper and middle crust located within the same depth range (Fig. 5, Supplementary Materials 7 and 8). The loading stress related to the growing basaltic shield structure may have also favored the ascending basaltic magmas to stop at shallower depths (e.g.,^61^). Comparable magma stagnation pressures have been estimated for other well-known volcanic calderas (e.g., Aira, Japan^62^).

The more basic OCTF samples falling outside the main cluster (though still along the main DI differentiation trend), would correspond to magmas coming from deeper sources (i.e., R1). The arrival of these hotter and more primitive magmas into reservoir R2 may have triggered the explosive eruption leading to the caldera formation^63,64^ as already suggested by Smellie *et al*.^3^. In line with other examples of caldera-forming events, all eruptible material would have been extruded from the magma chamber during collapse, fully or partially destroying R2^65–68^. Note that under the term “eruptible magma”, we understand that magma capable of being withdrawn during an eruptive event including^69–71^: (i) crystal-poor magma (< 15% crystals), (ii) crystal-rich magma (15–45% crystals); and (iii) crystal mush (barely eruptible, up to 50–60% crystals).

Magmas that erupted after the caldera collapse outline a well-defined evolutionary trend, showing the widest compositional range on DI, from basalts to rhyolites. Overall, major and trace element compositions of post-caldera magmas define a tholeiitic trend with initial TiO_2_ and FeOt enrichment related to delayed Fe-Ti oxide crystallization and fractionation^3,15^.

P-T estimates of the first magmas erupted after the caldera-forming event (P > 7.5 kbar, > 28.5 km), which are among the most primitive analysed in this suite (Supplementary Materials 5 and 8), suggest a direct ascent from the mantle magma source (Fig. 5). The time span between the deposition of the syn-caldera deposits and the eruption of these magmas is still uncertain, hindering the full understanding of their genesis. These magmas could be: (i) coeval to the end of the caldera-forming event, representing either the most primitive of the deeper basic magmas that triggered the caldera-forming eruption, or unrelated magmas whose ascent and eruption was favoured by the opening and depressurization of the plumbing system during the caldera-forming event; (ii) magmas emitted after a significant time, which would imply a direct magma ascent without stagnation at intermediate depths through the formation of new ascent paths outside the reservoir areas, or that ascend through inactive reservoirs (e.g., reservoirs that collapsed during caldera formation or were significantly solidified).

Magma compositions and P-T estimates of juvenile samples from the late post-caldera stage, including historical eruptions, hint that erupted magma can be either directly supplied by the magma accumulation zone at the crust-mantle boundary R1 or by diverse magma batches located at distinct shallow (< 10 km) depths (R3–R6) (Fig. 5). Pressure estimates from recent post-caldera juvenile samples from Crater Lake area (e.g., DI-1INF, DI-4SUP) (Fig. 3), point to the existence of a magma source located at similar depths, such as the presumably destroyed reservoir R2. This may indicate that, since the collapse event, new pulses of fresh magma coming from R1 or directly from the mantle, would have created new chambers at comparable depths (R2′).

The compositional variability of the 1967 and 1970 eruptive products, which depict approximately linear trends in binary diagrams, have been interpreted as related to mingling and mixing processes, as well as stratification of the magma reservoir^15^ (named R4, Supplementary Materials 5 and 8). In contrast to the wide compositional range of the 1967 and 1970 eruptions feeding reservoir, some eruptive cones emitted compositionally very restricted magmas^3,52^, indicating that more homogeneous magma reservoirs are also present in the system (e.g., Kroner Lake eruption, reservoir R3)(Fig. 5). Additionally, glass compositions of 1970 samples (e.g., DEI-2013-3-A1) are coherent with stagnation depths equivalent to the R1 and R2′ reservoirs (Fig. 5), suggesting that the latter would have fed shallower (P < 2 kbar), and likely smaller chambers responsible for the supply of the several recent eruptions across the island (Supplementary Materials 5 and 8). In this sense, magma stagnation in shallower reservoirs (P < 1 kbar) within a cooler country rock (e.g., R5 or R6) promotes faster and larger differentiation, thus generating the most evolved magma compositions in the DI system (e.g., Cross Hill eruption samples DI-39OBS, DI-41) (Fig. 5). This might be similar to the caldera-collapse event, in which the arrival of fresh and hotter magma from the deeper reservoirs could have acted as an eruption trigger for the case of the recent eruptions (see glass mixing evidence in compositional figures of Supplementary Materials 5 and 8).

Thermodynamical modelling with rhyolite-MELTS software v.1.2.0^72–74^, performed to test the consistency of trends observed in the DI suite with magma differentiation through fractional crystallization processes, has also provided information on the most likely H_2_O content, P, and fO_2_ conditions under which magmas evolved. Our thermodynamic estimates suggest that small differences in H_2_O content and/or fO_2_ conditions during the parental magma evolution are enough to account for the compositional trend difference between pre- and post-caldera magmas since no large major element compositional change in the parental magma is required. Our results indicate that DI magmas form through fractional crystallization of basaltic melts with an initial 0.5–0.75 wt.% H_2_O under fO_2_ conditions of 0–1 log units above Quartz-Fayalite-Magnetite (QFM) buffer at pressures from 2 to 5 kbar, which are in accordance with the proposed model (Supplementary Material 8). The modelled fractionating mineral assemblage consists of: (i) Cpx, Pl, and Spl for the first part of the differentiation trend (from 52.3 to 55.8 wt.% SiO_2_), and (ii) Cpx, Pl, rhombohedral oxides, Spl, and late Apt and Ol (Fa) for the second part of the differentiation trend (55.8–66.6 wt.% SiO_2_).

**Figure 6:**
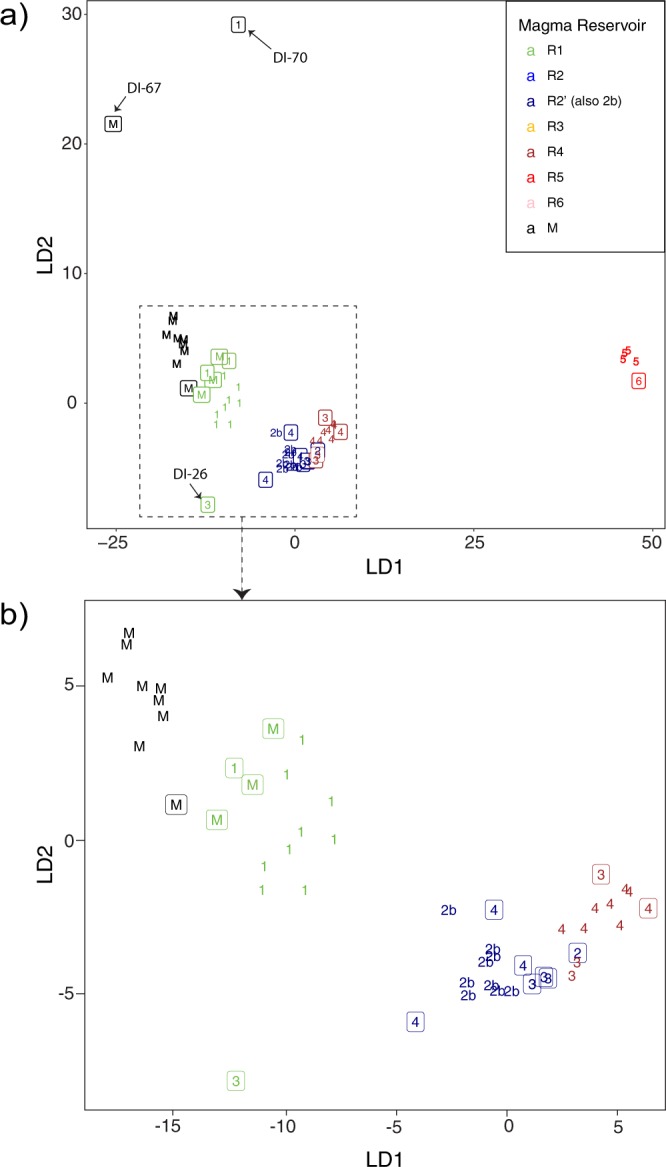
Ordination of samples on the plane defined by the first two LD components. Labels correspond to magma reservoirs according to the proposed model. Colours correspond to the LDA classification. Samples within squares are those not having been included in the calculation of LD components but projected on them and attributed to their closest class. Note that reservoir R2′ has been labelled as “2b”in the graph for visual purposes. This figure was generated with RStudio Version 1.0.143 (https://www.rstudio.com/) using ggplot2 package Version 2.1.9000 (http://www.ggplot2.org)^85^, a plotting system for R. Final layout of this figure was achieved using Adobe Illustrator CC 2015.3.1 (Copyright © 1987–2016 Adobe Systems Incorporated and its licensors).

Finally, Linear Discriminant Analysis (LDA)^31,32^ results present evidence for the general consistency of the proposed model of magma reservoirs in terms of major element geochemistry (Fig. 6). All suggested reservoirs with the exception of R3 have distinct major element compositions and are ordinated from Mantle to R6 along the major LD axis. R5 samples have the most distinct composition, with sample DI-24 (R6) being closer, yet clearly unclustered. This provides solid evidence for the actual existence of the proposed R6 reservoir.

The compositional similarity (in major element geochemistry) between R3 (i.e., the Kroner Lake eruption) and R4 (i.e., the 1967 and 1970 eruptions) is highlighted by the LDA results. Samples initially assumed to belong to R3 are actually classified as either R2′ (presumably, the reservoir that feeds R3 and R4) or R4, and most of them are intermediate between the core of the R4 and R2′ groups. The wide compositional ranges of the 1967 and 1970 eruptive materials (all assigned to R4), which are interpreted as related to magma mingling and mixing, and to reservoir stratification^15^ (Supplementary Material 5), when compared to the compositional homogeneity of the R3 samples, allows assuming the existence of at least two distinct reservoirs at similar depths.

## Discussion: Implications for volcano monitoring and volcanic hazard assessment

Our new evolutionary model for DI’s plumbing system is key to improve—and correlate—the interpretation of current geophysical data, such as monitoring signals recorded during volcanic crises. At DI, the geophysical anomalies of physical properties observed between 2 and up to 6–10 km depth (e.g., low resistivity values^75^, strong seismic velocity variations^53,54^, a very low density anomaly in both magnetic and gravity anomaly maps^76^) have been traditionally interpreted as evidence for the presence of partially melted rock/material beneath the island (e.g.,^53–55,75,76^) (Supplementary Material 7). Our results strengthen this idea and corroborate that magmas feeding DI post-caldera eruptions, including historic events, are raised mainly from an ~2–10 km depth range. We also confirm that erupted magmas did not belong to a single magma batch as had been previously suggested (e.g.,^53,54^), but instead to a complex network of individual, potentially interconnected, shallow reservoirs of variable size, volume and composition (R2′–R6). Magmas feeding the shallowest part of DI’s plumbing system (Fig. 5) would ascend directly from the mantle or the magma stored at the crust-mantle boundary (i.e., 15–20 km depth; R1). The described geochemical data and P-T estimates indicating the existence of melted material accumulated at the Moho discontinuity is in agreement with the low P-wave velocities registered in the upper mantle beneath DI, which are already interpreted as being due to the presence of partially melted material at depth^56^.

At DI, the lack of information regarding residence times of post-caldera magmas hinders a proper assessment of the time elapsed between the reservoir’s formation and its complete cooling, i.e., crystallization. As a consequence, estimating the average lifetime of the individual shallow chambers (R2′–R6) as a source of eruptible magma remains an important challenge. This is crucial to accurately understand and evaluate: (i) what geophysical methods today can image beneath the island, and (ii) the volume of eruptible magma under DI. Accordingly, the island’s eruptive potential in the near future is difficult to assess. For this purpose, and to seek a first order approximation of the potential average lifetime of the individual reservoirs that feed post-caldera eruptions, we have conducted magma chamber cooling models, which solve the heat transfer equation using the Finite Element (FE) method (Supplementary Material 9). For the sake of simplicity, we only consider heat transfer by conduction and discard episodes of magma chamber replenishment^77,78^. Since internal convection and injections of fresh and hotter magmas tend to delay any magma cooling process, the present numerical simulations provide a minimum estimate of the required crystallization times^77,78^.

**Figure 7:**
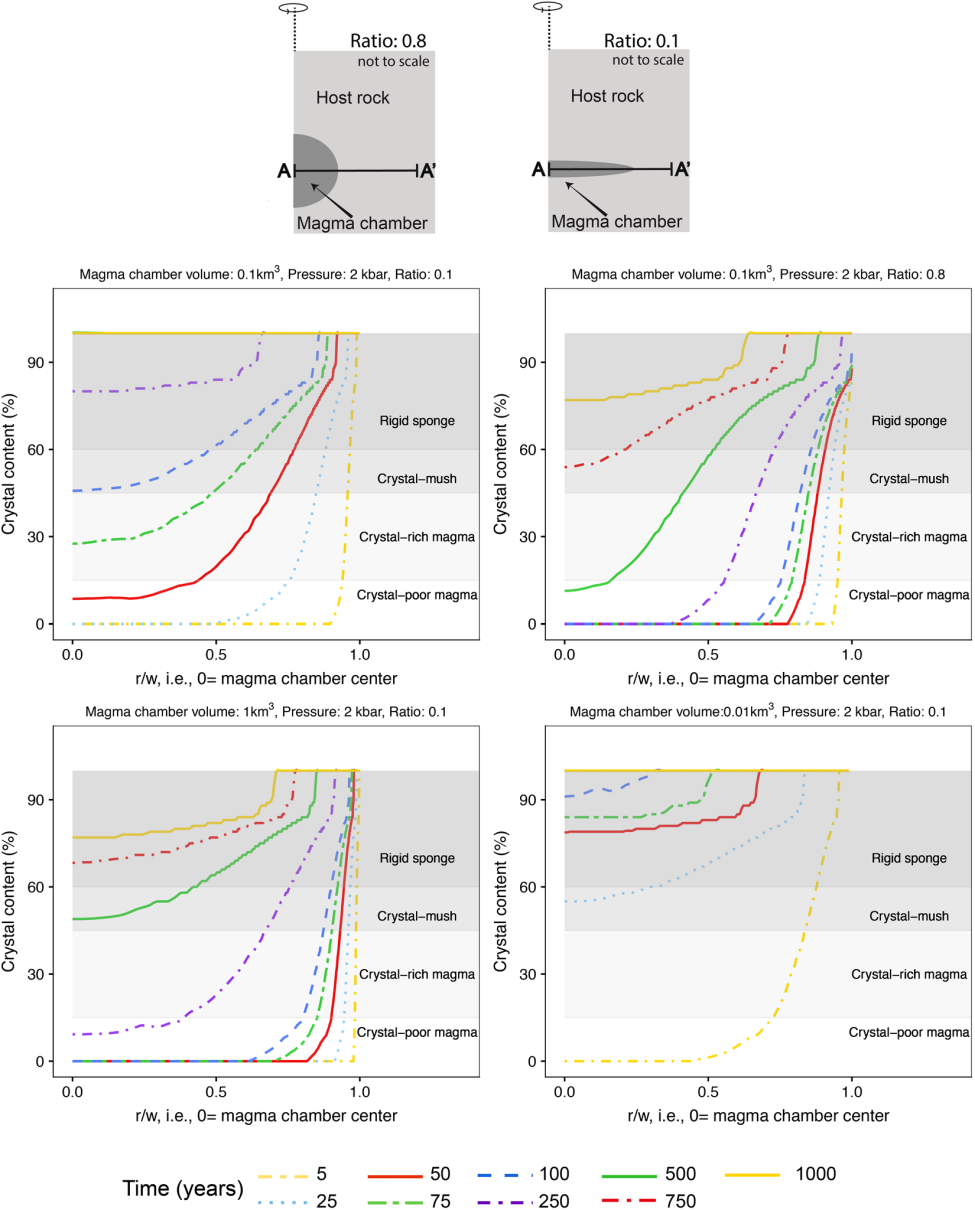
Crystal content (%) along a horizontal profile A-A′ for those models considering a magma reservoir of V = 0.01, 0.1, or 1 km^3^, R = 0.8 or 0.1, and located at different stagnation pressures P = 2 kbar. Distance r along the profile is normalized to the magma chamber width w, i.e., r/w = 1 corresponding to the contact between the magma chamber center and the host rock. This figure was generated with RStudio Version 1.0.143 (https://www.rstudio.com/) using ggplot2 package Version 2.1.9000 (http://www.ggplot2.org)^85^, a plotting system for R. Final layout of this figure was achieved using Adobe Illustrator CC 2015.3.1 (Copyright © 1987–2016 Adobe Systems Incorporated and its licensors).

Our results highlight the reservoir’s geometry, volume (*V*), and depth as the primary factors, which control the timing of the cooling process; shallow, small and sill-shaped magma pockets being the fastest to cool down (e.g.,^77,78^, Supplementary Material 9). A sill-shaped chamber of similar size to the volume of material emitted during the last DI eruption in 1970^51^ (i.e., *V* = 0.1 km^3^) located at 2 kbar pressure (i.e., similar to R3 reservoir stagnation pressure) would have hosted crystal-poor eruptible magma (i.e., magma crystal content < 15%) for at least 50 years, fully crystallizing only after a few hundred years (Fig. 7a). However, a nearly spherical reservoir of the same size and at identical depth, would contain potentially eruptible magma (i.e., crystal content < 45%) during several hundred years (Fig. 7b), similar to a sill-like reservoir of a much larger volume (*V* = 1 km^3^) (Fig. 7c). Smaller intrusions (*V* = 0.01 km^3^) are capable of retaining some eruptible magma for only few years if no further injections of fresh (and hotter) material take place (Fig. 7d). This time range can be extended for up to a few tens of years if the reservoir’s geometry is close to spheroidal (Supplementary Material 9).

Considering the depth estimated for R2 (~ 10–11 km), the caldera diameter (~ 6 × 10 km) and the general assumption that collapse calderas tend to be similar in diameter to the magma chambers responsible for the caldera-forming eruption (e.g.,^79^), we obtain a roof aspect ratio *R (R* = magma chamber depth/magma chamber diameter) for DI’s caldera in the range of 1.1–1.7. In this case, results of analogue experiments indicate that, at least, 40 to 55% (depending on *R*) of the magma contained in the chamber needs to be withdrawn to induce the caldera collapse^80^. If the ∼ 60 km^3^ of erupted magma estimated for the OCTF^36^ can be assumed to correspond to between 60–100% of the initial magma volume contained in R2 before the caldera-forming eruption, this reservoir may have still hosted up to ∼ 40 km^3^ of (non-eruptible) magma after the caldera collapse. Such an amount of material, with a crystal content between 60% and 100%, would take tens of thousands of years to cool and fully crystallize (Supplementary Material 9).

Our results lead us to interpret that the magma imaged by geophysical studies^54,75^ beneath DI corresponds to a combination of: (i) non-eruptible magma residues of the reservoir responsible for the caldera-forming event; (ii) magma remnants stagnated in chambers that developed during the post-caldera stage; and (iii) possible magma batches newly intruded in the last decades as suggested by the monitoring data recorded during the volcanic unrest episodes that happened in 1992, 1999, and 2014–2015 (e.g.,^7,8,39^). Indeed, magma plumbing systems of similar configuration have been described for other calderas worldwide such as Santorini (Greece)^68^. In these cases, shallow post-collapse dyke-fed intrusions may form laccoliths, sills, or small reservoirs, for which emplacement may drive variable amounts of reactivation of regional/local faults or caldera collapse-controlling faults (e.g. Kumano caldera, Japan)^68^. All this considered, we do not rule out the potential presence of a geothermal system as already suggested by other authors (e.g.,^36^), which may also contribute to some present-day geophysical observations up to 6 km depth.

The total amount of eruptible material beneath DI is difficult to assess due to the incapability of present geophysical techniques to identify individual magma batches but rather, only the whole picture of the island’s plumbing system. The shallow reservoirs formed after the caldera’s collapse, particularly those feeding the historic eruptions (i.e., 1820 onwards), can still host amounts of eruptible magma depending on their original size and geometry (Fig. 7). This implies that fresh and hotter magmas intruding into one of the existing chambers could easily trigger a new eruption without the requisite of creating a new magma reservoir. Hence, an eruption occurring in the future at DI may exceed the small-magma volumes of the eruptive events experienced in historical times. This highlights the necessity to perform more detailed geophysical studies on the island and its surroundings in order to improve the volcanic hazard assessment. In addition, it is important to remark that not only H_2_O as the main volatile, but also CO_2_ endowment of the magma could be a controlling factor of the past (hence future) volcanic eruption’s style and that future work should be carried out to better define the volatile budget of emitted magmas. Our conclusions reinforce the perception of DI as a very active and candidate volcano for a new eruption in the near future.

## Methodology

### Geochemistry

Geochemical data were collected from our own analytical results (Supplementary Material 9), published research works and the GEOROC database (Geochemistry of Rocks of the Oceans and Continents, http://georoc.mpch-mainz.gwdg.de/georoc/) (Supplementary Materials 2 and 3). The first data correspond to a total of 71 rock samples (Fig. 3) of different natures (incl. pyroclasts, lava flows, etc.) collected during two Antarctic campaigns carried out in the austral summers of 2010–2011 and 2012–2013 as part of the RECALDEC and PEVOLDEC projects, respectively. Major and trace elements were analysed by X-Ray Fluorescence (XRF) and Inductively Coupled Plasma–Mass Spectrometry (ICP-MS) in the GeoAnalytical Lab of at Washington State University (WSU). Additionally, major elements of mineral phases and the groundmass were analysed with an Electron Microprobe (EMP) at the Scientific and Technological Centre of Barcelona University (CCiTUB). Sr isotopic ratios were measured on twelve selected samples (Supplementary Material 1) using an IsotopX Phoenix Thermal Ionization Mass Spectrometer (TIMS) at the Centro de Geocronología y Geoquímica Isotópica, Universidad Complutense de Madrid, Spain). Analytical techniques and data processing are detailed in Supplementary Materials 5 and 6.

### Pressure-Temperature (P-T) estimates

They were calculated using (i) X-Ray Fluorescence (XRF) and Electron Microprobe (EMP) data from our own samples (Supplementary Material 1) and (ii) rhyolite-MELTS software v.1.2.0^72–74^ to analyse the thermodynamic database(http://melts.ofm-research.org/). The latter accounts for the phases and residual glass(es) involved in equilibrium crystallization during magma cooling. The range of water content we applied to the input compositions varies from 0.1 to 2%. Further methodological details on P-T condition estimates can be found in Supplementary Material 5.

### Linear Discriminant Analysis

LDA is a supervised classification method that uses expert-defined groups (i.e., the magmatic sources defined in this study: M, R1–R6) in a sub-set of cases (“training subset”) to calculate a linear transformation of the descriptors in order to maximize discrimination among groups. This transform is subsequently applied to all data, which are then classified into the groups. As a machine-learning method, LDA is very sensitive to the correctness and typicality of group adscriptions in the training phase and we have used this characteristic to check the consistency of our conceptual grouping. A first run of LDA can be used to point out incorrectly ascribed or atypical samples within their respective groups. Once user-defined adscriptions in the training set are certain, LDA can be used to assess whether the grouping defined by the training set is consistent in terms of the descriptor variables for the rest of the samples. Further methodological details on the performed LDA can be found in Supplementary Material 8.

### Fractional crystallization models

Modelling with rhyolite-MELTS software v.1.2.0^72–74^ was performed to test the consistency of DI’s compositional trends for magma differentiation through the fractional crystallization processes. Differentiation by fractional crystallization of starting compositions in the mafic (e.g., B.751.5a from Smellie *et al*.^3^) and intermediate (e.g., DI-4SUP) compositional areas of the studied magmas was modelled. This was done for different ranges of initial H_2_O content (0–1.25 wt.%), pressures (1–5 kbar), and both fO_2_ conditions—0 to 2 fixed log units above the Quartz-Fayalite-Magnetite (QFM) buffer). Modelling was performed with fO_2_ conditions both fixed and free relative to the QFM buffer during calculations. Starting compositions, which were consistent with all major elements (no outlier positions for any element) were chosen among those in the mafic and intermediate areas within the differentiation trends. Further methodological details on the performed fractional crystallization models can be found in Supplementary Material 8.

### Magma chamber cooling models

The internal temperature distribution of the magma chamber is calculated using the Finite Element (FE) method, by solving the heat transfer equation by conduction, assuming as negligible the effect of viscous heating and pressure-volume work. The geometric modelling, mesh discretization and numerical computations were carried out with COMSOL Multiphysics v5.2a software package (http://www.comsol.com). The performed FE models are axisymmetric and were constructed over a cylindrical coordinate system with positive *z* values related to altitudes above sea level. The magma chamber geometry is oblate in shape with height *h* and width *w*. The selected starting magmatic compositions for the numerical simulations correspond to the sample DI-4SUP). The melt (θ) and solid (ϕ) fractions, as well as the thermal properties of the crystallizing magmas, are determined using the rhyolite-MELTS software v.1.2.0^72–74^. Further methodological details on the performed magma chamber cooling models can be found in Supplementary Material 8.

## Electronic supplementary material

Supplementary Material 1

Supplementary Material 2

Supplementary Material 3

Supplementary Materials 4–9
