# Supplementary material for: Deciphering the evolution of Deception Island’s magmatic system
Source: Sci Rep. 2019 Jan 23;9:373. doi: 10.1038/s41598-018-36188-4 (PMC6344569; doi:10.1038/s41598-018-36188-4)
Supplement: Supplementary file 4 — Supplementary Materials 4–9 [file 41598_2018_36188_MOESM4_ESM.pdf]

Deciphering the evolution of Deception Island's magmatic system

A. Geyer (1), A.M. Álvarez-Valero (2), G. Gisbert (3), M. Aulinas (4), D. Hernández-Barreña (2), A. Lobo (1), J. Marti (1)

(1) Institute of Earth Sciences Jaume Almera, ICTJA,CSIC, Lluis Sole i Sabaris s/n, 08028 Barcelona, Spain

(2) Departamento de Geología, Universidad de Salamanca, 37008 Salamanca, Spain

(3) Instituto de Geociencias, CSIC-UCM, Severo Ochoa 7, 28040 Madrid, Spain

(4) Departament de Mineralogia, Petrologia i Geologia Aplicada. Universitat de Barcelona, Marti Franques s/n, 08028 Barcelona, Spain

SUPPLEMENTARY MATERIAL 4

Petrographic features of the volcanic samples from Deception Island

The studied rock samples were collected during the 2010–2011 and 2012–2013 Antarctic campaigns as part of the RECALDEC and PEVOLDEC projects, respectively (Supplementary Material 1). Pre-caldera products consist mainly of (i) porphyritic lavas of basalts and basaltic andesites with fine grain phenocrysts (up to 1 mm) of euhedral to subeuhedral olivine (Ol), pyroxene (Px), and plagioclase (Pl; locally displaying twining and zonation) within a groundmass of mainly microlites of the same mineralogy, and Fe-Ti oxides (Fig. S4-1a); (ii) palagonitized scoria and hyaloclastite breccia with scarce subeuhedral phenocrysts of Ol, Px, and Pl within a microcrystalline-glassy groundmass (Fig. S4-1b); and (iii) vitric scoria pyroclasts as lapilli tuffs and minor bombs, which are characterised by abundant palagonitic alteration and variable volume of vesicles (Fig. S4-1c).

Syn-caldera products are mainly composed of welded ignimbrite and lithic breccia with scoriaceous material and lithic fragments of pre-caldera juveniles (locally with platy shards and Px, Pl fragments, Fig. S4-2) embedded within a fine-grained pyroclastic yellowish glassy matrix (Fig. S4-2). The yellow colour of the matrix is related to the later palagonitic alteration, which developed from the pale brown basaltic (sideromelane) glass. Baker et al.[1] suggested that this process is time-dependent and may explain the distinction between the older yellow rocks and the younger, post-caldera, brown pyroclastic rocks. The scattered distribution of the trapped pre-caldera fragments (mostly black) shows no signs of alteration.

Post-caldera eruptions ejected (i) basaltic lavas with microlites of mainly Pl, typically euhedral, zoned, locally grouped, and displaying polysynthetic and Carlsbad twining, as well as abundant glass and mafic (mainly Px) inclusions. In places, plagioclase microlites show trachytic and pilotaxitic textures, oriented following the melt direction flow. Pyroxenes are euhedral and subeuhedral phenocrysts showing a wide variation in size. Locally, they appear both between and within plagioclase phenocrysts (intergranular and sieve textures). Olivines are made up of scarce euhedral crystals that are typically fractured/broken with straight extinction; (ii) scoria of fine-grained glassy groundmass (typical aspect of vitric tuff), with variable vesicle quantity and shapes (locally infilled by fibrous zeolite) (Fig. S4-3). They also host local lithic

fragments of mafic material (lava, scoria) and ignimbrites. In thin section, the aphanitic glassy groundmass contains minor crystals (up to 10% per vol.), which are mainly Pl microlites (needle-shaped laths) and euhedral Px (mainly Cpx, minor Opx), and local small (<< 1 mm) phenocrysts of Ol and Px, and minor larger phenocrysts of euhedral to subeuhedral Pl (locally displaying twining and zonation) (Fig. S4-3). Microlites may be locally aligned in a flow texture that wraps around phenocrysts, yet they typically show a random distribution. The glassy groundmass shows significant variability, ranging from totally fresh/unaltered to devitrified/altered, i.e., from basaltic sideromelane (quenched glass, clear and isotropic) transforming to palagonite (yellowish-orange) or totally devitrified (black and isotropic). The scoriaceous material also includes variable amounts and types of lithics such as lavas, pumice, ignimbrite, and oxidized scoria. The minor, more evolved/acidic samples, pumice and scoria fragments show glassy groundmasses that are normally devitrified or altered.

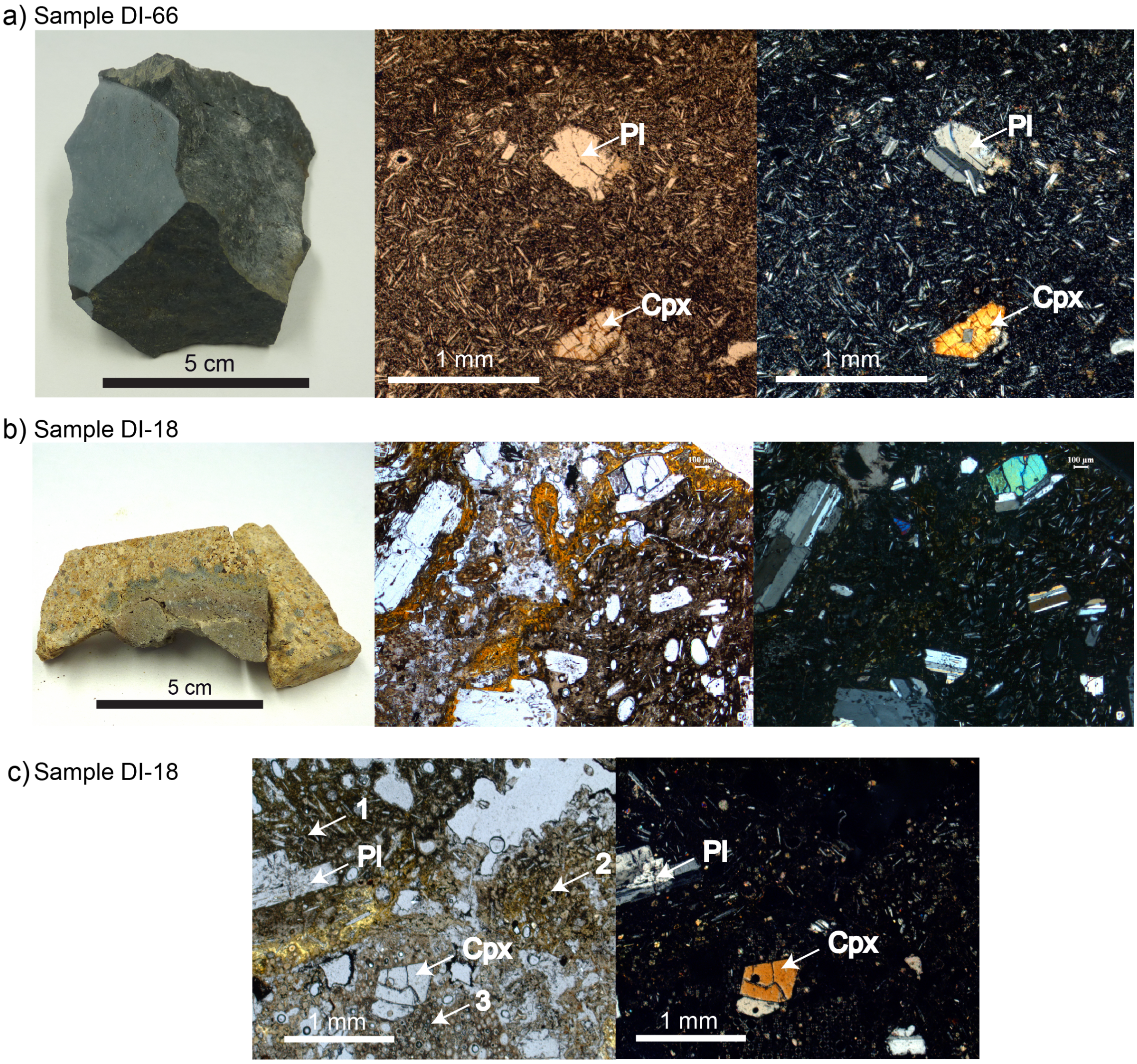

Fig. S4-1: Hand-specimen (left) and plane- and cross-polarized light microscopy images (center and right, respectively) of: a) a pre-caldera porphyritic lava with Clinopyroxene (Cpx) microphenocrysts in a moderately fluidal groundmass of Plagioclase (Pl) microlites and Cpx and oxides microcrystals; b) fragmented hyaloclastite view of a pre-caldera sample with palagonite alteration; and c) a pre-caldera cohesive lapilli-tuff. Arrows 1-3 point out three vesiculated porphyritic pyroclasts cohered by a colorless isotropic thin film as a cement. Phenocrysts are Pl and Cpx.

Sample DI-28

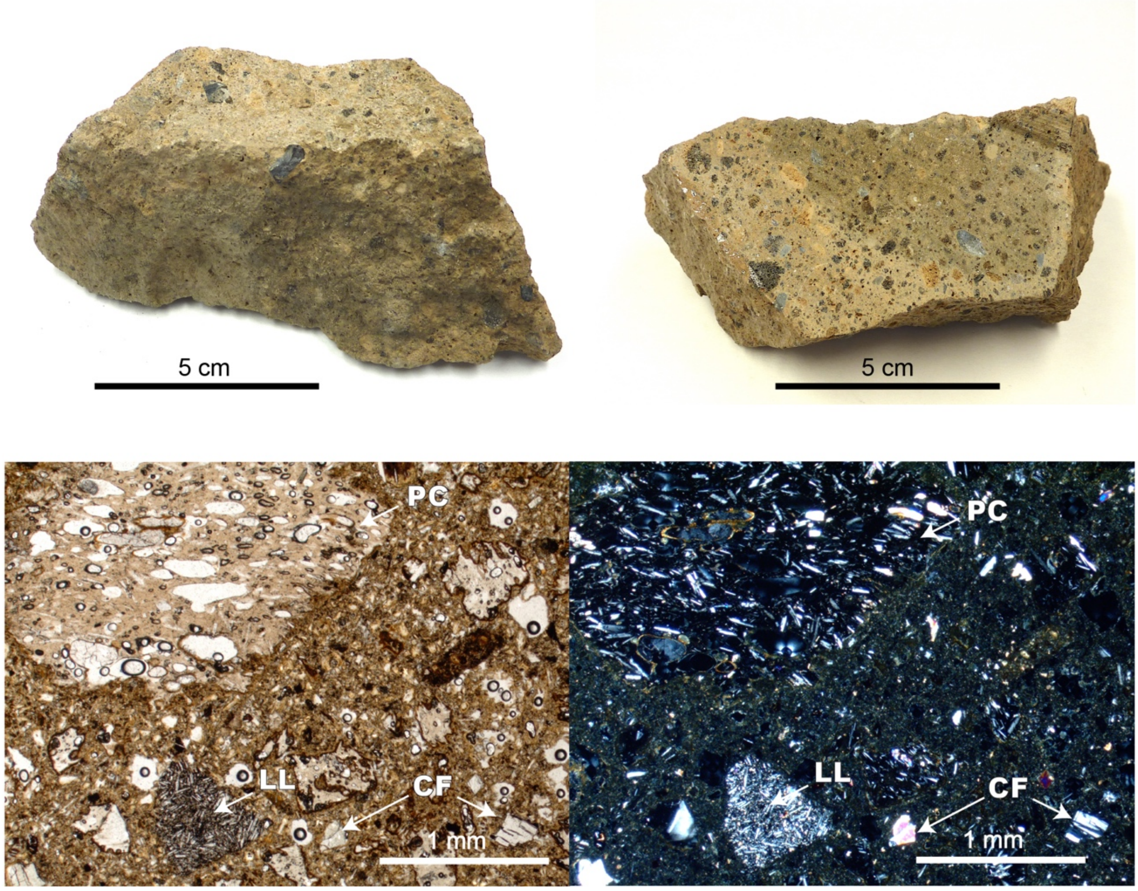

Fig. S4-2: Hand-specimen and microscopy images (plane- and cross-polarized) of a syn-caldera ignimbrite. Lapilli-tuff with a partly palagonitized fine ash matrix bearing pumiceous juvenile pyroclasts (PC), lava lithic clasts (LL), and crystal fragments (CF). The highly vesicular clast PC has aligned Pl microlites and Cpx microcrystals in a light brown glassy groundmass.

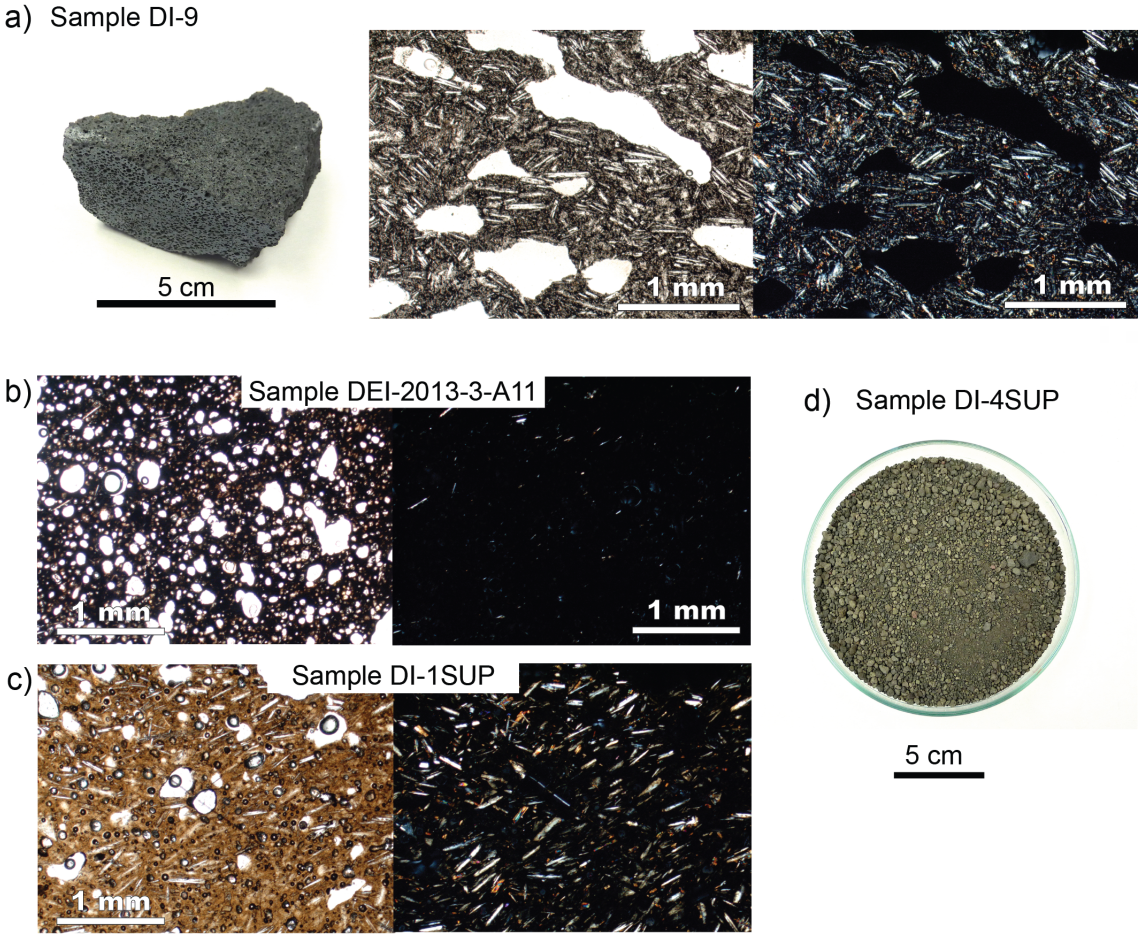

Fig. S4-3: Hand-specimen (left) and plane- and cross-polarized light microscopy images (center and right, respectively) of: a) post-caldera lava dike (Sample DI-9) showing vesicles with fluidal texture marked by moderately aligned Pl microlites in a matrix also containing Cpx and opaques; b) vesicular juvenile pyroclast with Pl and Cpx microlites in a light brown glassy matrix (Sample DI-1SUP); c) highly vesicular, nearly aphyric, scoriaceous juvenile pyroclast. Scarce Pl microphenocrysts and Pl and Cpx microcrysts (Sample DEI-2013-3-A11); d) Image of pyroclastic material erupted during the post-caldera phase (Sample DI-4SUP).

References

1 Baker, P. E., McReath, I., Harvey, M. R., Roobol, M. J. & Davies, T. G. The geology of the south Shetland islands: Volcanic evolution of Deception island. *British Antarctic Survey Scientific Reports* **78**, 81 pp. (1975).

Deciphering the evolution of Deception Island's magmatic system

A. Geyer (1), A.M. Álvarez-Valero (2), G. Gisbert (3), M. Aulinas (4), D. Hernández-Barreña (2), A. Lobo (1), J. Martí (1)

(1) Institute of Earth Sciences Jaume Almera, ICTJA, CSIC, Lluis Sole i Sabaris s/n, 08028 Barcelona, Spain

(2) Departamento de Geología, Universidad de Salamanca, 37008 Salamanca, Spain

(3) Instituto de Geociencias CSIC-UCM, Severo Ochoa 7, 28040 Madrid, Spain

(4) Departament de Mineralogia, Petrologia i Geologia Aplicada. Universitat de Barcelona, Marti Franques s/n, 08028 Barcelona, Spain

SUPPLEMENTARY MATERIAL 5

Geochemistry of volcanic products at Deception Island

Data compilation and processing

The geochemical database of Deception Island's rock samples used in this study was compiled from an exhaustive integration of both our own analytical results (Supplementary Material 1) and a compilation of published research[1-10](Supplementary Material 2). The former correspond to a total of 71 rock samples of different natures (incl. pyroclastic material, lava flows, etc.) collected during two Antarctic campaigns carried out in the austral summers of 2010–2011 and 2012–2013 as part of the RECALDEC and PEVOLDEC projects, respectively. All samples were classified as belonging to the pre-, syn- or post-caldera stage based on the synthetic stratigraphy presented by Martí et al. [11] (Fig. S5-1).

Whole rock major and trace element compositions were analysed for our samples by X-Ray Fluorescence (XRF) using a ThermoARL Advant'XP+ sequential X-ray spectrometer at the GeoAnalytical Lab at Washington State University (WSU) (https://environment.wsu.edu/facilities/geoanalytical-lab/) (Supplementary Material 1). Loss on Ignition (LOI) as a proxy for volatile content was measured using standard thermogravimetric methods at WSU. Trace elements and Rare Earth Elements (REE) were also analysed on the same samples by Inductively Coupled Plasma - Mass Spectrometry (ICP-MS) using an Agilent 7700 at WSU. Whole rock powders were dissolved following a fusion-dissolution method that efficiently decomposes refractory mineral phases and removes the bulk of unwanted matrix elements. The procedure consists in a low-dilution fusion with di-Lithium tetraborate followed by an open-vial mixed acid digestion (HNO_3 + HF + $HClO_4$). Relative precision was in general better than 1% (RSD) for major oxides and trace elements, under 5% for REE, and under 10% for the remaining trace elements.

Additionally, major element glass and mineral chemistry was analysed on thin sections of a selection of samples using a JEOL JXA-8230 electron microprobe at the Scientific and Technological Centre of Barcelona University (CCiTUB) (http://www.ccit.ub.edu/EN/home.html) (Supplementary Material 1). A combination of silicates

and oxides was used for calibration. An up to 20-μm-diameter defocused beam was used for groundmass analyses in order to minimise Na migration.

The geochemical database (Supplementary Materials 1 and 2), shows only results of samples with (i) structural volatile content (LOI, H_2O^+) lower than 2.5 wt.% (rocks with higher content are more likely to have undergone alteration processes, which may have modified their original mobile element compositions[12]); and (ii) the sum of major elements plus volatiles ranges from 98 to 101 wt.%.

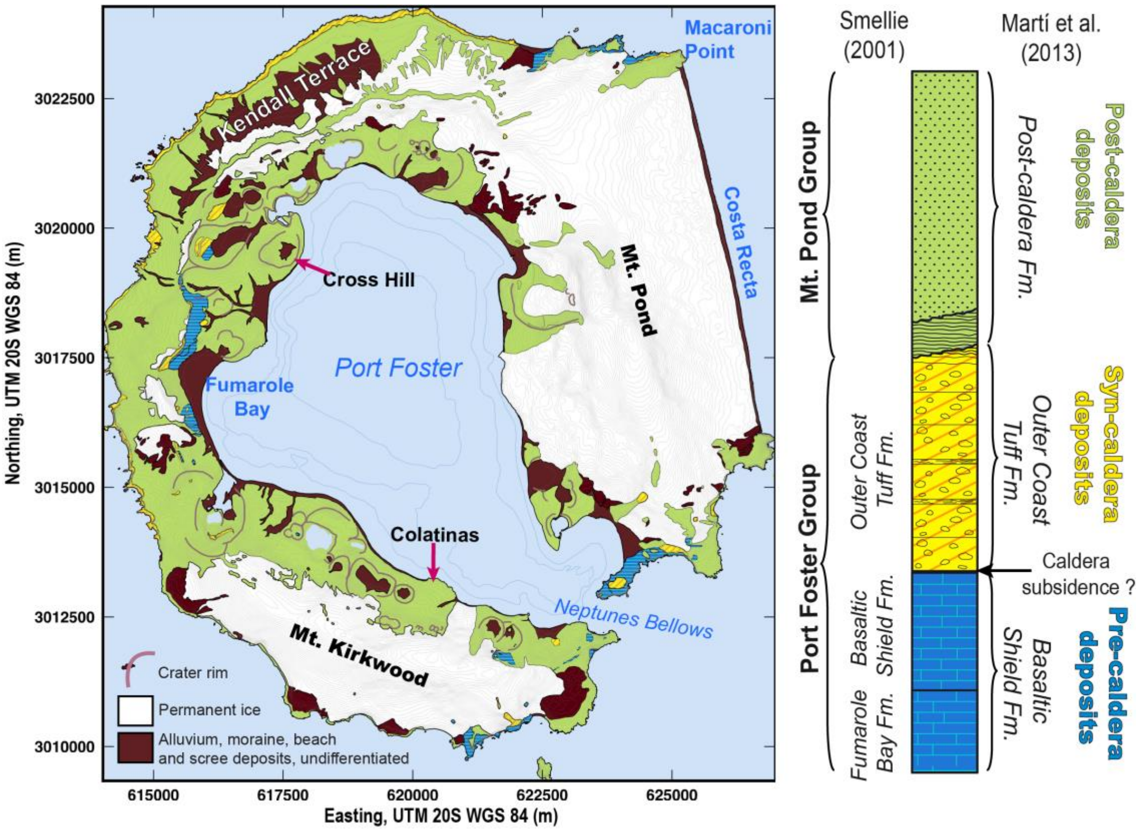

Fig. S5-1: Simplified geologic map (left) and stratigraphic section (right) of Deception Island (both modified from Martí et al. [11]). At the sides of the stratigraphic log divisions proposed in previous studies are indicated. Data obtained from Spatial Data Infrastructure for Deception Island SIMAC, Torrecillas et al.[13]. This figure was generated with QGIS software version 2.18 Las Palmas (available at: www.qgis.org). Final layout was obtained with Adobe Illustrator CC 2015.3.1 (Copyright © 1987–2016 Adobe Systems Incorporated and its licensors).

Whole rock geochemistry

Rocks in Deception Island range in composition from basalts to trachydacites and rhyolites, and follow a distinctive alkalinity-increasing trend at the upper end of the subalkaline field in the Total Alkali vs. Silica diagram (TAS, Fig. S5-2). This trend shows an apparent compositional gap at c. 63–66 wt.% SiO_2 that separates the highly evolved samples from the Cross Hill and Colatinas areas (Fig. S5-1). Overall, Deception Island (DI) rocks show (Fig. S5-3): a negative correlation of MgO, Al_2O_3, CaO, and a positive correlation of K_2O with SiO_2; a positive correlation of FeO_t and TiO_2 with SiO_2 between 50 and 55 wt.% SiO_2, followed by a negative correlation; and a positive correlation of Na_2O and P_2O_5 with SiO_2, with depletion at the silica-rich end of the trend (SiO_2 > 65 wt.%). Rocks of basic compositions (< 55 wt.% SiO_2) from pre- and post-caldera stages show a significant chemical dispersion around an inferred average trend, which is most evident in TiO_2 and FeO_t vs SiO_2 but also visible in the other Harker diagrams. On the other hand, most syn-caldera rocks define trends, which differ from those outlined by pre- and post-caldera samples.

The syn-caldera rocks define two geochemically distinctive groups: i) a main compositional cluster that comprises most of the samples deviating from the principal chemical trends. This corresponds to the proposed second magma by Smellie et al. [6]; and ii) a second cluster within the main DI geochemical trends composed of minor samples with < 55 wt.% SiO_2. The syn–caldera samples of the main cluster group at lower TiO_2 and FeO_t values and for the same SiO_2 content, have slightly higher concentrations of MgO, Al_2O_3, CaO, and lower Na_2O and K_2O than the pre- and post-caldera rocks in major elements vs. SiO_2 Harker diagrams (Fig. S5-3).

Most trace elements show a positive correlation with SiO_2 except for Cr, Ni, Sc, Sr (Fig. S5-4). Vanadium behaviour is equivalent to that of TiO_2 and FeO_t, with the rocks of the main cluster group also depicting compositional differences relative to the pre- and post-caldera rocks. REE contents depict nearly straight trends in a chondrite-normalized diagram (Fig. S5-5), with minor negative Eu anomalies in the most evolved rocks, yet positive in some mush samples (Figs. S5-5

and S5-6). In contrast, marked positive (Cs and Pb) and negative (Nb) anomalies occur in the primitive mantle-normalized diagram. There are no significant differences among pre-, syn-, and post-caldera rocks in chondrite- and primitive mantle-normalized diagrams.

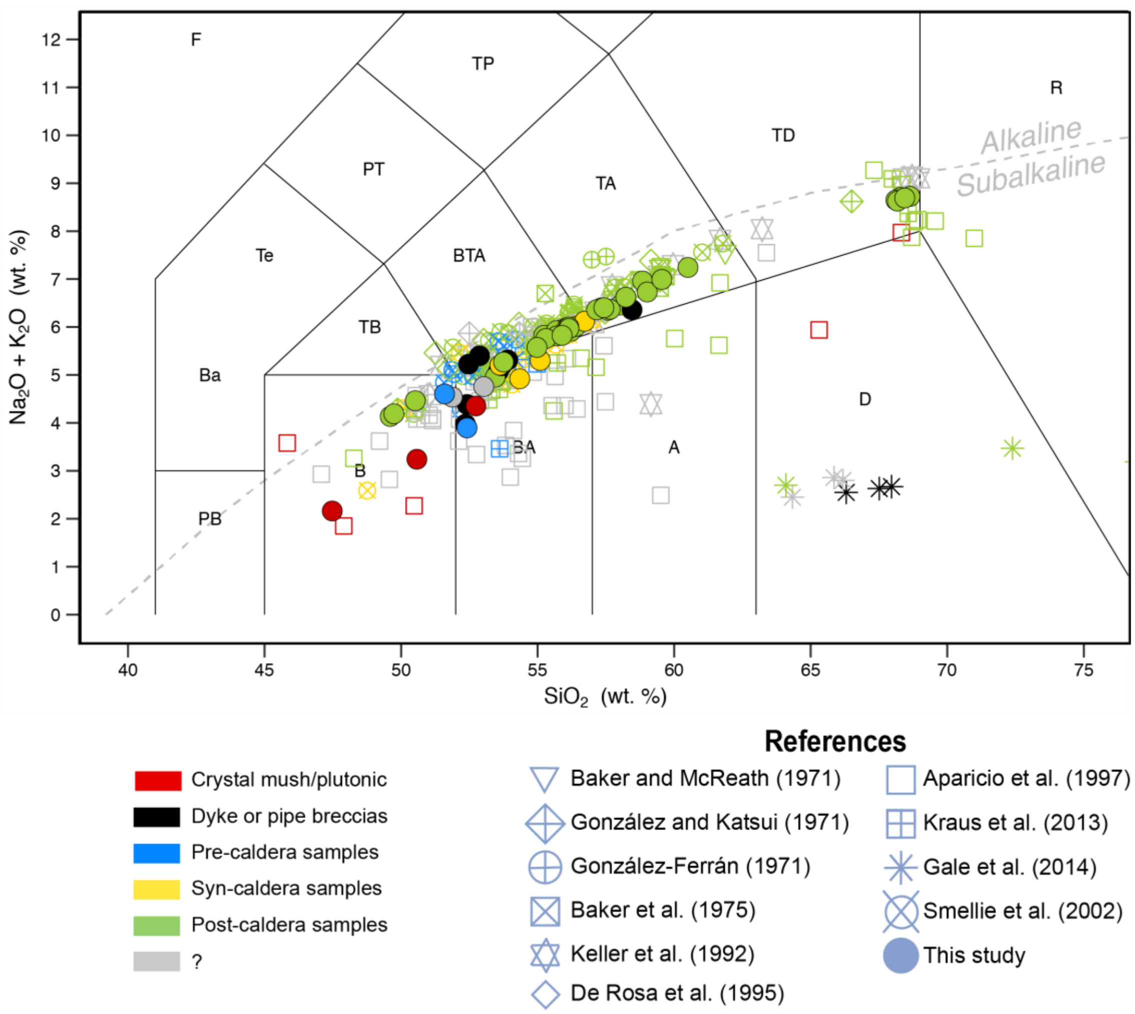

Fig. S5-2: Total Alkali vs. Silica diagram (TAS)[14] for the rock samples considered in this work (see Supplementary Materials 1-2 for details on composition and exact latitude-longitude coordinates of the rock samples). Major elements normalized to 100% (anhydrous) with Fe distributed between FeO and Fe_2O_3 following Middlemost[15]. Grey dashed line discriminates between the alkaline-subalkaline fields[16]. This figure was generated with RStudio Version 1.0.143 (https://www.rstudio.com/) using ggplot2 package Version 2.1.9000 (http://www.ggplot2.org), a plotting system for R. Final layout of this figure was achieved using Adobe Illustrator CC 2015.3.1 (Copyright © 1987–2016 Adobe Systems Incorporated and its licensors).

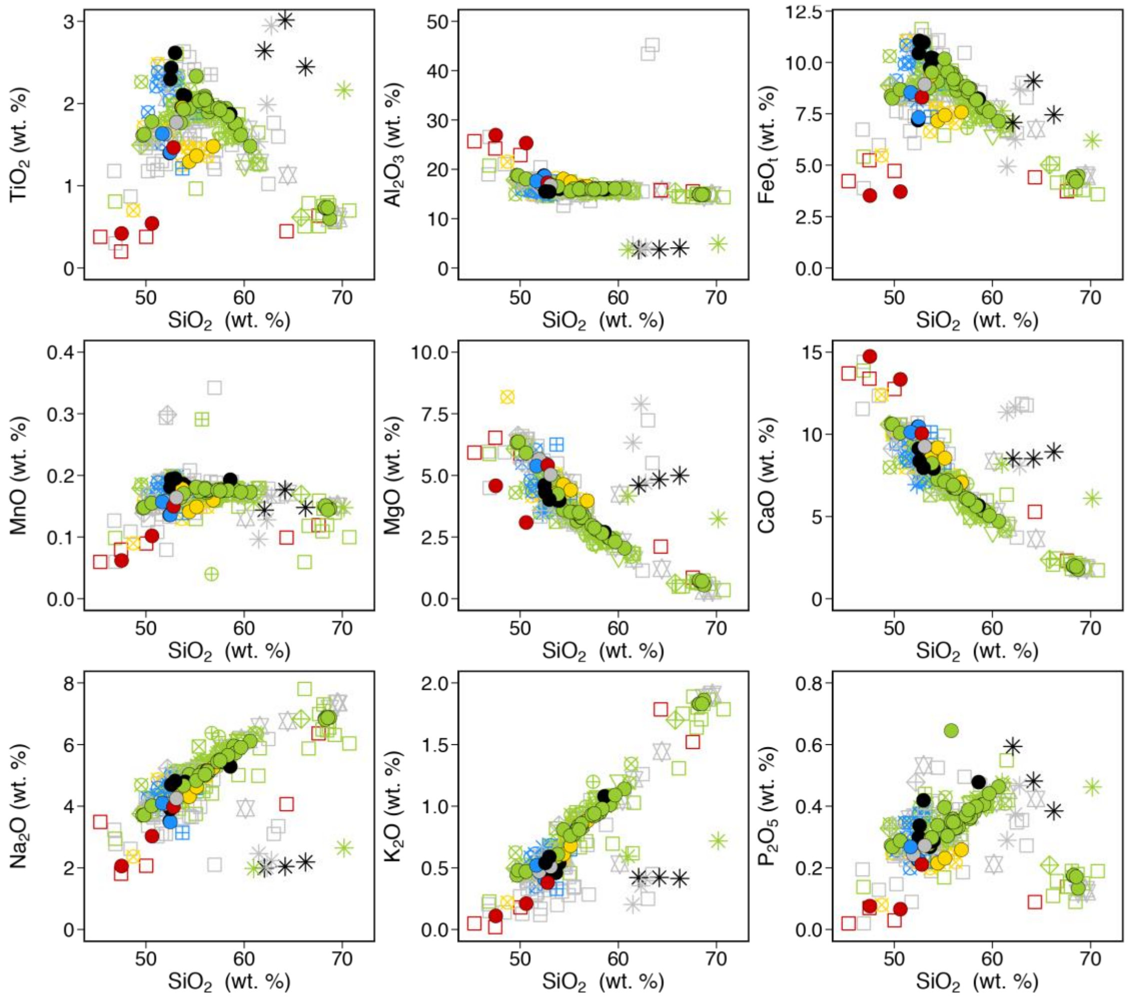

Fig. S5-3: Major elements vs. SiO_2 content Harker Diagrams for the rock samples considered in this work see Supplementary Materials 1-2 for details on composition and exact latitude-longitude coordinates of the rock samples). Major element compositions have been normalized to 100% in anhydrous base with Fe as FeOt. This figure was generated with RStudio Version 1.0.143 (https://www.rstudio.com/) using ggplot2 package Version 2.1.9000 (http://www.ggplot2.org), a plotting system for R. Legend as per Fig. S5-2.

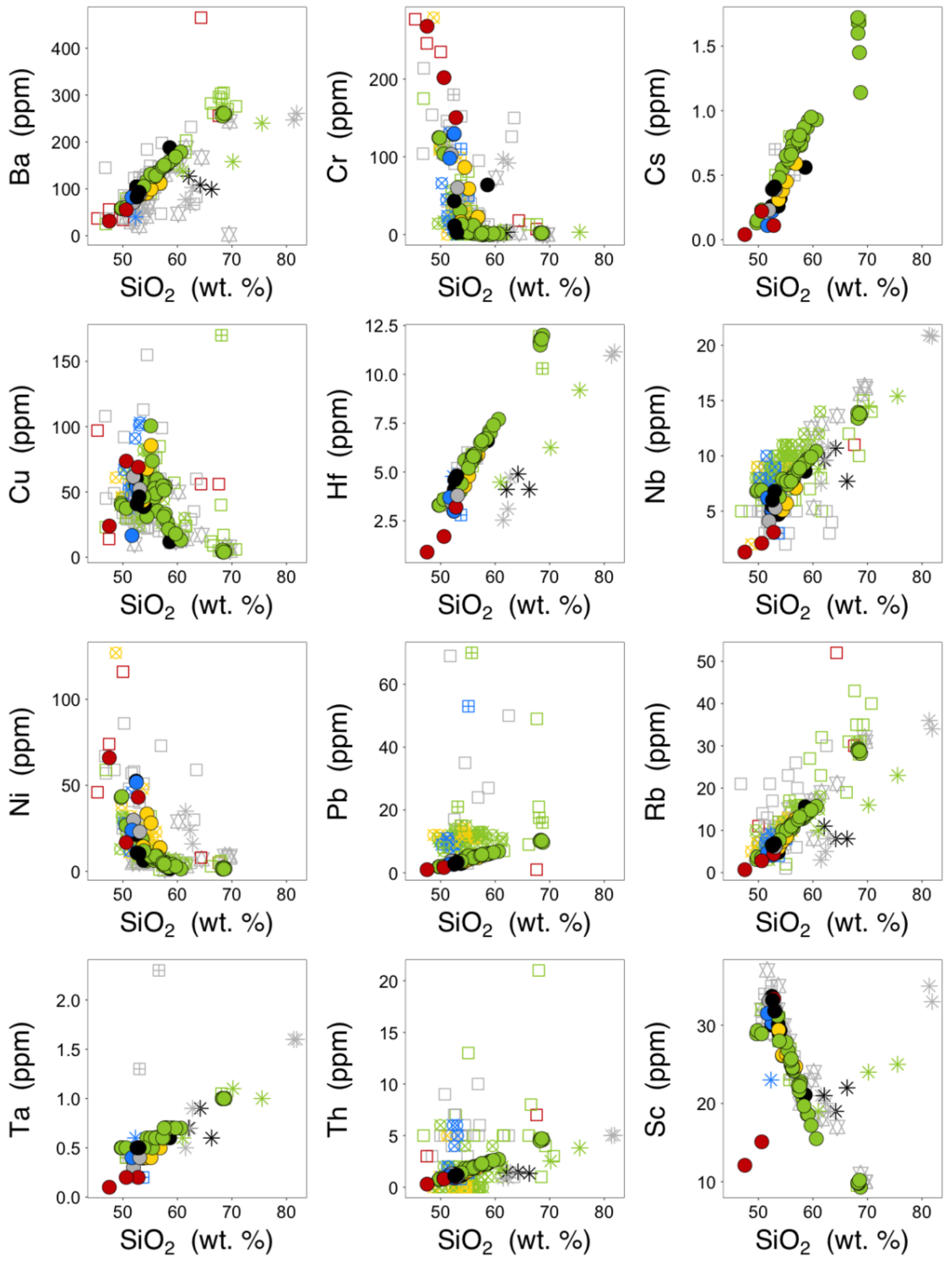

Fig. S5-4: Trace and Rare Earth elements vs. SiO_2 content Harker Diagrams for the rock samples considered in this work (see Supplementary Materials 1-2 for details on composition and exact latitude-longitude coordinates of the rock samples). SiO_2 contents are taken from major element compositions normalized to 100% in anhydrous base with Fe as FeO_t. This figure was generated with RStudio Version 1.0.143 (https://www.rstudio.com/) using ggplot2 package Version 2.1.9000 (http://www.ggplot2.org), a plotting system for R. Legend as per Fig. S5-2.

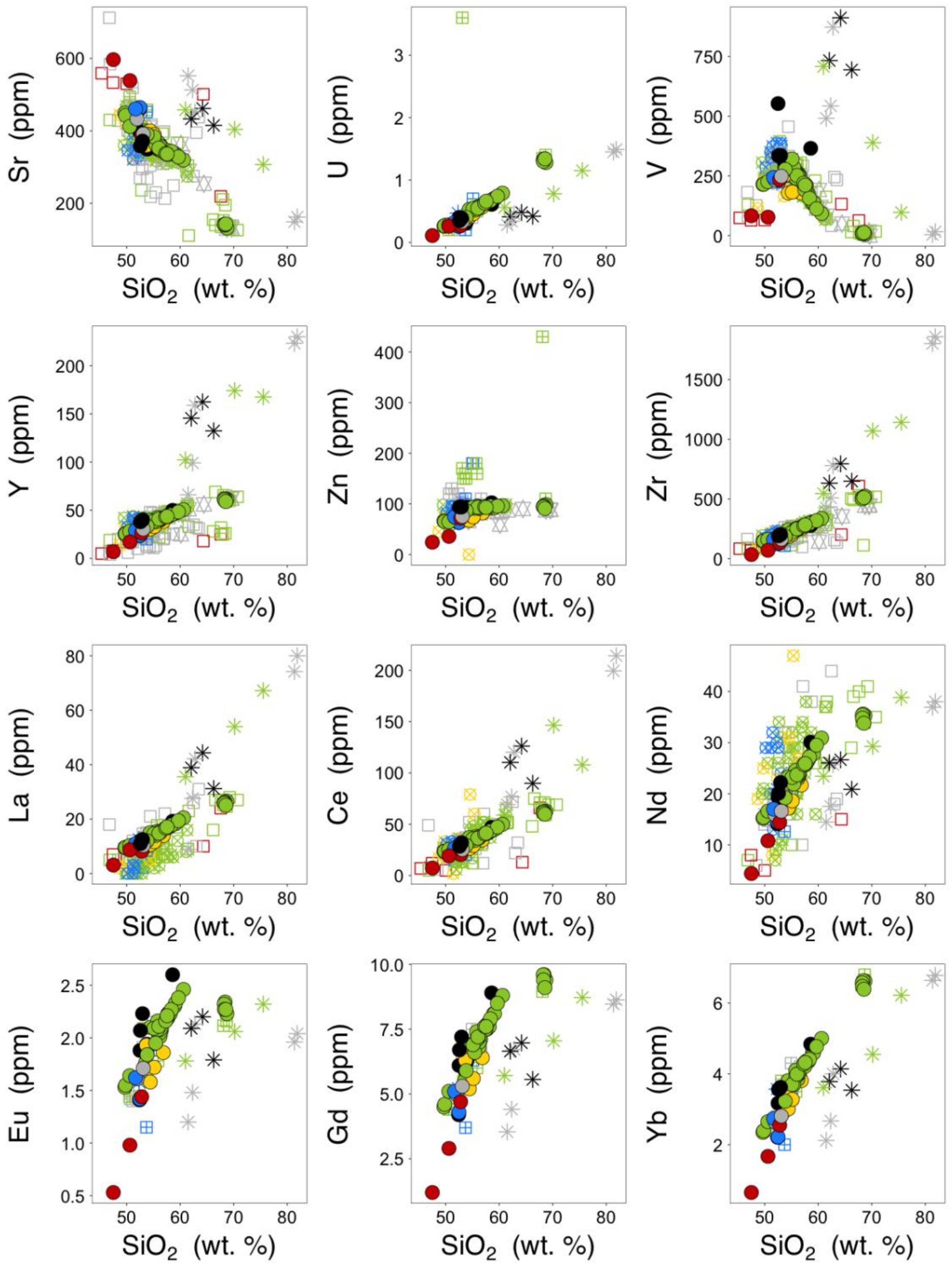

Fig. S5-4: continuation

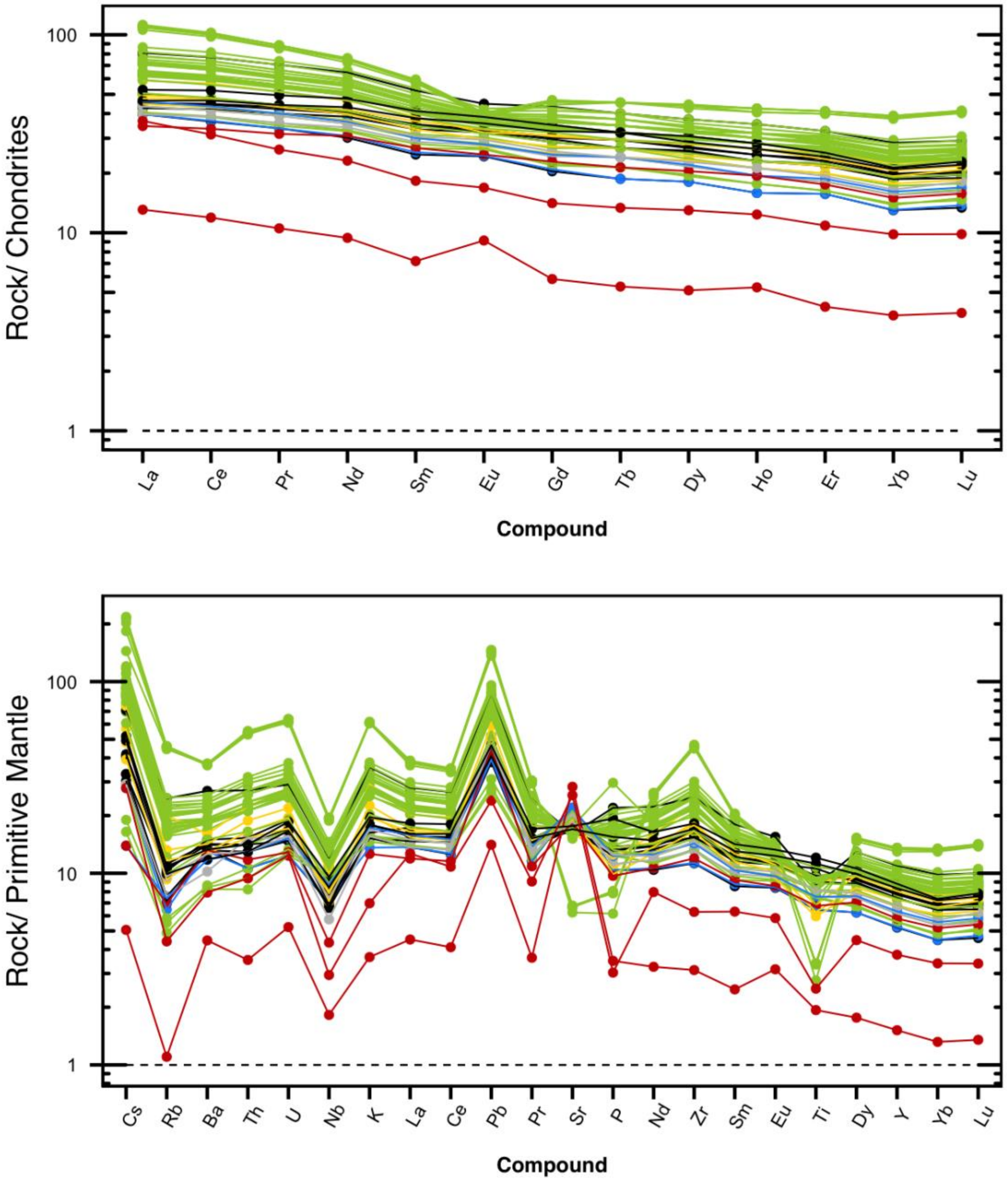

Fig. S5-5: Whole Rock compositions normalized to chondrites (top) and primitive mantle (bottom) compositions of Sun and McDonough[17]*. Major element compositions have been normalized to 100% in anhydrous base with Fe as* FeO_t*. This figure was generated with RStudio Version 1.0.143 (https://www.rstudio.com/) using ggplot2 package Version 2.1.9000 (http://www.ggplot2.org), a plotting system for R. Legend as per Fig. S5-2.*

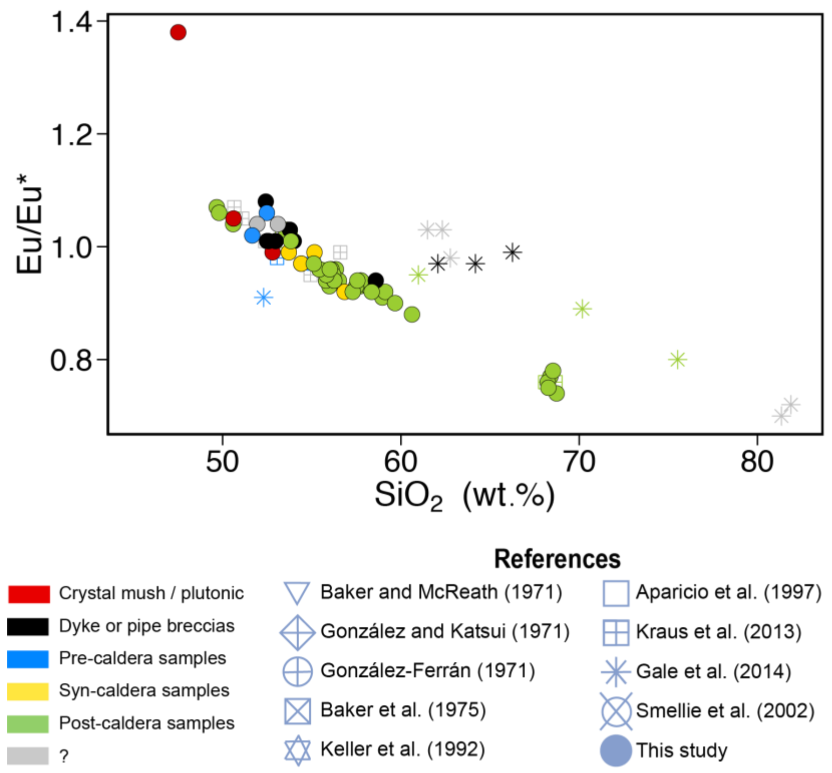

Fig. S5-6: Eu/Eu vs. SiO_2. SiO_2 contents are taken from major elements compositions normalized to 100% in anhydrous base with Fe as FeO_t. This figure was generated with RStudio Version 1.0.143 (https://www.rstudio.com/) using ggplot2 package Version 2.1.9000 (http://www.ggplot2.org), a plotting system for R. Legend as per Fig. S5-2.*

Glass and mineral geochemistry

Mineral chemistry

The main mineral assemblage of DI rocks is composed of olivine (Ol), pyroxene (Px), plagioclase (Pl), and Fe-Ti oxide, as well as a significant variety of glasses (Supplementary Material 4).

Olivine

Olivine crystals show a wide range of forsterite content (from Fo_{36} to Fo_{88}) (Fig. S5-7). This variation was already noted by Weaver et al.[18] and Aparicio et al.[5] and is related to magmatic differentiation. The highest contents (> 70% Fo) mainly correspond to phenocrysts and microphenocrysts of basaltic samples. The lowest forsteritic Ol (Fo_{55-70}) are essentially found as microcrysts and microphenocysts of trachyandesites, basaltic trachyandesites, trachybasalts and minor basalts. The most fayalitic olivines (Fo_{36}) occur in the most evolved trachytes. Nickel concentrations generally decrease in line with decreasing Fo values due to magma fractionation, especially for Fo contents above 80% (Fig. S5-7). The increase in Ca with decreasing Fo is also related to magma fractionation (while Ni is more compatible in Ol, Ca preferentially partitions into Px). Systematically, Ol belonging to crystal mush and pipe breccia show Fo contents above 80%, whereas the syn-caldera and post-caldera Ol show a wider range of compositions (Fig. S5-7).

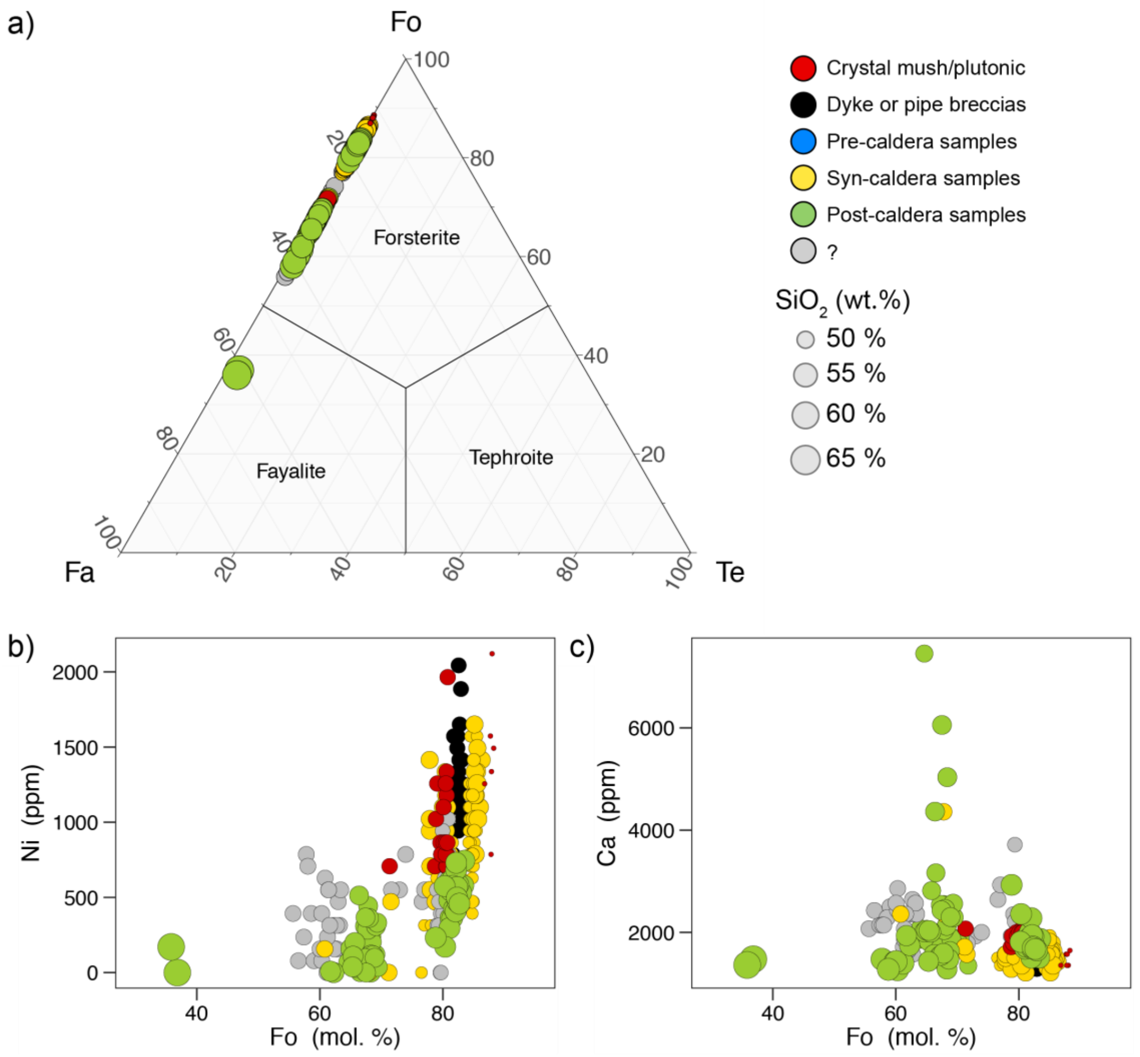

Fig. S5-7: a) Forsterite (Fo) - Fayalita (Fa) - Tephroite (Te) ternary diagram for olivine classification; b) Ni vs. Fo content in olivine; c) Ca vs. Fo content in olivine. SiO_2 symbols refer to the silica content of rocks sample containing the analysed olivine crystal (see Supplementary Material 1 for details on composition and exact latitude-longitude coordinates of the rock samples). This figure was generated with RStudio Version 1.0.143 (https://www.rstudio.com/) using ggplot2 package Version 2.1.9000 (http://www.ggplot2.org) and ggtern package Version 2.2.1 (http://www.ggtern.com/), plotting systems for R. Final layout of this figure was achieved using Adobe Illustrator CC 2015.3.1 (Copyright © 1987–2016 Adobe Systems Incorporated and its licensors).

Pyroxene

Most samples are clinopyroxenes (Cpx) and are mainly represented in the diopside and augite fields within the Morimoto et al. (1989)[19] quadrilateral diagram (Fig. S5-8). They show compositions of $Wo_{45.0-46.4}$, $En_{40.5-47.8}$, $Fs_{6.5-13.9}$ for diopsides and $Wo_{22.0-44.9}$, $En_{32.9-51.6}$, $Fs_{8.2-36.3}$ for augites. Whereas the Mg-number [Mg#; 100 x MgO/ (MgO + FeOt) mol %] shows a narrow range in diopsides (81–89%), it is variable in the case of augites (from 54 to 88%). The latter also show variable TiO_2 contents (0.25 to 3.08 wt.%) being those with TiO_2 above 3 wt.% and corresponding to titanaugites. Rare aegirine-augites are also present in some samples characterized by higher Na_2O contents (2.63 to 4.87 wt.%) and Al_2O_3 (14.86–17.69 wt.%), and lower CaO (7.61–8.67 wt.%). Diopsides are only identified in basalts and minor trachybasalts, whereas augite is present across a wider compositional spectrum. Augites with Mg# above 75% are mainly found in basalts and trachybasalts, while those with Mg# between 65 and 75% correspond to basaltic trachyandesites and trachyandesites, evidencing magmatic differentiation. Augites in trachytes present Mg# below 65%. The magmatic differentiation is also detected by the decrease in Ca or the increase in Fe with decreasing Mg#. Cpx belonging to the pre-caldera and mush samples, show a narrow range in Mg# (79–86% and 73–80%, respectively), whereas the syn- caldera (Mg# 75–88%) and especially, the post-caldera Cpx (Mg# from 54 to 80%) show a larger range in compositions. On the other hand, Opx are enstatites ($Wo_{2.5-4.0}$, $En_{46.9-75.7}$, $Fs_{21.3-49.4}$) (Fig. S5-8). Their presence is restricted to two samples of the crystal mush (DI-67 and DI-70), one syn-caldera basalt (DI-36), and two trachytes (DI-24 and DI-43) belonging to the post-caldera period (see also Weaver et al.[18] and Aparicio et al.[5]).

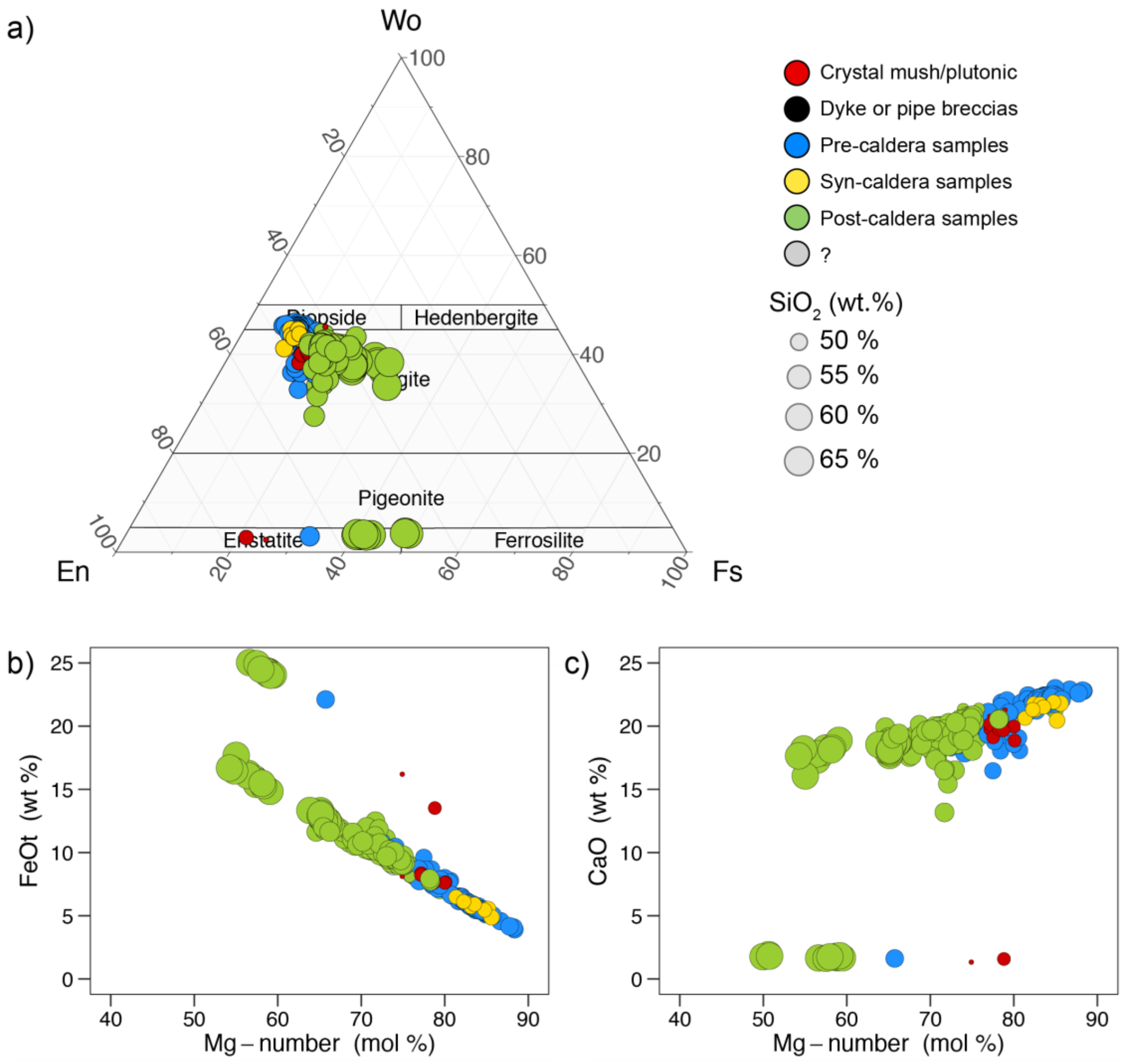

Fig. S5-8: a) Wollastonite (Wo) - Enstatite (En) - Ferrosilite (Fs) ternary diagram for the classification of Ca-Mg-Fe pyroxenes, Morimoto et al. (1989); b) FeO_t vs Mg# in pyroxenes; c) CaO vs Mg# in pyroxenes. SiO_2 symbols refer to the silica content of rock samples containing the analysed pyroxene crystal (see Supplementary Material 1 for details on composition and exact latitude-longitude coordinates of the rock samples). This figure was generated with RStudio Version 1.0.143 (https://www.rstudio.com/) using ggplot2 package Version 2.1.9000 (http://www.ggplot2.org) and ggtern package Version 2.2.1 (http://www.ggtern.com/), plotting systems for R. Final layout of this figure was achieved using Adobe Illustrator CC 2015.3.1 (Copyright © 1987–2016 Adobe Systems Incorporated and its licensors).

Feldspar

Plagioclase is extremely variable from anorthite to oligoclase (Fig. S5-9), for instance, in basalts the widest range goes from An_{32} to An_{92}. Most of the plagioclase phenocrysts in the basalts show calcium-rich compositions (mainly bytownites and anorthites), whereas microphenocrysts and microcrysts are mostly classified as labradorite and minor andesine (An_{44-70}). Plagioclases in minor basalts are also more sodic (An_{32-64}) and are mainly classified as andesine (and minor labradorite). The anorthite contents decrease in the most evolved samples (trachytes) with Pl compositions ranging from An_{13} to An_{40}. The groundmass microlites are oligoclase (An > 20%) with the highest K_2O contents and the lowest CaO/Al_2O_3 ratios. The intermediate compositions (trachybasalts, basaltic trachyandesites, and trachyandesites) present Pl with anorthite contents ranging from 35 to 75%. Those Pl crystals belonging to the post-caldera volcanic period show the widest range in composition (An_{12-90}). This is in accordance with the chemistry of Ol and pPx and with the large range of whole-rock compositions representing the recent volcanism on Deception Island.

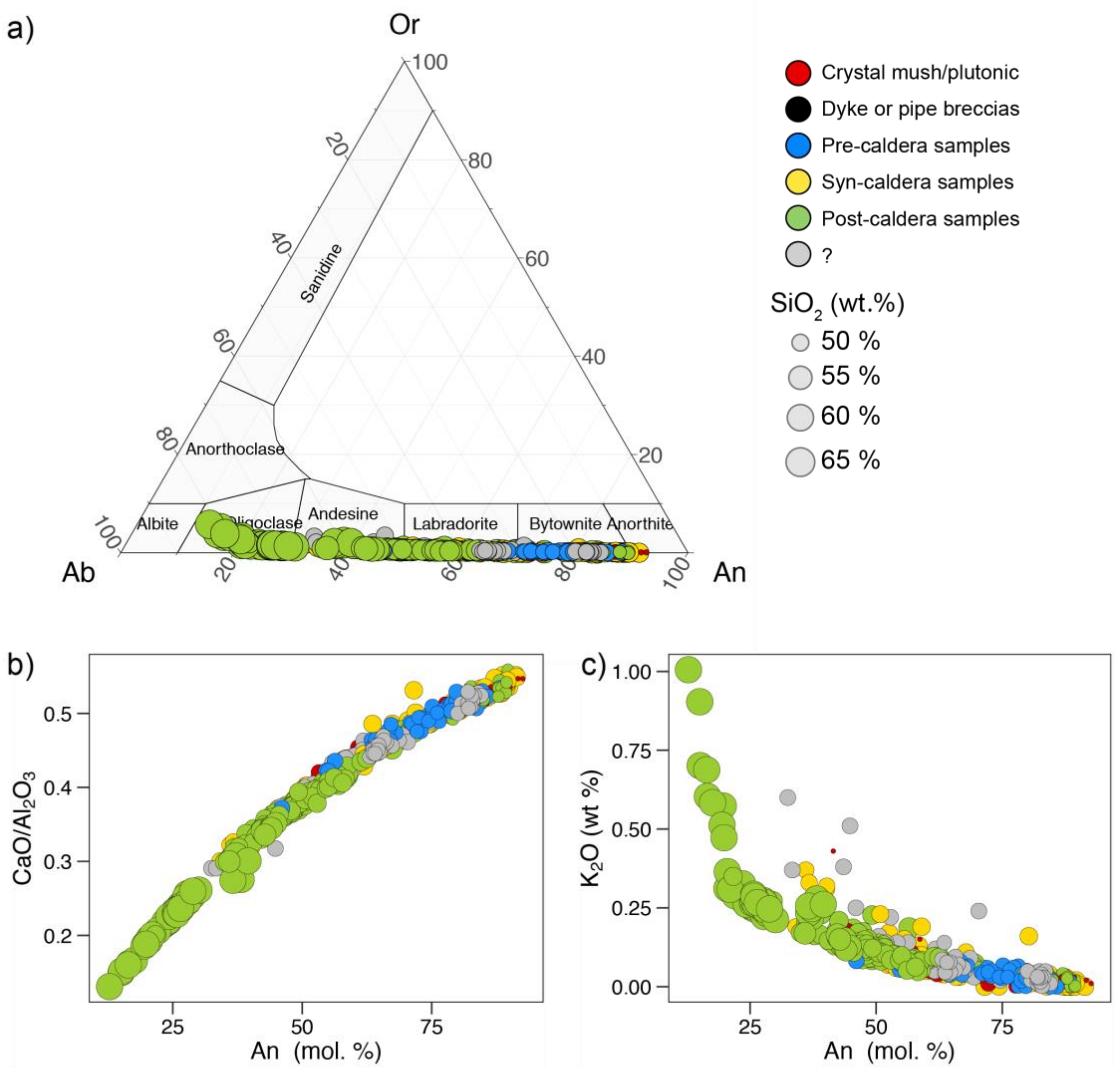

Fig. S5-9:a) Orthoclase (Or) - Albite (Ab) - Anorthite (An) ternary diagram for the classification of feldspars[20]*. CaO/Al_2O_3 ratio vs. An content in plagioclase; c) K_2O vs. An content in plagioclase. SiO_2 symbols refer to the silica content of rock samples containing the analysed plagioclase crystal (see Supplementary Material 1 for details on composition and exact latitude-longitude coordinates of the rock samples). This figure was generated with RStudio Version 1.0.143 (https://www.rstudio.com/) using ggplot2 package Version 2.1.9000 (http://www.ggplot2.org) and ggtern package Version 2.2.1 (http://www.ggtern.com/), plotting systems for R. Final layout of this figure was achieved using Adobe Illustrator CC 2015.3.1 (Copyright © 1987–2016 Adobe Systems Incorporated and its licensors).*

Fe-Ti oxides

They are mainly titano-magnetites and ilmenites (Fig. S5-10; see also Weaver et al.[18], and Aparicio et al.[5]). Ilmenites are homogeneous in composition with TiO_2 contents ranging from 46 to 49 wt.%, FeO from 44 to 47 wt.%, MgO from 1.3 to 4.49 wt.%, and MnO from 0.5 to 1.1 wt.%. Titanomagnetites show FeO_t contents of 68-76 wt.%, TiO_2 from 13 to 24 wt.%, Al_2O_3 from 0.5 to 3.2 wt.% and MgO from 0.5 to 4.9 wt.%.

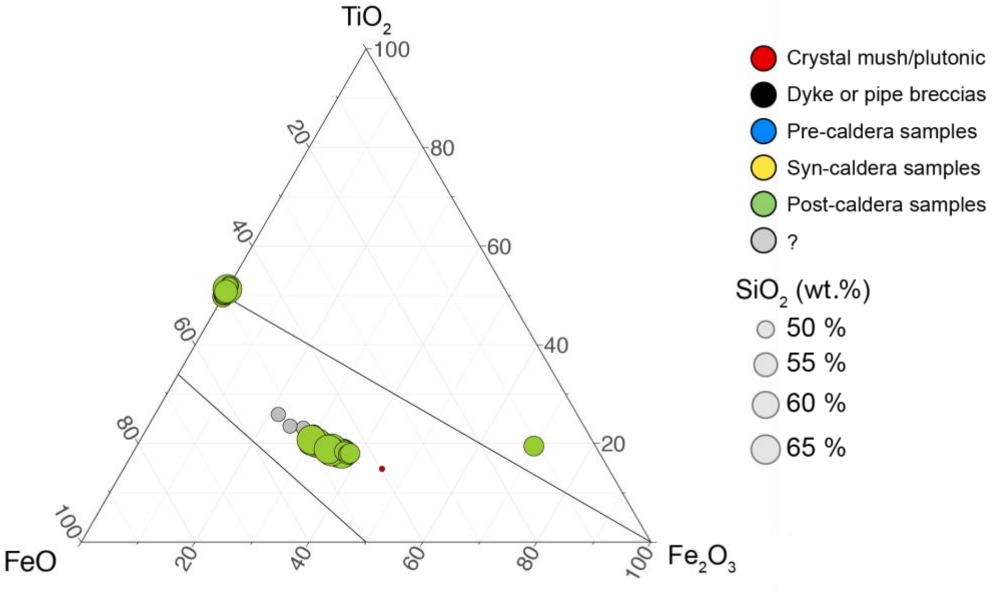

Fig. S5-10: a) Ternary classification diagram for Fe-Ti oxides[21]. SiO_2 symbols refer to the silica content of rock samples containing the analysed Fe-Ti oxide crystal. Ilmenites are very homogeneous (TiO_2 46-49%), while titamagnetites show more compositional variation (see Supplementary Material 1 for details on composition and exact latitude-longitude coordinates of the rock samples). This figure was generated with RStudio Version 1.0.143 (https://www.rstudio.com/) using ggplot2 package Version 2.1.9000 (http://www.ggplot2.org) and ggtern package Version 2.2.1 (http://www.ggtern.com/), plotting systems for R. Final layout of this figure was achieved using Adobe Illustrator CC 2015.3.1 (Copyright © 1987–2016 Adobe Systems Incorporated and its licensors).

Glasses

Glass compositions mainly range from basalts to trachyandesites with minor trachytes and rhyolites (Fig. S5-11). Glasses show a negative correlation of MgO and CaO with SiO_2, as well as a minor positive correlation of K_2O and Na_2O, with depletion at the silica-rich end of the trend ($SiO_2 > 65$ wt.% for Na_2O and $SiO_2 > 70$ wt.% for K_2O). Contents of TiO_2, FeO, Al_2O_3, and MnO show a notable chemical dispersion (Fig. S5-12a, b).

Some samples (e.g., DI-37) are homogeneous in glass composition, whereas different glass populations are identified in most (e.g., DI-29b) (Fig. S5-12c, d). Both glass and bulk-rock compositions (EMP and XRF, respectively) show similar chemical trends (Supplementary Material 1).

Prevalent glass populations in each sample are certainly related to juvenile melts, whereas less represented compositions may be associated either with recycled pyroclasts or magma mingling. For instance, syn-caldera samples show a large variety of glass compositions, with two dominant populations. The most abundant corresponds to glasses with compositions similar to samples within the main cluster group. Most of these samples are poorly crystalline scoriaceous pyroclasts whose whole rock and glass compositions are similar. The less abundant group lies in the main trend of the pre- and post-caldera samples. These glasses are found in bulk ignimbrite samples, where main cluster-like glasses dominate, and whose whole rock compositions are outside the main cluster, in an apparent mixing line between the two dominant glass populations (e.g., DI-29). The average sample compositions could be due to both the incorporation into the ignimbrite deposit of lithic clasts (abundantly found in syn-caldera ignimbrite deposits, and partly represented by minor glass populations), and/or to magma mingling. The minor dominant glass population could represent a deeper, hotter magma, which was fed into the main syn-caldera reservoir and triggered the caldera-forming eruption (Supplementary Material 8).

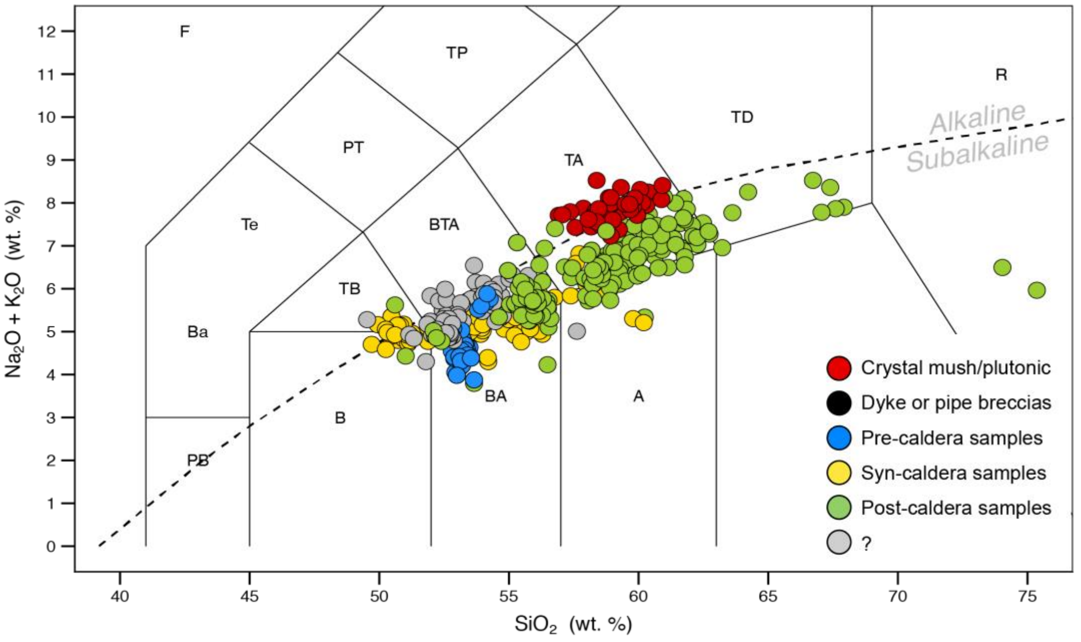

Fig. S5-11: Total Alkali vs. Silica diagram (TAS)[14] for the glasses analyzed in this work (see Supplementary Material 2 for details on composition and exact latitude-longitude coordinates of the rock samples). Major elements normalized to 100% (anhydrous) with Fe distributed from FeO to Fe_2O_3 following Middlemost[15]. Grey dashed line discriminates between the alkaline-subalkaline fields[16]. This figure was generated with RStudio Version 1.0.143 (https://www.rstudio.com/) using ggplot2 package Version 2.1.9000 (http://www.ggplot2.org), a plotting system for R. Final layout of this figure was achieved using Adobe Illustrator CC 2015.3.1 (Copyright © 1987–2016 Adobe Systems Incorporated and its licensors).

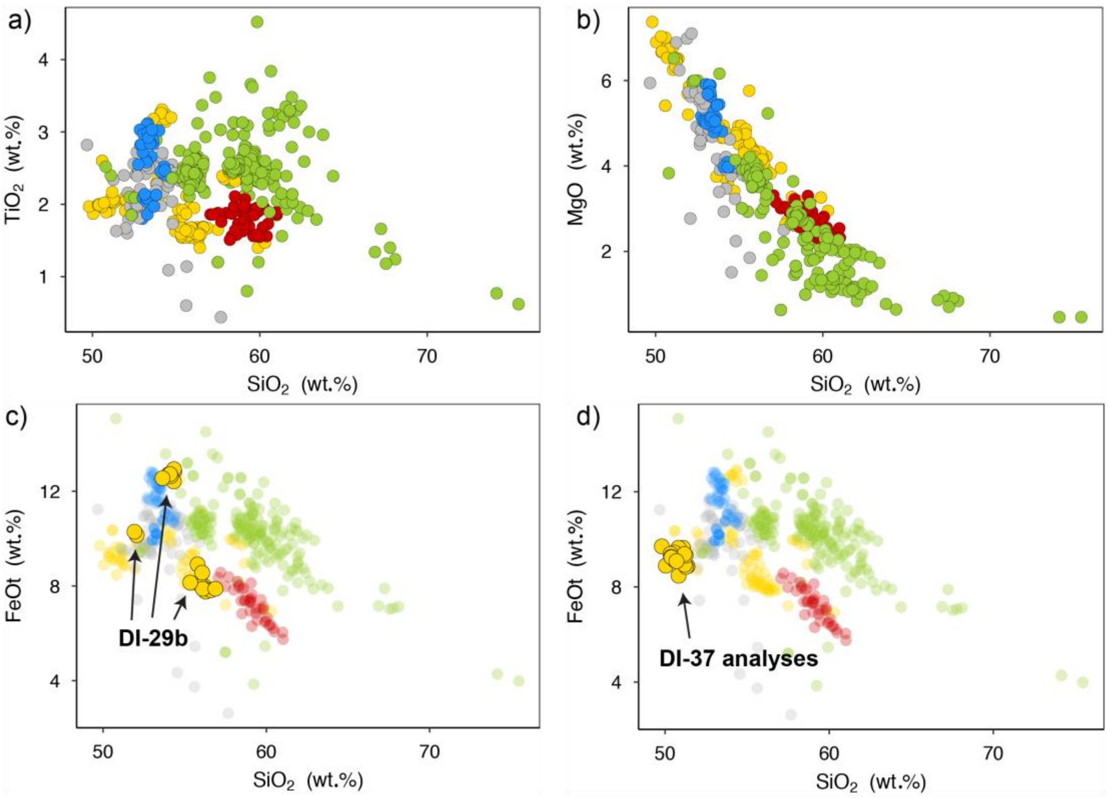

Fig. S5-12: Major elements vs. SiO_2 *content Harker Diagrams for the glasses analysed in this work. In c) heterogeneous glass in DI- 29b is detected by the presence of three glass populations, whereas in d) glass homogeneity is evidenced in sample DI-37 (see Supplementary Material 2 for details on composition and exact latitude-longitude coordinates of the rock samples). Major elements normalized to 100% (anhydrous) with Fe as FeOt This figure was generated with RStudio Version 1.0.143 (https://www.rstudio.com/) using ggplot2 package Version 2.1.9000 (http://www.ggplot2.org), a plotting system for R. Final layout of this figure was achieved using Adobe Illustrator CC 2015.3.1 (Copyright © 1987–2016 Adobe Systems Incorporated and its licensors).*

Isotopic signal of Deception Island samples

Sr isotopic ratios

Sr isotopic ratios were measured on twelve selected samples (Supplementary Material 1) using an IsotopX Phoenix Thermal Ionization Mass Spectrometer (TIMS) at the Centro de Geocronología y Geoquímica Isotópica, Universidad Complutense de Madrid, Spain). Bulk rock powders (ca. 0.1 g) were dissolved with high-purity HF + HNO3 + HCl mixtures. Sr was separated using standard column chromatographic methods with ion-exchange resins. Samples were loaded in Re filaments together with 1µl of H_3PO_4 1M and 2µl of Ta_2O_5. The average $^{87}Sr/^{86}Sr$ value and reproducibility for the isotopic standard NBS 987 during the analytical session was 0.710245 ± 0.00001 (2σ, n=7).

The Sr isotope ratios of the analysed Deception Island's samples range from 0.703396 to 0.703764 (Fig. S5-14). The highest Sr isotope ratio corresponds to a pre-caldera sample (DI-16) with H_2O contents ca. 5 wt.%, which could be indicative of some alteration. Results obtained agree with those reported in previous works for Deception Island [4-9,18,22] (Supplementary Material 2) and are in the range of the Sr isotope ratios reported for the Brandsfield Basin (0.702662 to 0.704288)[23,24]. Sr ratios at Deception Island, which are typical of mantle values, indicate that crustal assimilation was not significant. In fact, there is no correlation between the Sr isotope ratios and the magma composition that could indicate magma-crust interaction. On the other hand, there are no significant isotopic variations throughout time and thus, the pre-, syn- and post-caldera periods are not isotopically distinguishable (Fig. S5-14).

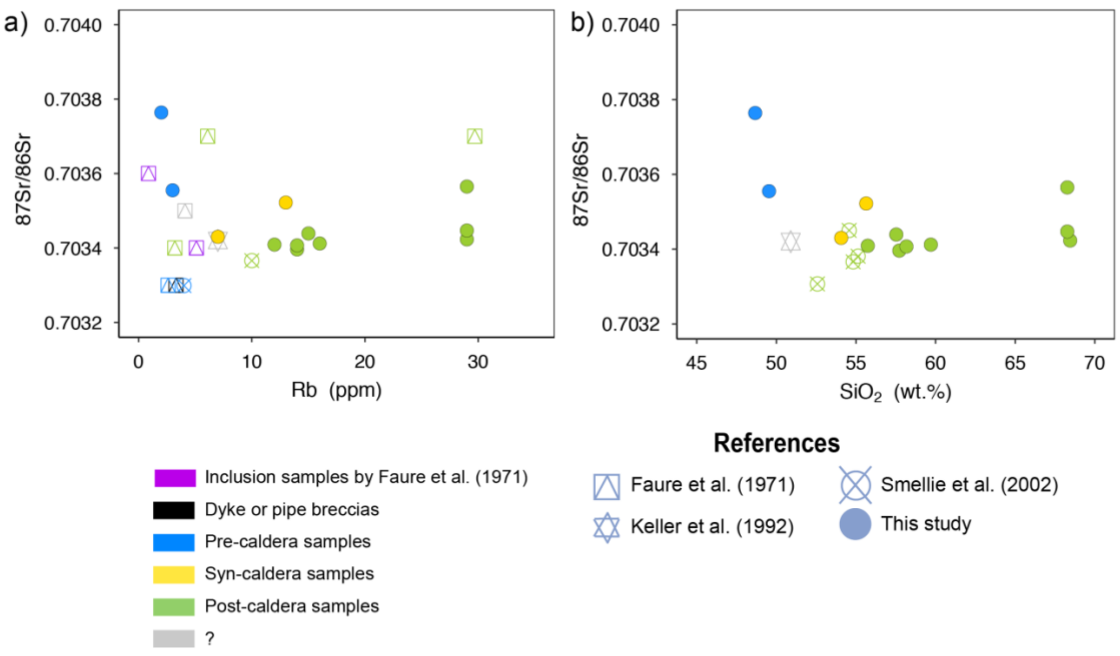

Fig. S5-14: $^{87}Sr/^{86}Sr$ *ratios versus Rb (in ppm) and SiO2 (wt.%). The isotopic ratios are very homogeneous with the exception of a pre-caldera sample that could be related to some alteration. This figure was generated with RStudio Version 1.0.143 (https://www.rstudio.com/) using ggplot2 package Version 2.1.9000 (http://www.ggplot2.org), a plotting system for R. Final layout of this figure was achieved using Adobe Illustrator CC 2015.3.1 (Copyright © 1987–2016 Adobe Systems Incorporated and its licensors).*

References

1 González-Ferrán, O., Munizaga, F. & Moreno R., H. 1970 eruption at Deception island: distribution and chemical features of ejected materials. *Antarctic Journal of the United States* **6**, 87-89 (1971).

2 González-Ferrán, O. & Katsui, Y. Estudio integral del volcanismo cenozoico superior de las Islas Shetland del Sur, Antartica. *Ser. cient. Inst. antart. chileno* **22**, 123-174 (1971).

3 Baker, P. E. & McReath, I. 1970 Volcanic Eruption at Deception Island. *Nature physical science* **231**, 5-9, doi:10.1038/physci231005a0 (1971).

4 Baker, P. E., McReath, I., Harvey, M. R., Roobol, M. J. & Davies, T. G. The geology of the south Shetland islands: Volcanic evolution of Deception island. *British Antarctic Survey Scientific Reports* **78**, 81 pp. (1975).

5 Aparicio, A., Menegatti, N., Petrinovic, I., Risso, C. & Viramonte, J. G. El volcanismo de Isla Decepción (Península Antártida). *Boletin Geológico y Minero* **108**, 235-258 (1997).

6 Smellie, J. L. *et al.* Geology and geomorphology of Deception Island. *BAS GEOMAP Series, Sheets 6-A and 6-B, 1:25 000*,78pp. with accompanying maps, (British Antarctic Survey, Cambridge, 2002).

7 Kraus, S., Kurbatov, A. & Yates, M. Geochemical signatures of tephras from Quaternary Antarctic Peninsula volcanoes. *Andean Geology* **40**, 1-40, doi:10.5027/andgeoV40n1-a01 (2013).

8 Galé, C. *et al.* Vulcanismo cuaternario de la Isla Decepción (Antártida): una signatura relacionada con la subducción de la Fosa de las Shetland del Sur en el dominio de tras-arco de la Cuenca de Bransfield. *Boletín Geológico y Minero* **125**, 31-52 (2014).

9 Keller, R. A., Fisk, M. R., White, W. M. & Birkenmajer, K. Isotopic and trace element constraints on mixing and melting models of marginal basin volcanism, Bransfield Strait, Antarctica. *Earth and Planetary Science Letters* **111**, 287-303, doi:10.1016/0012-821X(92)90185-X (1992).

10 De Rosa, R., Mazzuoli, R., Omarini, R. H., Ventura, G. & Viramonte, J. A Volcanological Model for the Historical Eruptions at Deception Island (Bransfield Strait, Antarctica). *Terra Antarctica* **2**, 95-101 (1995).

11 Martí, J., Geyer, A. & Aguirre-Diaz, G. Origin and evolution of the Deception Island caldera (South Shetland Islands, Antarctica). *Bulletin of Volcanology* **75**, 1-18, doi:10.1007/s00445-013-0732-3 (2013).

12 Le Maitre, R. W. *et al.* A classification of Igneous Rocks and Glossary terms: Recomendations of the International Union of Geological Sciences Subcommission on the Systematics of Igneous Rocks., (Blackwell Scientific Publications, Oxford, U.K, 1989).

13 Torrecillas, C., Berrocoso, M. & García-García, A. The Multidisciplinary Scientific Information Support System (SIMAC) for Deception Island, in *Antarctica* (eds Dieter Karl Fütterer *et al.*) Ch. 50, 397-402 (Springer Berlin Heidelberg, 2006).

14 Le Bas, M. J., Le Maitre, R. W., Streckeisen, A. & Zanettin, B. A Chemical Classification of Volcanic Rocks Based on the Total Alkali-Silica Diagram. *Journal of Petrology* **27**, 745-750, doi:10.1093/petrology/27.3.745 (1986).

15 Middlemost, E. A. K. Iron oxidation ratios, norms and the classification of volcanic rocks. *Chemical Geology* **77**, 19-26, doi:10.1016/0009-2541(89)90011-9 (1989).

16 Irvine, T. N. & Baragar, W. R. A. A guide to the chemical classification of the common volcanic rocks. *Canadian Journal of Earth Sciences* **8**, 523-548, doi:10.1139/e71-055 (1971).

17 Sun, S. S. & McDonough, W. F. Chemical and isotopic systematics of oceanic basalts; implications for mantle composition and processes, in *Magmatism in the ocean basins* (eds A. D. Saunders & M.J. Norry) 313-435 (Geological Society of London, 1989).

18 Weaver, S. D., Saunders, A. D., Pankhurst, R. J. & Tarney, J. A geochemical study of magmatism associated with the initial stages of back-arc spreading. *Contributions to Mineralogy and Petrology* **68**, 151-169, doi:10.1007/bf00371897 (1979).

19 Morimoto, N. c. Nomenclature of pyroxenes. *The Canadian Mineralogist* **27**, 143-156 (1989).

20 Deer, W. A., Zussman, J. & Howie, R. A. Rock-forming minerals. Vol. 4A, Framework silicates : feldspars. 2nd edn,972 pp., (Geological Society of London Pub. House, Bath, 2001).

21 Chang, L. L. Y., Zussman, J. & Howie, R. A. Rock-forming minerals. Volume 5B, Non-silicates: sulphates, carbonates, phosphates, halides. 2nd edn,383 pp., (Geological Society, London, 1998).

22 Faure, G., Shulz, C. H. & Carwile, R. H. Isotope composition of strontium in volcanic rocks from Deception Island. *Antarctic Journal of the United States* **6**, 197-198 (1971).

23 Keller, R. A., Fisk, M. R., Smellie, J. L., Strelin, J. A. & Lawver, L. A. Geochemistry of back arc basin volcanism in Bransfield Strait, Antarctica: Subducted contributions and along-axis variations. *Journal of Geophysical Research: Solid Earth* **107**, ECV 4-1-ECV 4-17, doi:10.1029/2001JB000444 (2002).

24 Fretzdorff, S. *et al.* Magmatism in the Bransfield Basin: Rifting of the South Shetland Arc? *Journal of Geophysical Research: Solid Earth* **109**, doi:10.1029/2004JB003046 (2004).

Deciphering the evolution of Deception Island's magmatic system

A. Geyer (1), A.M. Álvarez-Valero (2), G. Gisbert (3), M. Aulinas (4), D. Hernández-Barreña (2), A. Lobo (1), J. Marti (1)

(1) Institute of Earth Sciences Jaume Almera, ICTJA, CSIC, Lluis Sole i Sabaris s/n, 08028 Barcelona, Spain

(2) Departamento de Geología, Universidad de Salamanca, 37008 Salamanca, Spain

(3) Instituto de Geociencias, CSIC-UCM, Severo Ochoa 7, 28040 Madrid, Spain

(4) Departament de Mineralogia, Petrologia i Geologia Aplicada. Universitat de Barcelona, Marti Franques s/n, 08028 Barcelona, Spain

SUPPLEMENTARY MATERIAL 6

Deception Island's magmatism within the regional magmatic framework

Data compilation and processing

The geochemical database of Deception Island's rock samples (Supplementary Materials 1 and 2) has been integrated with a selection of available analytical data from South Shetland Islands and Bransfield Rift area obtained from the GEOROC database (Geochemistry of Rocks of the Oceans and Continents, http://georoc.mpch-mainz.gwdg.de/georoc/) and an exhaustive compilation of published research [1-23] (Supplementary Material 3). Analyses were discarded following the same considerations as in Supplementary Material 5, and also when the sample's age was older than 200 Ma (beginning of Phoenix plate subduction beneath Antarctic Peninsula[24]).

In addition, we only selected samples with MgO contents ranging from 5 to 10 wt.% to be plotted in composition-normalized multi-element and Ba/Nb vs. Nb/Zr diagrams. This allows minimizing the effects of fractional crystallization and crystal accumulation on initial trace element contents and ratios, in order to favour the comparisons between magma source compositions and partial melting degrees. MgO < 5 wt.% suggests that magma underwent substantial amounts of fractional crystallization during its evolution, whereas MgO > 10 wt.% may indicate olivine accumulation in the magma[25]. No filtering has been applied for trace element compositions because the effect of crustal contamination in most suites, including Deception Island's (DI), is negligible [11,22,23].

We grouped the analyses as a function of their geographic location and main geochemical features into (Fig. S6-1): 1) South Shetland Islands (except Deception Island); 2) Bransfield Rift alkaline rocks; 3) Bransfield Rift subalkaline rocks with minor subduction influence; 4) Bransfield Rift subalkaline rocks with significant subduction influence; and 5) Deception Island.

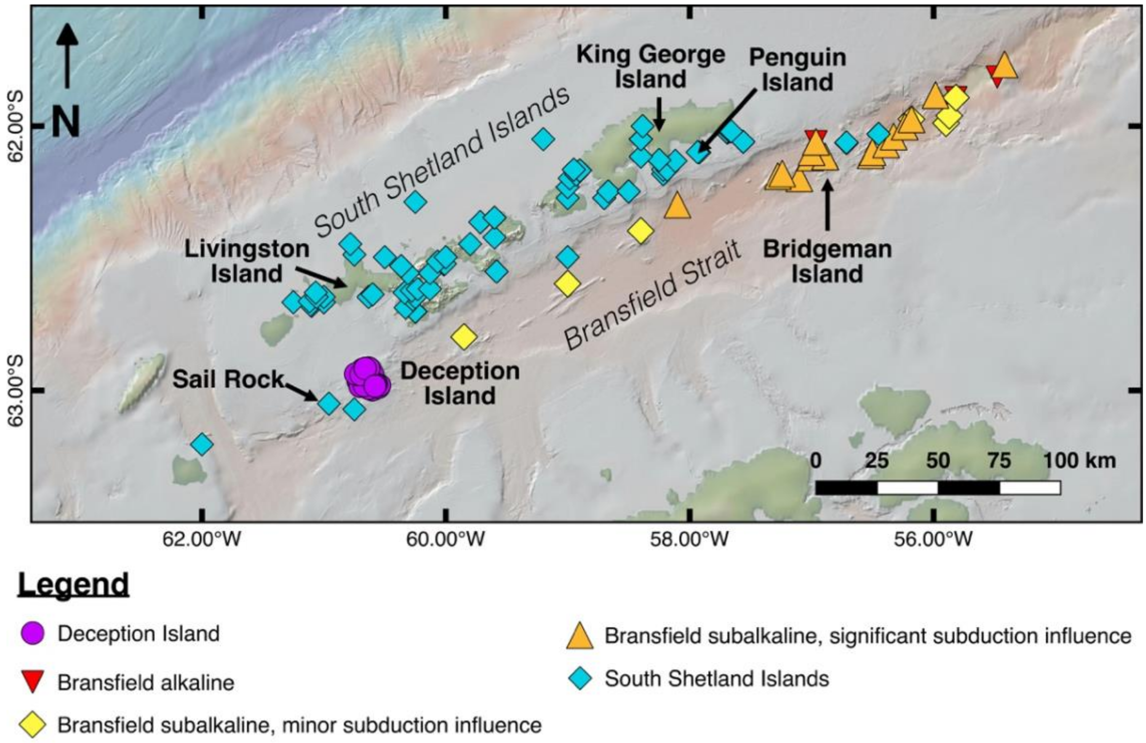

Fig. S6-1: Location map of the rock samples considered in this study (see Supplementary Materials 1-3 for exact latitude-longitude coordinates of the rock samples). This figure was generated with QGIS software version 2.18 Las Palmas (available at: [www.qgis.org)](www.qgis.org) and GeoMapApp v. 3.6.6 (http://www.geomapapp.org). The base map used corresponds to the Global Multi-Resolution Topography (GMRT, http://www.marine-geo.org/portals/gmrt/) Synthesis[26].

Regional magmatic setting

The South Shetland Islands, Bransfield Rift and Larsen Rift-James Ross Island areas have been the active volcanic regions in the northern Antarctic Peninsula during Cenozoic times[22,23]. The complex geodynamics, mostly controlled by the Phoenix plate subduction under the Antarctic Peninsula, conditioned their evolution and magma composition[21-23] (Table S6-1).

The formation of the South Shetland Islands magmatic arc is related to a westward shift in arc magmatism location within the Antarctic Peninsula during Cretaceous times. The main building stage lasted from ca. 135 to 24 Ma ago[23], yet the volcanic activity in the area is still on-going[22]. In the Late Cenozoic, the subduction velocity decreased (convergence rates from 2.5 to 7.5 mm/yr for the last 2 Ma[27]), and the extensional tectonics, mostly related to slab roll back[12,24], promoted the Bransfield and Larsen Rift formations within a back-arc setting[9,11,12,16,28]. Development of the Bransfield Rift from the Pliocene onwards (ca. 4 Ma) opened the Bransfield Strait, separating the South Shetland Islands arc from the Antarctic Peninsula[24,29,30].

In the Bransfield Rift area both alkaline and subalkaline rocks have been described (Figure S6-2). Alkaline rocks occur NE of King George Island [19], with trace element contents indicating formation through low partial melting (high Nb/Zr) of a depleted mantle source similar to N-MORB with no subduction influence (absence of Large Ion Lithophile Elements, LILE, enrichment relative to High Field Strength Elements, HFSE, low Ba/Nb, Figs. S6-3, S6-5, S6-7). The genesis of the alkaline samples was related to upwelling and decompression melting of a fresh asthenospheric mantle without significant contribution from a subduction-modified mantle[19]. Subalkaline rocks have a wider compositional range than the alkaline ones (Fig. S6-2). Samples with minor subduction influence were dredged from seamounts along the bottom of Bransfield Rift SW of King George Island and NE of Bridgeman Island. These are mostly basalts and basaltic andesites represented in the arc-tholeiites field of Peccerillo and Taylor[31] (Figs. S6-2, S6-4). These magmas may have formed by higher degrees of partial melting, compared to the alkaline ones (lower Nb/Zr), of a depleted mantle source with minor subduction influence (slight

LILE enrichment relative to HFSE; lower than on South Shetland Island rocks, (e.g., lower Ba/Nb ratios, Figs. S6-3, S6-5, S6-7)[11,23]. On the other hand, subalkaline rocks with significant subduction influence (mostly sampled NE of King George Island) range in composition from similar to rocks in the previously described subalkaline group to compositions with higher subduction component influence, similar to rocks from the South Shetland Islands (e.g., Ba/Nb ratios from 20 to > 100, Fig. S6-3). Therefore, the samples of the Bransfield area suggest a heterogeneous mantle source with variable influence from subduction processes

At the South Shetland Islands, typical subduction-controlled subalkaline basaltic to rhyolitic arc magmas erupted during the active arc magmatism stage. Trace element and isotope compositions indicate a depleted mantle source modified by contributions from the subducting slab (both from sediments and altered oceanic crust; e.g., marked LILE enrichment relative to HFSE, high (> 20) Ba/Nb ratios, Figs. S6-3 and S6-5)[16,22,23,32]. Low Yb concentrations have been interpreted to indicate a garnet peridotite mantle source, thus requiring relatively deep melting conditions (> 80 km)[22].

Quaternary volcanism in the South Shetlands archipelago, strongly connected to rifting and back-arc basin formation[12,22], occurs at Livingston and King George Islands, but is mostly concentrated at Penguin, Bridgeman, and Deception Islands[12,22,33]. Quaternary magmas of the South Shetlands islands, except Deception (considered separately due to its own geochemical signal), are mostly subalkaline basaltic and basaltic andesitic (Fig. S6-2) with trace element compositions suggesting a mantle source similar to that of the active arc period (Figs. S6-5 to S6-7). Some magmas from Livingston, King George, and Penguin Islands present higher alkalinity than previous active arc magmas[2,6,13,22], which is given by higher contents in Na_2O and K_2O. These magmas have the highest Nb/Zr and lowest Ba/Nb ratios within the entire compositional range of the South Shetland Islands (Fig. S6-3). This may reveal a lower degree of partial melting and contribution from the subduction component.

Area	Magmatism	Age	Location	Composition	Subduction influence on magma source	Partial melting degree	Magma source and genesis
**Bransfield Rift**	Alkaline	< 4 Ma	NE of King George Island	Alkaline. Basalts, trachybasalts, basanites	none (low Ba/Nb, < 10)	low (high Nb/Zr, > 0.1)	Low degree of partial melting of a depleted mantle source (similar to that of N-MORB). Decompression melting of a fresh asthenospheric mantle without significant contribution from a subduction-modified mantle
	Subalkaline with minor subduction influence	< 4 Ma	Seamounts along the bottom of Bransfield Rift SW of King George Island and NE of Bridgeman Island	Subalkaline. Mostly basalts and basaltic andesites	minor (low Ba/Nb, < 20)	high (low Nb/Zr, < 0.1)	High degrees of partial melting of a depleted mantle source with minor subduction influence
	Subalkaline with significant subduction influence	< 4 Ma	Mostly NE of King George Island	Subalkaline. Basalts to rhyolites	major (high Ba/Nb, 20 to > 100)	high (low Nb/Zr, < 0.1)	High degrees of partial melting of a depleted mantle source with significant variable subduction influence
**South Shetland Islands**	Main active arc period	135–24 Ma	Major South Shetland Islands except Deception	Subalkaline. Basalts to rhyolites	major (high Ba/Nb, 20 to >100)	high (low Nb/Zr, < 0.1)	High degrees of partial melting of a depleted mantle source with significant subduction contribution (both from sediments and altered oceanic crust)
	Quaternary magmatism outside Deception Island	< 2 Ma	Penguin, Bridgeman, Livingston and King George Islands	Subalkaline. Basalts to basaltic andesites. Occurrence of some magmas with higher alkalinity than active arc ones	major (high Ba/Nb, 20 to > 100) Lower for higher alkalinity magmas	high (low Nb/Zr, < 0.1). Lower for higher alkalinity magmas	Same mechanism as for active arc magmas. Occurrence of some magmas with higher alkalinity. These formed from lower partial melting degrees and contribution of subduction component
**Deception Island**		< 2 Ma	Deception Island	Subalkaline. Basalts to trachytes and rhyolites	minor (low-medium Ba/Nb, < 30)	high (low Nb/Zr, < 0.1), but slightly lower than for subalkaline SW Bransfield Rift magmas	High degree of partial melting of a depleted mantle source with minor subduction influence similar to that of subalkaline Bransfield Rift magmas SW of King George Islands.

Table S6-1: Summary of the main features of the Bransfield Rift, South Shetland Islands and Deception Island magmatisms.

Deception Island's vs.
South Shetlands and Bransfield Rift magmatisms

Even though magmas in Deception Island are contemporaneous with the Quaternary South Shetland Islands and Bransfield Rift magmatisms, they are different from a geochemical perspective[23,34,35]. Deception Island magmas follow a distinctive a lkalinity-increasing differentiation trend at the upper end of the subalkaline field in the TAS diagram (Fig. S6-2) produced by higher Na_2O contents (Figs. S6-4 and S6-8). Unusually high Na_2O contents at Deception Island magmas mark a compositional deviation from the normal active arc andesite-rhyolite associations in the circum-Pacific areas[34], and Na/K ratios that are only similar to ocean ridge basalts[35]. Indeed, Deception Island magmas fall close to the composition of subalkaline Bransfield Rift samples with the lowest subduction influence in Figure S6-9, outside the compositional field of active volcanic arcs worldwide, which includes the rest of the magmatic suites in the South Shetland archipelago.

The trace element compositions of Deception Island's rocks are similar to those of the subalkaline Bransfield Rift samples with the lowest influence of subduction component (low Ba/Nb, Fig. S6-3), indicating a magma mantle source of close composition. Therefore, magmas at Deception Island were formed in a different mantle source with respect to the other South Shetland Islands magmas, one with less subduction influence.

Deception Island magmas have slightly higher trace element abundances at the same MgO content, and higher enrichment of the more incompatible elements compared to the less incompatible ones, i.e., higher La/Lu ratios, than Bransfield Rift rocks. We suggest that a lower partial melting degree for the magmas of Deception Island with respect to the magmas of Bransfield Rift may account for the higher alkalinity and incompatible trace element enrichment (which produces higher Nb/Zr ratios in Fig. S6-3). The lower partial melting degree might be related to the marginal location of Deception Island relative to both the rift and the low subduction component (i.e., water content) in the mantle source.

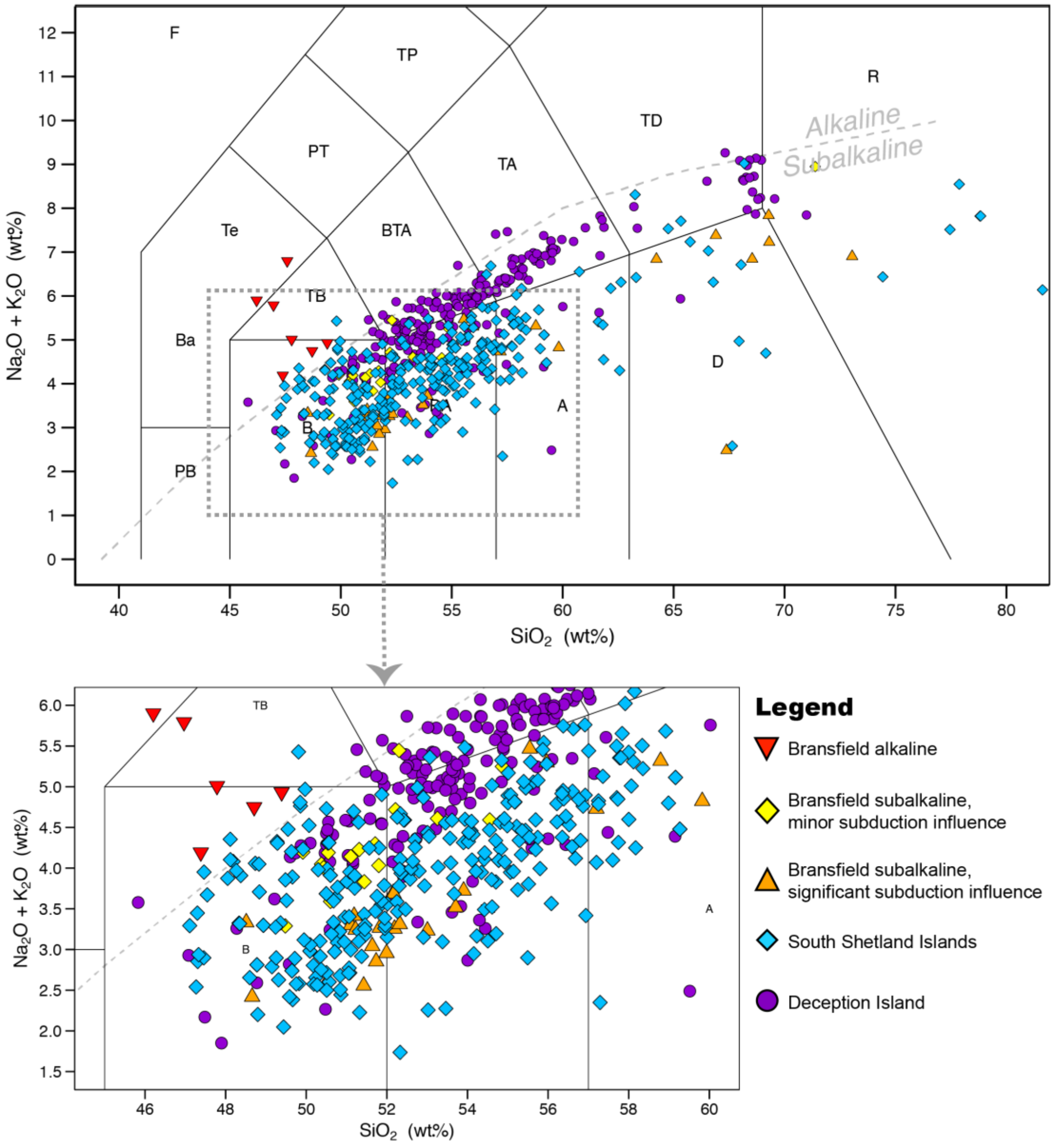

Fig. S6-2: Total Alkali vs. Silica diagram (TAS)[36] *for the rock samples considered in this work (see Supplementary Materials 1-3 for details on composition and exact latitude-longitude coordinates of the rock samples). Major elements normalized to 100% (anhydrous) with Fe distributed from FeO to Fe_2O_3 following Middlemost*[37]*. Black dashed line separates alkaline and subalkaline fields*[38]*. This figure was generated with RStudio Version 1.0.143 (https://www.rstudio.com/) using ggplot2 package Version 2.1.9000 (http://www.ggplot2.org), a plotting system for R. Final layout of this figure was achieved using Adobe Illustrator CC 2015.3.1 (Copyright © 1987–2016 Adobe Systems Incorporated and its licensors).*

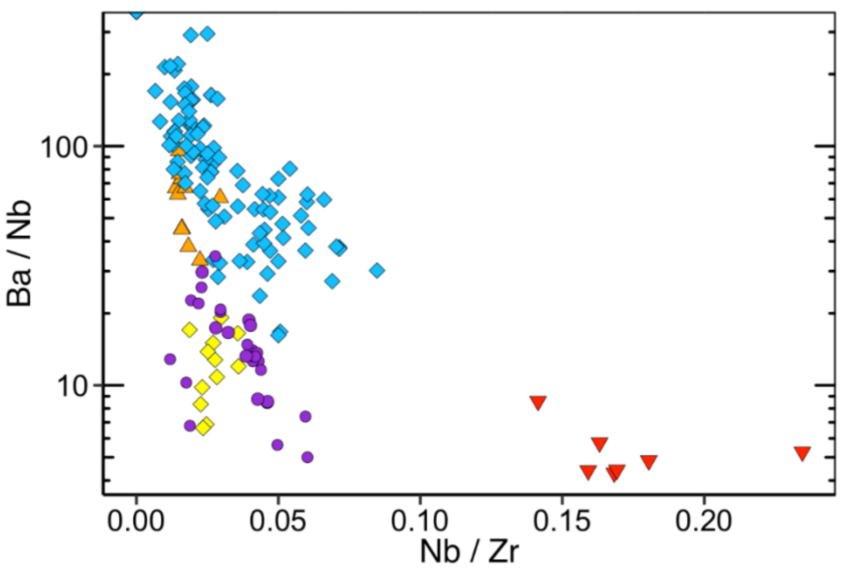

Fig. S6-3: Ba/Nb vs. Nb/Zr diagram for samples with 5-10 wt.% MgO (see Supplementary Materials 1-3 for details on composition and exact latitude-longitude coordinates of the rock samples). This figure was generated with RStudio Version 1.0.143 (https://www.rstudio.com/) using ggplot2 package Version 2.1.9000 (http://www.ggplot2.org), a plotting system for R. Legend as per Fig. S6-2.

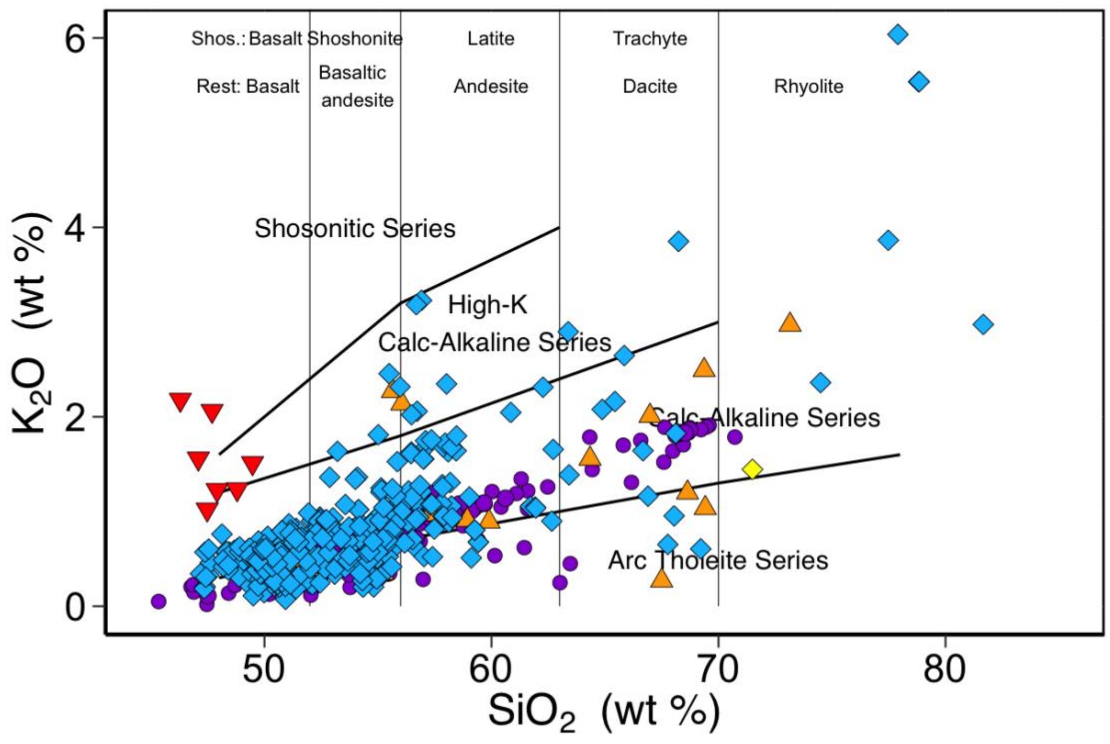

Fig. S6-4: K_2O vs. SiO_2 diagram (wt.% normalized on an H_20- and CO_2-free basis; see Supplementary Materials 1-3 for details on composition and exact latitude-longitude coordinates of the rock samples). Rock type fields as in Peccerillo and Taylor[31]. This figure was generated with RStudio Version 1.0.143 (https://www.rstudio.com/) using ggplot2 package Version 2.1.9000 (http://www.ggplot2.org), a plotting system for R. Legend as per Fig. S6-2.

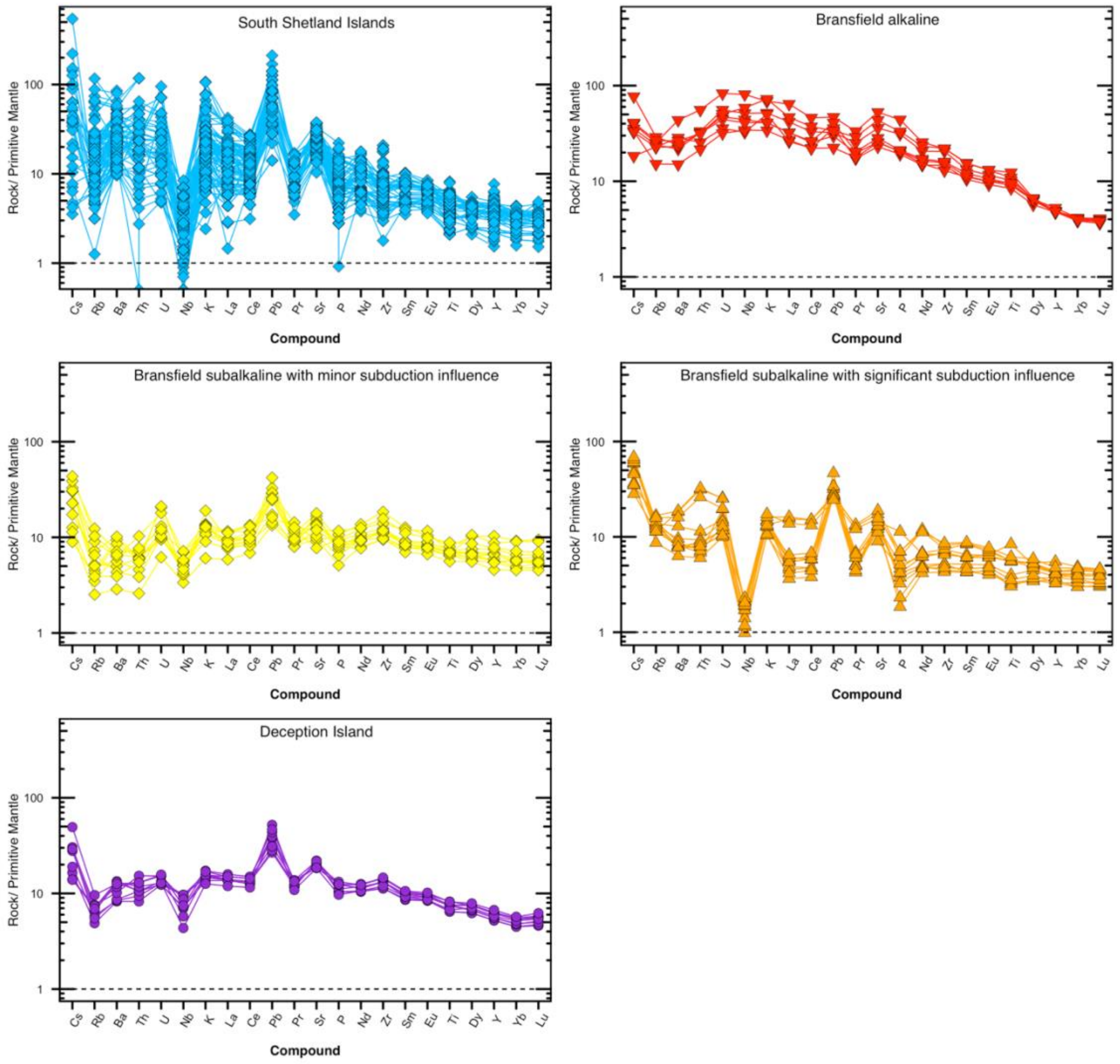

Fig. S6-5: Whole rock composition of samples with 5-10 wt.% MgO normalized to primitive mantle composition[39] (see Supplementary Materials 1-3 for details on composition and exact latitude-longitude coordinates of the rock samples). This figure was generated with RStudio Version 1.0.143 (https://www.rstudio.com/) using ggplot2 package Version 2.1.9000 (http://www.ggplot2.org), a plotting system for R. Legend as per Fig. S6-2.

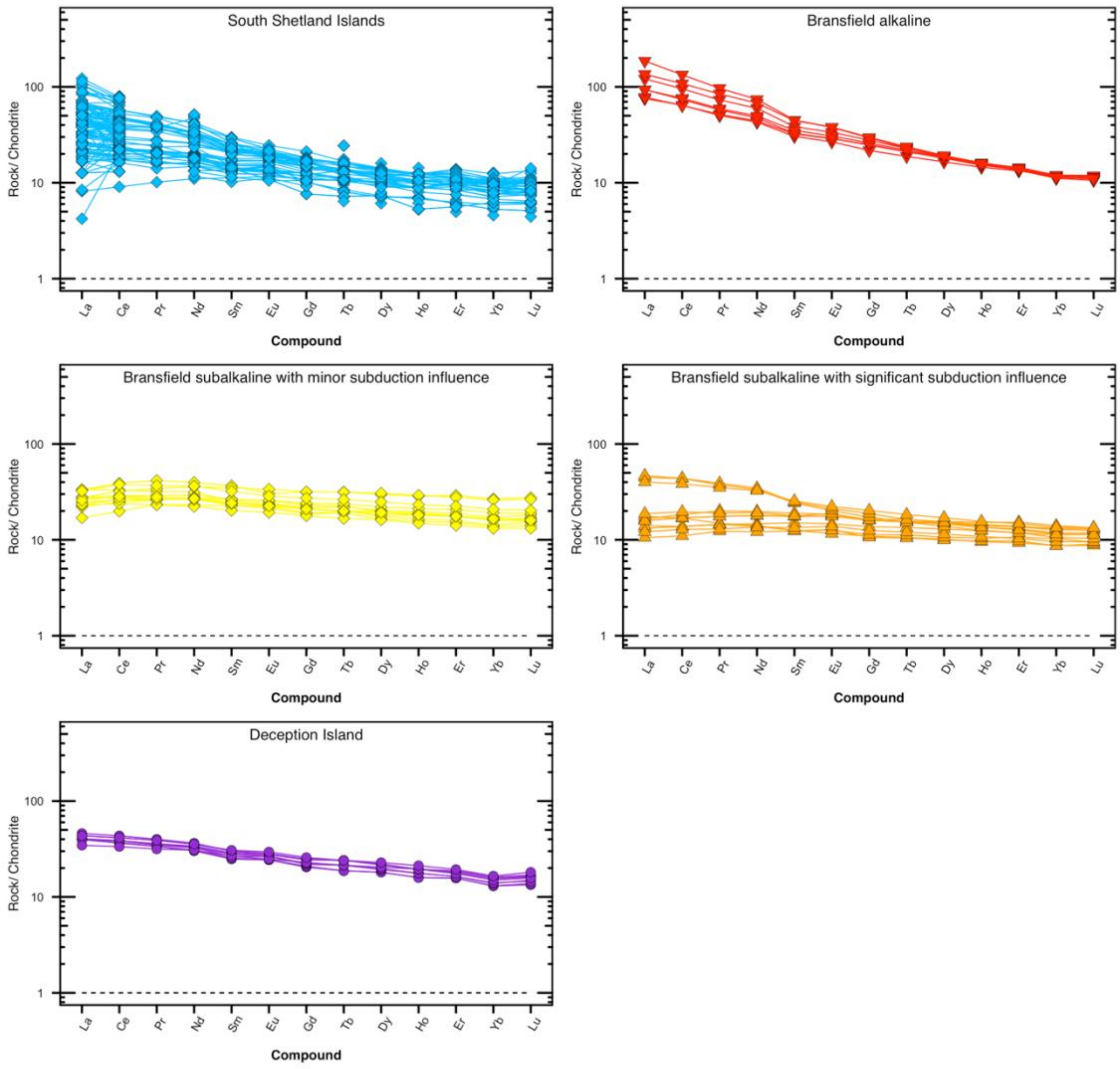

Fig. S6-6: Whole rock composition of samples with 5-10 wt.% MgO normalized to chondrite composition[39] *(see Supplementary Materials 1-3 for details on composition and exact latitude-longitude coordinates of the rock samples). This figure was generated with RStudio Version 1.0.143 (https://www.rstudio.com/) using ggplot2 package Version 2.1.9000 (http://www.ggplot2.org), a plotting system for R. Legend as per Fig. S6-2.*

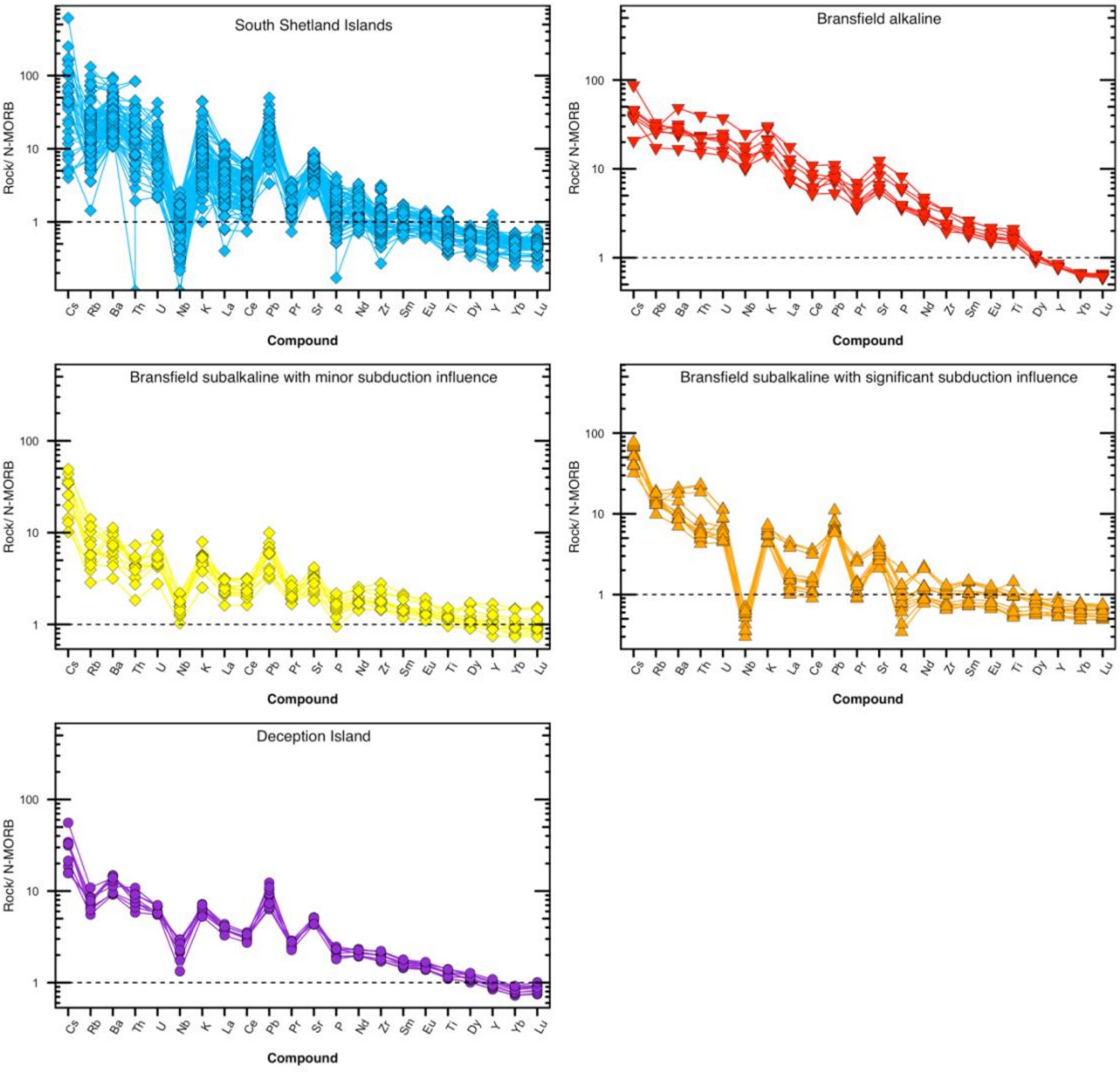

Fig. S6-7: Whole rock composition of samples with 5-10 wt.% MgO normalized to N-MORB composition[39] *(see Supplementary Materials 1-3 for details on composition and exact latitude-longitude coordinates of the rock samples). Rock type fields as in Peccerillo and Taylor* [31]*. This figure was generated with RStudio Version 1.0.143 (https://www.rstudio.com/) using ggplot2 package Version 2.1.9000 (http://www.ggplot2.org), a plotting system for R. Legend as per Fig. S6-2.*

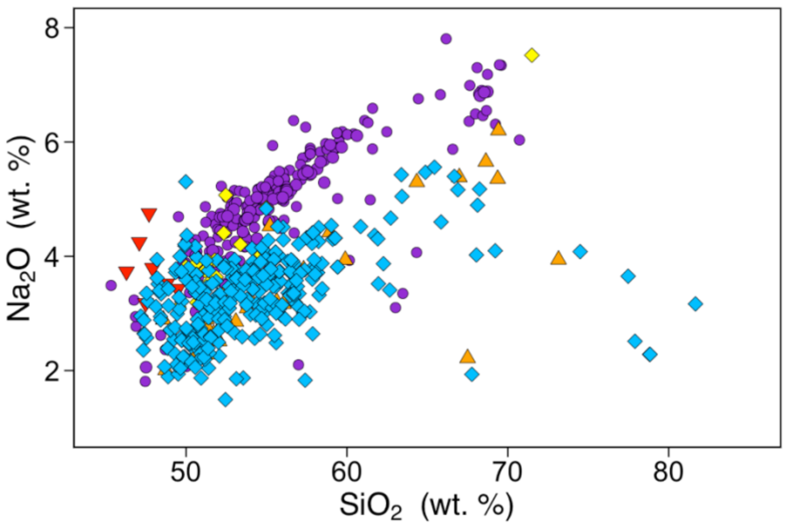

Fig. S6-8: Na_2O vs. SiO_2 diagram (wt.% normalized on an H_20- and CO_2-free basis; see Supplementary Materials 1-3 for details on composition and exact latitude-longitude coordinates of the rock samples). This figure was generated with RStudio Version 1.0.143 (https://www.rstudio.com/) using ggplot2 package Version 2.1.9000 (http://www.ggplot2.org), a plotting system for R. Legend as per Fig. S6-2.

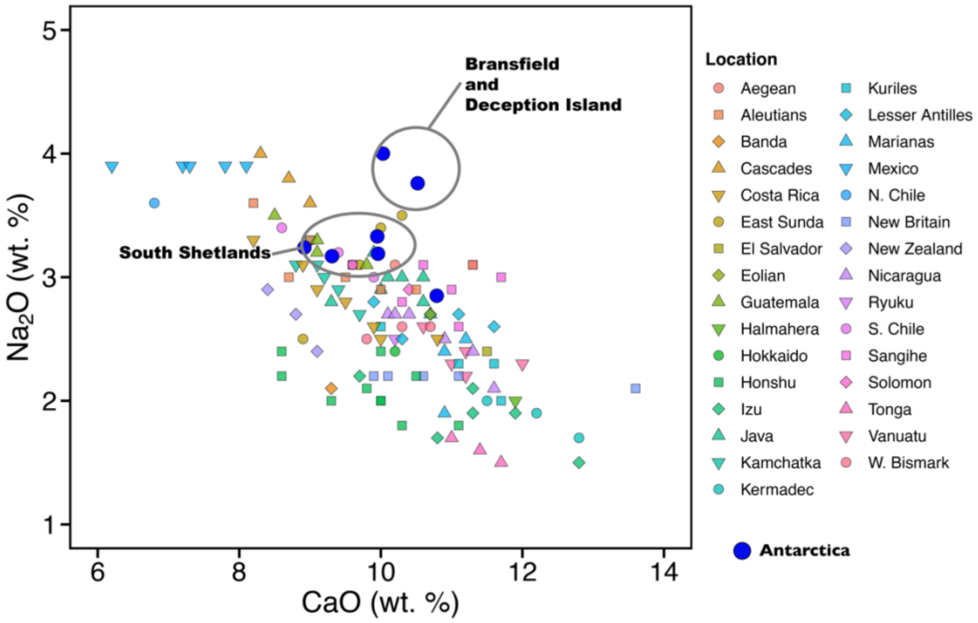

Fig. S6-9. Average compositions of volcanic centres and volcanic arcs normalized to 6 wt.% MgO[25] in a Na_2O vs. CaO diagram. Note that South Shetland Islands suites are within the compositional field of arc magmas whereas Deception Island suite is not. DI is next to Bransfield Rift rocks with minor subduction influence. This figure was generated with RStudio Version 1.0.143 (https://www.rstudio.com/) using ggplot2 package Version 2.1.9000 (http://www.ggplot2.org), a plotting system for R. Final layout of this figure was achieved using Adobe Illustrator CC 2015.3.1 (Copyright © 1987–2016 Adobe Systems Incorporated and its licensors).

References

1 Tarney, J., Saunders, A. D. & Weaver, S. D. Geochemistry of volcanic rocks from the island arcs and marginal basins of the Scotia Arc region, in *Island arcs, deep sea trenches and back-arc basins* (eds M. Talwani & W. C. III Pitman) 367-377 (AGU 1977).

2 Weaver, S. D., Saunders, A. D., Pankhurst, R. J. & Tarney, J. A geochemical study of magmatism associated with the initial stages of back-arc spreading. *Contributions to Mineralogy and Petrology* **68**, 151-169, doi:10.1007/bf00371897 (1979).

3 Birkenmajer, K., Narebski, W., Skupinski, A. & Bakun-Czubarow, N. Geochemistry and origin of the Tertiary island-arc calc-alkaline volcanic suite at Admiralty Bay, King George Island (South Shetland Islands, Antarctica). *Stud. Geol. Polonica* **72**, 7-57 (1981).

4 Moyes, A. B. & Hamer, R. D. Contrasting origins and implications of garnet in rocks of the Antarctic Peninsula, in *Antarctic Earth Science 4th Intern. Symp.* (eds R. L. Oliver, P. R. James, & J. B. Jago) 358-362 (Cambridge Univ. Press 1983).

5 Smellie, J. L. A geochemical overview of subduction-related igneous activity in the South Shetland Islands, Lesser Antarctica. , in *Antarctic Earth Science* (ed R. L. Oliver) 352-356 (Austral. Acad. Sci. Canberra and Cambridge Univ. Press. , 1983).

6 Smellie, J. L., Pankhurst, R. J., Thomson, M. R. A. & Davies, R. E. S. The geology of the south Shetland Islands: VI.stratigraphy,geochemistry and evolution. *British Antarctic Survey Scientific reports* **87**, 2-83 (1984).

7 Birkenmajer, K., Narebski, W., Bakun-Czubarow, N. & Kalmus, M. Geochemistry and petrogenesis of calc-alkaline Mesozoic volcanics and Andean plutons of Admiralty Bay, King George Island (South Shetland Islands, Antarctica). *Stud. Geol. Polonica* **82**, 7-51 (1985).

8 LeMasurier, W. E. *et al.* Volcanoes of the Antarctic Plate and Southern Oceans Vol. 48 *Antarctic Research Series* (eds W.E. LeMasurier & J.W. Thomson),487 pp., 1990).

9 Hole, M. J. Geochemical evolution of Pliocene-Recent post-subduction alkalic basalts from Seal Nunataks, Antarctic Peninsula. *Journal of Volcanology and Geothermal Research* **40**, 149-167, doi:10.1016/0377-0273(90)90118-Y (1990).

10 Birkenmajer, K., Francalanci, L. & Peccerillo, A. Petrological and geochemical constraints on the genesis of Mesozoic–Cenozoic magmatism of King George Island, South Shetland Islands, Antarctica. *Antarctic Science* **3**, 293-308, doi:10.1017/S0954102091000354 (1991).

11 Keller, R. A., Fisk, M. R., White, W. M. & Birkenmajer, K. Isotopic and trace element constraints on mixing and melting models of marginal basin volcanism, Bransfield Strait, Antarctica. *Earth and Planetary Science Letters* **111**, 287-303, doi:10.1016/0012-821X(92)90185-X (1992).

12 Hole, M. J., Saunders, A. D., Rogers, G. & Sykes, M. A. The relationship between alkaline magmatism, lithospheric extension and slab window formation along

continental destructive plate margins. *Geological Society, London, Special Publications* **81**, 265-285, doi:10.1144/gsl.sp.1994.081.01.15 (1994).

13 Smellie, J. L., Pallàs, R., Sàbat, F. & Zheng, X. Age and correlation of volcanism in central Livingston Island, South Shetland Islands: K-Ar and geochemical constraints. *Journal of South American Earth Sciences* **9**, 265-272, doi:10.1016/0895-9811(96)00012-0 (1996).

14 Willan, R. C. R. & Kelley, S. P. Mafic dike swarms in the South Shetland Islands volcanic arc: Unravelling multiepisodic magmatism related to subduction and continental rifting. *Journal of Geophysical Research: Solid Earth* **104**, 23051-23068, doi:10.1029/1999JB900180 (1999).

15 De Almeida, D. P. M. *et al.* An extrusive-plutonic event at Hardy Point and its vicinity, Greenwich Island, Antarctica. *Rev. Bras. Geocienc* **30**, 12-16 (2000).

16 Keller, R. A., Fisk, M. R., Smellie, J. L., Strelin, J. A. & Lawver, L. A. Geochemistry of back arc basin volcanism in Bransfield Strait, Antarctica: Subducted contributions and along-axis variations. *Journal of Geophysical Research: Solid Earth* **107**, ECV 4-1-ECV 4-17, doi:10.1029/2001JB000444 (2002).

17 De Almeida, D. P. M. *et al.* An igneous event at the Fildes Peninsula (King George Island) and around Fort Point (Greenwich Island), South Shetland Islands, Antarctica. *Rev. Bras. Geocienc* **33**, 339-348 (2003).

18 Demant, A., Touron, S., Lapierre, H. & Bosch, D. Cretaceous arc volcanism of Byers Peninsula, Livingston Island, Antarctica : new petrological, geochemical and isotope data. *Bulletin de la Société Géologique de France* **175**, 131-145, doi:10.2113/175.2.131 (2004).

19 Fretzdorff, S. *et al.* Magmatism in the Bransfield Basin: Rifting of the South Shetland Arc? *Journal of Geophysical Research: Solid Earth* **109**, doi:10.1029/2004JB003046 (2004).

20 Machado, A. *et al.* Modeling of subduction components in the Genesis of the Meso-Cenozoic igneous rocks from the South Shetland Arc, Antarctica. *Lithos* **82**, 435-453, doi:10.1016/j.lithos.2004.09.026 (2005).

21 Košler, J. *et al.* Combined Sr, Nd, Pb and Li isotope geochemistry of alkaline lavas from northern James Ross Island (Antarctic Peninsula) and implications for back-arc magma formation. *Chemical Geology* **258**, 207-218, doi:10.1016/j.chemgeo.2008.10.006 (2009).

22 Haase, K. M., Beier, C., Fretzdorff, S., Smellie, J. L. & Garbe-Schönberg, D. Magmatic evolution of the South Shetland Islands, Antarctica, and implications for continental crust formation. *Contributions to Mineralogy and Petrology* **163**, 1103-1119, doi:10.1007/s00410-012-0719-7 (2012).

23 Kraus, S., Kurbatov, A. & Yates, M. Geochemical signatures of tephras from Quaternary Antarctic Peninsula volcanoes. *Andean Geology* **40**, 1-40, doi:10.5027/andgeoV40n1-a01 (2013).

24 Barker, P. F. The Ceozoic subduction histrory of the Pacific margin of the Antarctic Peninsula: ridge crest-trench interactions. *J. geol. Soc. London* **139**, 787-801 (1982).

25 Plank, T. & Langmuir, C. H. An evaluation of the global variations in the major element chemistry of arc basalts. *Earth and Planetary Science Letters* **90**, 349-370, doi:10.1016/0012-821X(88)90135-5 (1988).

26 Ryan, W. B. F. *et al.* Global Multi-Resolution Topography synthesis. *Geochemistry, Geophysics, Geosystems* **10**, n/a-n/a, doi:10.1029/2008GC002332 (2009).

27 Henriet, J. P., Meissner, R., Miller, H. & The Grape, T. Active margin processes along the Antarctic Peninsula. *Tectonophysics* **201**, 229-253, doi:10.1016/0040-1951(92)90235-X (1992).

28 González-Ferrán, O. The Larsen Rift: an active extension fracture in West Antarctica, in *Antarctic Earth Science* (eds R. Oliver, P. James, & J. Jago) 344-346 (Cambridge University Press, 1983).

29 Larter, R. D. & Barker, P. F. Effects of ridge crest-trench interaction on Antarctic-Phoenix Spreading: Forces on a young subducting plate. *Journal of Geophysical Research: Solid Earth* **96**, 19583-19607, doi:10.1029/91JB02053 (1991).

30 Barker, D. H. N., Christeson, G. L., Austin, J. A. J. & Dalziel, I. W. D. Backarc basin evolution and cordilleran orogenesis: Insights from new ocean-bottom seismograph refraction profiling in Bransfield Strait, Antarctica *Geology* **31**, 107-110, doi:10.1130/0091-7613(2003)031<0107:BBEACO>2.0.CO;2 (2003).

31 Peccerillo, A. & Taylor, S. R. Geochemistry of eocene calc-alkaline volcanic rocks from the Kastamonu area, Northern Turkey. *Contributions to Mineralogy and Petrology* **58**, 63-81, doi:10.1007/bf00384745 (1976).

32 Machado, A. *et al.* Geochemistry constraints of Mesozoic–Cenozoic calc-alkaline magmatism in the South Shetland arc, Antarctica. *Journal of South American Earth Sciences* **18**, 407-425, doi:10.1016/j.jsames.2004.11.011 (2005).

33 Birkenmajer, K., Soliani, E. & Kawashita, K. Reliability of potassium-argon dating of Cretaceous-Tertiary island-arc volcanic suites of King George Island, South Shetland Islands (West Antarctica). *Zentralblatt fur Geologie und Palaöntologie* **1**, 127-140 (1990).

34 Hawkes, D. D. The geology of the South Shetland Islands: II. The geology and petrology of Deception Island. *Falkland Islands Dependencies Survey Scientific Reports* **27**, 43 (1961).

35 Smellie, J. L. *et al.* Geology and geomorphology of Deception Island. *BAS GEOMAP Series, Sheets 6-A and 6-B, 1:25 000*,78pp. with accompanying maps, (British Antarctic Survey, Cambridge, 2002).

36 Le Bas, M. J., Le Maitre, R. W., Streckeisen, A. & Zanettin, B. A Chemical Classification of Volcanic Rocks Based on the Total Alkali-Silica Diagram. *Journal of Petrology* **27**, 745-750, doi:10.1093/petrology/27.3.745 (1986).

37 Middlemost, E. A. K. Iron oxidation ratios, norms and the classification of volcanic rocks. *Chemical Geology* **77**, 19-26, doi:10.1016/0009-2541(89)90011-9 (1989).

38 Irvine, T. N. & Baragar, W. R. A. A guide to the chemical classification of the common volcanic rocks. *Canadian Journal of Earth Sciences* **8**, 523-548, doi:10.1139/e71-055 (1971).

39 Sun, S. S. & McDonough, W. F. Chemical and isotopic systematics of oceanic basalts; implications for mantle composition and processes, in *Magmatism in the ocean basins* (eds A. D. Saunders & M.J. Norry) 313-435 (Geological Society of London, 1989).

Deciphering the evolution of Deception Island's magmatic system

A. Geyer (1), A.M. Álvarez-Valero (2), G. Gisbert (3), M. Aulinas (4), D. Hernández-Barreña (2), A. Lobo (1), J. Marti (1)

(1) Institute of Earth Sciences Jaume Almera, ICTJA, CSIC, Lluis Sole i Sabaris s/n, 08028 Barcelona, Spain

(2) Departamento de Geología, Universidad de Salamanca, 37008 Salamanca, Spain

(3) Instituto de Geociencias CSIC-UCM, Severo Ochoa 7, 28040 Madrid, Spain

(4) Departament de Mineralogia, Petrologia i Geologia Aplicada. Universitat de Barcelona, Marti Franques s/n, 08028 Barcelona, Spain

SUPPLEMENTARY MATERIAL 7

Geophysical imaging of Deception Island's internal structure and magmatic system

Crustal structure beneath Deception Island

Geophysical observations in previous literature have allowed imaging the crustal structure beneath the Bransfield Strait and Deception Island (DI) (e.g., [1,2]). Crustal thickness in the region may range from about 10 km for the oceanic crust of the Drake plate to about 25 km for the South Shetland Islands shelf, and 30–33 km for the South Shetland Islands crustal block[1-3]. Below DI, the Moho discontinuity has been estimated to be 15–20 km deep[4].

According to existing seismic models, crustal structure beneath the Bransfield Strait is characterized by an uppermost layer corresponding to young unconsolidated or poorly consolidated sediments (ν_p = 1.9–2.5km/s)[1-3] underlain by a second sedimentary layer comprised by older and better consolidated sediments and lavas (~ 4.0–5.7 km/s)[1-3](Figs. S7-1). Along the Bransfield Strait, these two sedimentary layers (up to 2.5 km thick) cover an approximately 10-km-wide anomalous body (ν_p =6.8 km/s, 2- to 7-km depth range), which runs parallel to the rift spreading axis and is assumed to be the active Bransfield Rift[1]. Away from the rift axis, the sedimentary layers are underlain by a complex basement with diverse crustal complexes having different compositions and physical properties[1-3]. The crustal structure beneath DI has been widely investigated applying gravity and magnetic studies [5,6], demonstrating the existence of two types of crust (i.e., continental and incipient oceanic) separated by an ENE–WSW fault dipping toward the south (Fig. S7-1). This crustal fault is also well constrained by seismic reflection and refraction profiles[2,7].

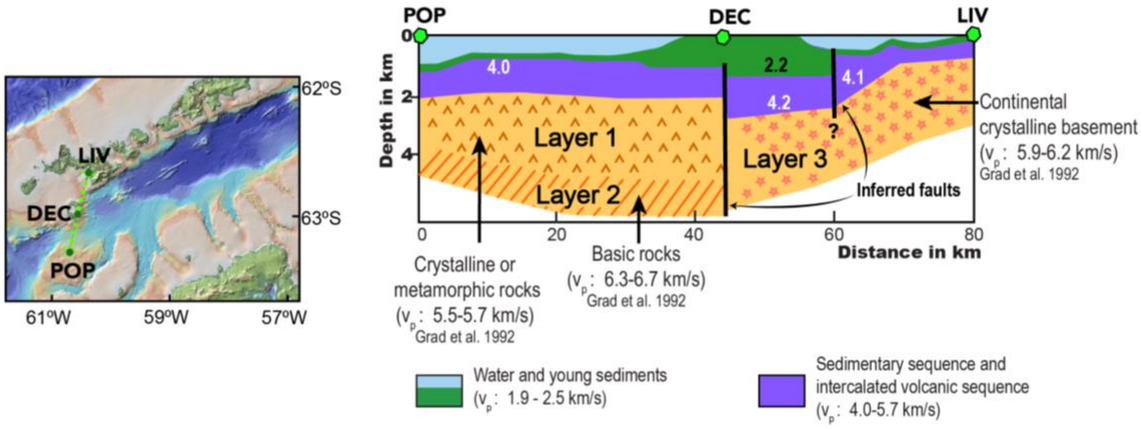

Fig. S7-1: Seismic model of the upper crust structure along a profile of the Bransfield Strait-Deception Island-Livingston based on the results by Grad et al. [1-3]. This figure was generated with QGIS software version 2.18 Las Palmas (available at: www.qgis.org), QGIS2threejs plugin, and GeoMapApp v 3.6.6 (http://www.geomapapp.org). The basemap used corresponds to the Global Multi-Resolution Topography (GMRT, http://www.marine-geo.org/portals/gmrt/) Synthesis[8]. Final layout was obtained with Adobe Illustrator CC 2015.3.1 (Copyright © 1987–2016 Adobe Systems Incorporated and its licensors).

Deception Island's internal structure and magmatic system

Numerous multidisciplinary studies carried out on and around DI have allowed imaging its internal structure and magmatic system, as well as monitoring its volcanic activity. Two conceptual models of DI's internal structure have been proposed to explain the existing geophysical observations. Both models coincide in their interpretation of the shallower structure (0–2 km depth beneath Port Foster Bay), but show some discrepancies at deeper crustal levels (> 2 km depth beneath Port Foster Bay)[9]. We briefly summarize the most relevant aspects concerning DI's internal structure and geophysical imaging of the island's magmatic system. For more details, the reader is referred to Prudencio et al. [9] and references therein.

Shallow structure: 0–2 km depth beneath Port Foster

Results from ambient seismic noise analysis indicate that the first ~ 400 m of materials beneath Port Foster (v_s : ~ 0.2 – 1.1 km/s), would correspond to volcanic deposits and sediments from the different post-caldera eruptions that have occurred within and around the bay[10] (Fig. S7-2). Below ~ 400 m, it is possible to identify a ~ 1 km-thick layer v_s : ~ 1.3 – 2.8 km/s) interpreted as ignimbrites (Outer Coast Tuff Formation) deposited during the caldera-forming event[10,11](Fig. S7-2). In parallel, between 1 and 2 km depth, isotopes and noble gas data from fumarolic and bubbling gases and hot spring waters allow inferring the existence of a heated hydrothermal system with different temperatures [12].

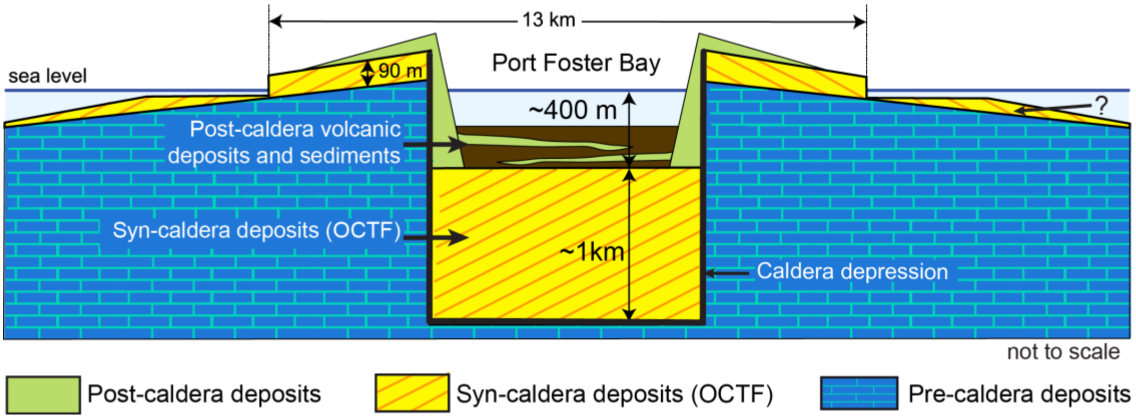

Fig. S7-2: Sketch of DI's shallow internal structure based on S-wave velocity values estimated by Luzón et al. [10]. Only well-constrained layers combined with a volcanostratigraphic interpretation of the deposits are shown (modified from Martí et al. [11]) OCTF: Outer Coast Tuff Formation. This figure was created using Adobe Illustrator CC 2015.3.1 (Copyright © 1987–2016 Adobe Systems Incorporated and its licensors).

Deep structure: > 2 km depth beneath Port Foster

Between 2 km and up to 6–10 km depth beneath Port Foster, observed anomalies of physical properties have been commonly interpreted as evidence of the existence of a certain amount of partially melted rock/material variable in volume (e.g., [5,9,13-15]). As an example, the 3D resistivity model obtained by Pedrera et al. [14] after an extensive magnetotelluric survey in 2008, reveals an ENE–WSW elongated conductor east of Whalers Bay ranging from 2 to 10 km depth (Fig. S7-3), interpreted as a combination of partial melt and hot fluids[14]. Thus, the ENE–WSW crustal fault revealed by magnetic and gravity studies[5,6] is assumed to facilitate the emplacement of melt, which apparently accumulates in the hanging wall of the fault[14] (Fig. S7-4). Secondary fractures with different orientations could connect the melt and hydrothermal fluids with the surface.

In addition, strong seismic velocity variations in the first kilometres of the crust are attributed to the presence of crustal magmatic systems with either partially melted regions and frozen intrusive bodies, or sediment thickness variations and geothermal systems[9,10,13,15] . Indeed, seismic tomography results show the presence of a low P-wave velocity sector beneath the caldera floor interpreted as a shallow magma chamber[13,15] (Fig. S7-5). In addition, a very low density anomaly

in both magnetic and gravity anomaly maps of DI have been interpreted as a partially melted intrusive body with its top at 1.7 km depth[5]. Some authors state that at these same depths, fractured rocks and the existence of a geothermal system may also be, at least partially, responsible for the observed geophysical signals (e.g., [11]) (Fig. S7-6). Deep feeding structures may simply heat the upper crustal systems, where meteoric waters both penetrate and circulate producing the high-attenuation anomaly in the centre of the caldera. Thus, the large hydrothermal system developed in the interior of the caldera depression may be responsible for most of the present-day observations up to 6 km depth[9].

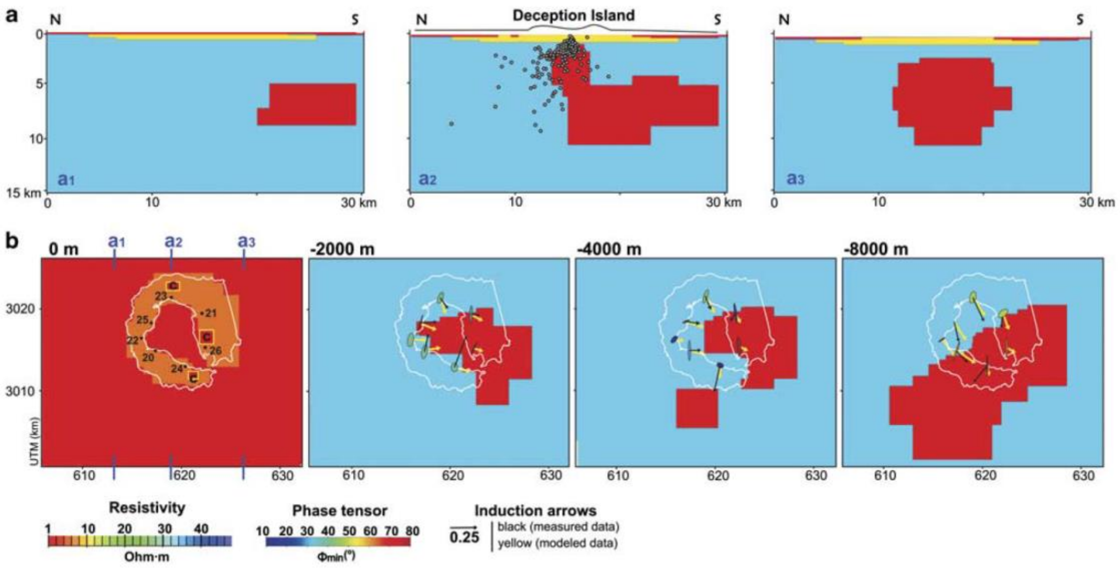

Fig. S7-3: North-south cross sections (a) and horizontal slices (b) of the 3-D resistivity model proposed by Pedrera et al.[14] The location of earthquake hypocenters recorded during the 1992 and 1999 seismic crises [16] is also indicated (cross section a2). The induction arrows and phase tensor ellipses at periods 0.5, 10, and 100 s approximately correspond with the depth of the slices, pointing towards the conductor. Colors of phase tensor ellipses correspond to the length of the minor axis. The induction arrows derived from the model are plotted (Figure obtained from Pedrera et al.[14]).

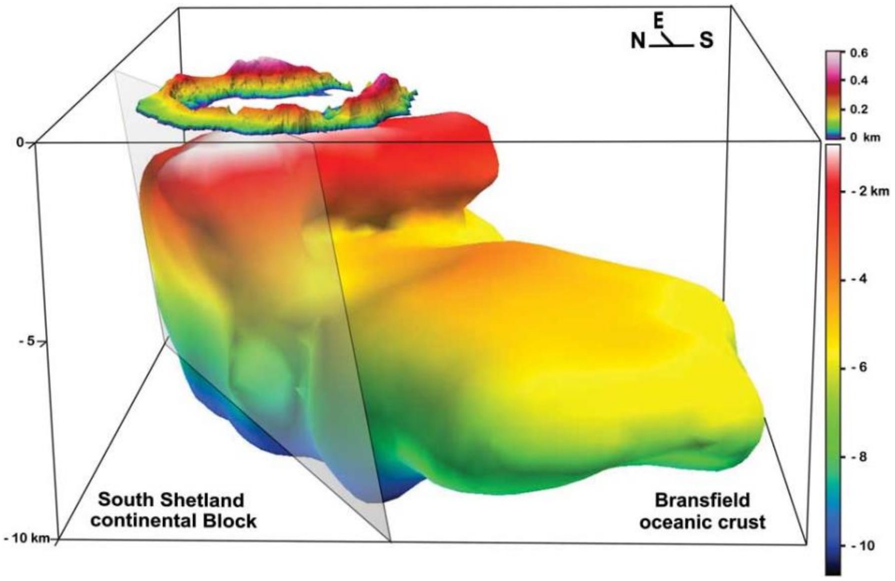

Fig. S7-4: Sketch of Deception Island showing the location of the conductive body that suggests the emplacement of melt driven by an ENE–WSW oriented and SSE dipping regional normal fault according to the results by Pedrera et al. [14] (Figure obtained from Pedrera et al. [14])

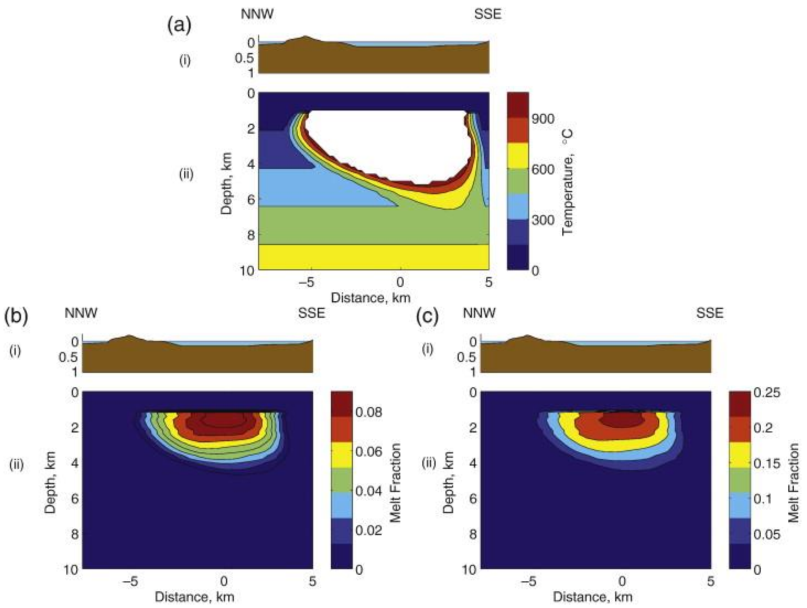

Fig. S7-5: Results for melt calculations provided by Ben-Zvi et al. [13] *(a) Temperatures calculated from the seismic velocity anomaly in the vicinity of the island along the NNW–SSE profile (see Ben-Zvi et al.* [13] *for more details on the methodology and results). White shading indicates a region where the temperatures exceed the solidus temperature of 1050 °C. Note that temperature anomalies are not calculated for the upper 1.2 km since this corresponds to the sediment basin. Melt fractions calculated from the excess negative velocity anomalies and a melt pocket aspect ratio of 0.05 (b) and of 1.0 (c). (Figure from Ben-Zvi et al.* [13]*)*

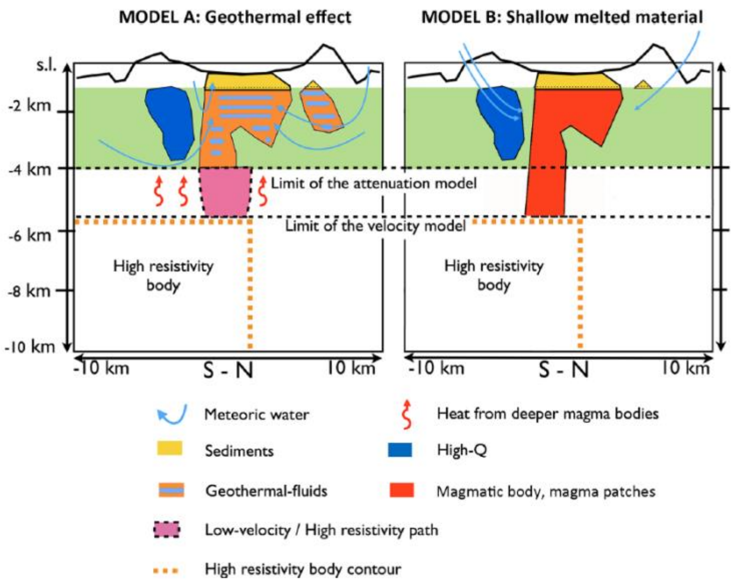

Fig. S7-6: Schematic interpretation proposed by Prudencio et al. [9] *based on their own P-wave attenuation model (up to 4 km depth), and also considering the existing 3D velocity* [15] *(up to 5.5 km depth) and resistivity* [14] *models (Figure from Prudencio et al.* [9]*) .*

References

1 Grad, M., Guterch, A. & Janik, T. Seismic structure of the lithosphere across the zone of subducted Drake plate under the Antarctic plate, West Antarctica. *Geophysical Journal International* **115**, 586-600, doi:10.1111/j.1365-246X.1993.tb01209.x (1993).

2 Grad, M., Shiobara, H., Janik, T., Guterch, A. & Shimamura, H. Crustal model of the Bransfield Rift, West Antarctica, from detailed OBS refraction experiments. *Geophysical Journal International* **130**, 506-518, doi:10.1111/j.1365-246X.1997.tb05665.x (1997).

3 Grad, M., Guterch, A. & Sroda, P. Upper crustal structure of Deception Island area, Bransfield Strait, West Antarctica. *Antarctic Science* **4**, 469-476, doi:10.1017/S0954102092000683 (1992).

4 Christeson, G. L., Barker, D. H. N., Austin, J. A. & Dalziel, I. W. D. Deep crustal structure of Bransfield Strait: Initiation of a back arc basin by rift reactivation and propagation. *Journal of Geophysical Research: Solid Earth* **108**, doi:doi:10.1029/2003JB002468 (2003).

5 Muñoz-Martín, A., Catalán, M., Martín-Dávila, J. & Carbó, A. Upper crustal structure of Deception Island area (Bransfield Strait, Antarctica) from gravity and magnetic modelling. *Antarctic Science* **17**, 213-224, doi:10.1017/S0954102005002622 (2005).

6 Catalán, M., Agudo, L. M. & Muñoz, A. Geomagnetic secular variation of Bransfield Strait (Western Antarctica) from analysis of marine crossover data. *Geophysical Journal International* **165**, 73-86, doi:10.1111/j.1365-246X.2006.02877.x (2006).

7 Galindo-Zaldívar, J., Gamboa, L., Maldonado, A., Nakao, S. & Bochu, Y. Tectonic development of the Bransfield Basin and its prolongation to the South Scotia Ridge, northern Antarctic Peninsula. *Marine Geology* **206**, 267-282, doi:10.1016/j.margeo.2004.02.007 (2004).

8 Ryan, W. B. F. *et al.* Global Multi-Resolution Topography synthesis. *Geochemistry, Geophysics, Geosystems* **10**, n/a-n/a, doi:10.1029/2008GC002332 (2009).

9 Prudencio, J. *et al.* The 3D Attenuation Structure of Deception Island (Antarctica). *Surv Geophys* **36**, 371-390, doi:10.1007/s10712-015-9322-6 (2015).

10 Luzón, F., Almendros, J. & García-Jerez, A. Shallow structure of Deception Island, Antarctica, from correlations of ambient seismic noise on a set of dense seismic arrays. *Geophysical Journal International* **185**, 737-748, doi:10.1111/j.1365-246X.2011.04962.x (2011).

11 Martí, J., Geyer, A. & Aguirre-Diaz, G. Origin and evolution of the Deception Island caldera (South Shetland Islands, Antarctica). *Bulletin of Volcanology* **75**, 1-18, doi:10.1007/s00445-013-0732-3 (2013).

12 Kusakabe, M. *et al.* Noble gas and stable isotope geochemistry of thermal fluids from Deception Island, Antarctica. *Antarctic Science* **21**, 255-267, doi:10.1017/S0954102009001783 (2009).

13 Ben-Zvi, T. *et al.* The P-wave velocity structure of Deception Island, Antarctica, from two-dimensional seismic tomography. *Journal of Volcanology and Geothermal Research* **180**, 67-80, doi:10.1016/j.jvolgeores.2008.11.020 (2009).

14 Pedrera, A. *et al.* The fracture system and the melt emplacement beneath the Deception Island active volcano, South Shetland Islands, Antarctica. *Antarctic Science* **24**, 173-182, doi:10.1017/S0954102011000794 (2012).

15 Zandomeneghi, D. *et al.* Crustal structure of Deception Island volcano from P wave seismic tomography: Tectonic and volcanic implications. *Journal of Geophysical Research* **114**, B06310, doi:10.1029/2008jb006119 (2009).

16 Ibáñez, J. M., Almendros, J., Carmona, E., Martínez-Arévalo, C. & Abril, M. The recent seismo-volcanic activity at Deception Island volcano. *Deep Sea Research Part II: Topical Studies in Oceanography* **50**, 1611-1629, doi:10.1016/S0967-0645(03)00082-1 (2003).

Deciphering the evolution of Deception Island's magmatic system

A. Geyer (1), A.M. Álvarez-Valero (2), G. Gisbert (3), M. Aulinas (4), D. Hernández-Barreña (2), A. Lobo (1), J. Marti (1)

(1) Institute of Earth Sciences Jaume Almera, ICTJA, CSIC, Lluis Sole i Sabaris s/n, 08028 Barcelona, Spain

(2) Departamento de Geología, Universidad de Salamanca, 37008 Salamanca, Spain

(3) Instituto de Geociencias CSIC-UCM, Severo Ochoa 7, 28040 Madrid, Spain

(4) Departament de Mineralogia, Petrologia i Geologia Aplicada. Universitat de Barcelona, Marti Franques s/n, 08028 Barcelona, Spain

SUPPLEMENTARY MATERIAL 8

Deception Island's magmatic system: Evolution and present stage

Evolution of Deception Island´s magmatic system: Pressure-Temperature conditions

Methodology

Pressure-Temperature conditions of the magmatic reservoirs beneath Deception Island at the different evolutionary stages were calculated using the thermodynamic database of rhyolite-MELTS software v.1.2.0[1-3] (http://melts.ofm-research.org/), whose input compositions were obtained by X-Ray Fluorescence (XRF) and Electron Microprobe (EMP) analysis of our own samples (Supplementary Materials 1 and 5). This software accounts for the phases and residual glass(es) involved in the equilibrium crystallization during the magma cooling. The range of water content we applied in the input compositions varies from 0.1 to 2%, constrained according to the total value (up to 100%) of the EMP and XRF results (Supplementary Materials 1 and 5).

Pre-caldera stage

Pressure estimates on the pre-caldera samples (e.g., DI-12, DI-50) indicate that, during this stage, some of these magmas ascended directly from depths ≥ 25 km (i.e., P > 6.5 kbar, assuming an average crustal density of 2650 kg/m^3) suggesting a mantle origin (Moho depth beneath DI is between 15 and 20 km deep[4]). The estimated stagnation depths from further evolved pre-caldera magmas indicate an equilibration depth between 15 and 20 km (P ~ 4–5 kbar) revealing the accumulation of magmatic material at the crust-mantle boundary, similar to other volcanic areas[5-8] (R1, Fig. S8-1) (e.g., DI-23, DI-48).

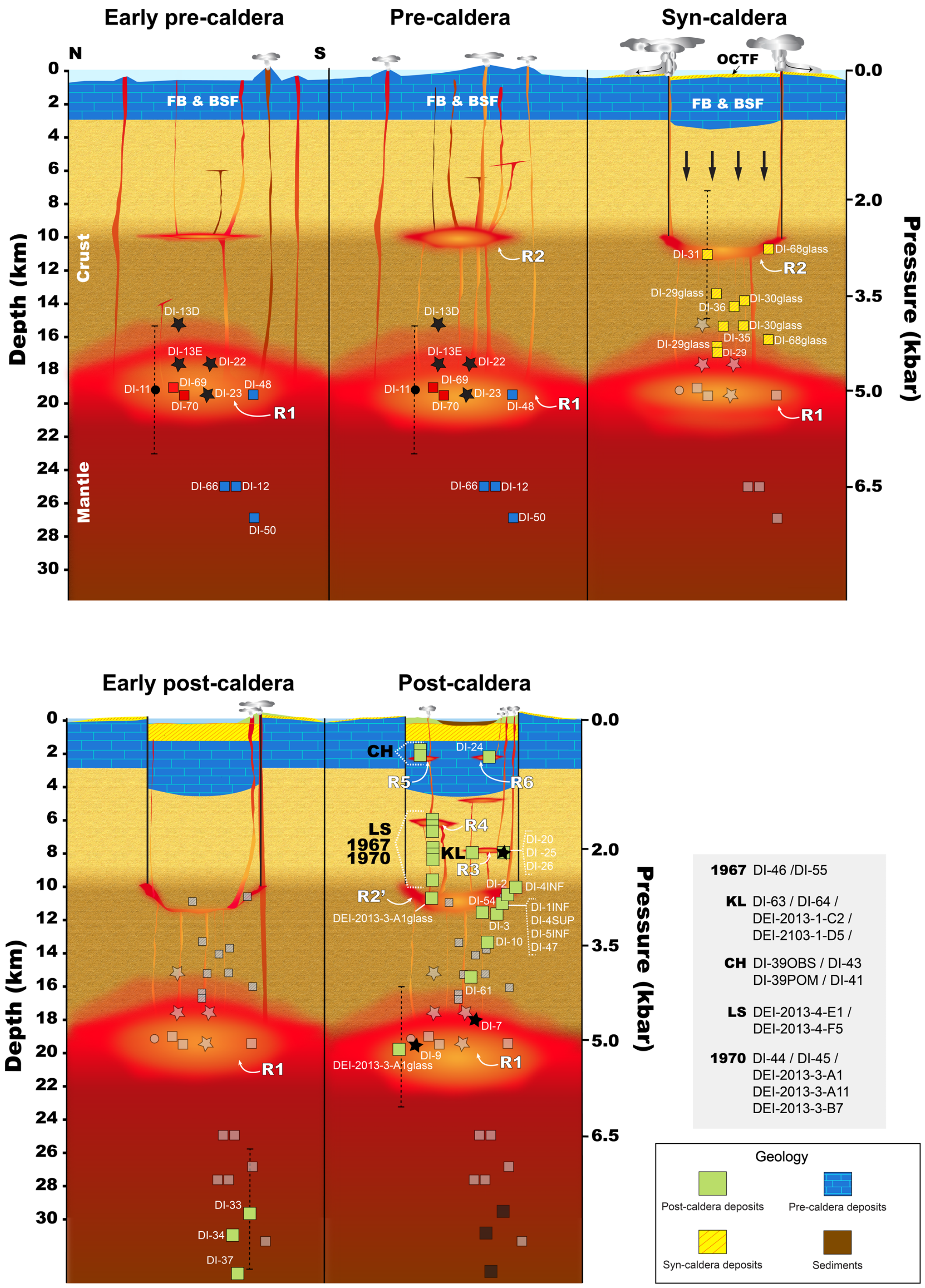

Fig. S8-1 Conceptual model of the magmatic system of Deception Island based on P-T estimates. See text for more details. CH Cross Hill eruption; KL Kroner Lake eruption; LS Lago Escondido eruption. This figure was generated with Adobe Illustrator CC 2015.3.1 (Copyright © 1987–2016 Adobe Systems Incorporated and its licensors).

Syn-caldera stage

Syn-caldera magma, mainly represented by the ignimbrite deposits of the Outer Coast Tuff Formation (OCTF), group into (Fig. S8-2): (i) a main compositional cluster that comprises most of the samples that deviate from the principal chemical trends showing, for the same SiO_2 percentage, a lower TiO_2 and FeO_t content than pre- and post-caldera stage samples (Fig. S8-2a, grey dashed circle); and ii) a minor number of samples with < 55 wt.% SiO_2 within the main DI geochemical trends. The first group would correspond to the second magma series proposed by Smellie et al.[9].

Pressure estimates of OCTF samples reveal a provenance of the syn-caldera magmas from ~ 11–19 km depth. We assert that the main compositional cluster corresponds to magmas stagnated in a shallower magma reservoir (R2, at about ~ 10–11 km depth, P ~ 3kbar), thus being directly responsible for the caldera-forming event. The accumulation depth of R2 is presumably promoted by the contact between upper and lower crust located at the same depth range[10-12]. The loading stress related to the growing basaltic shield structure would have also favored the ascending basaltic magmas to stop at shallower depths (e.g., [13]). Comparable magma stagnation pressures have been estimated for other well-known volcanic calderas (e.g., Aira, Japan[14]). The more basic OCTF samples falling outside the main cluster (yet within the main differentiation trend) would correspond to magmas coming from deeper sources, i.e., R1. This second magma would have been similar in composition to prior and subsequent magmas, and within the differentiation trend defined by the pre- and post-caldera rocks (Fig. S8-2). The arrival of these hotter and more primitive magmas into reservoir R2 may have triggered the explosive eruption leading to the caldera formation[15,16] as already suggested by Smellie et al.[9]. Arrival of deeper, hotter magma into a shallower reservoir is a common volcanic process triggering explosive eruptions (e.g., [15,16]) and has already been suggested for DI (e.g., [9,17]). In line

with other examples of caldera-forming events, all eruptible material would have been extruded from the magma chamber, fully or partially destroying R2[18-20]. Additionally, approximately linear trends in binary diagrams indicate that this reservoir was likely heterogeneous. Heterogeneity might be caused by incomplete magma mixing and/or reservoir stratification, both consistent with a supply of more primitive magmas from a deeper reservoir. Samples of the main syn-caldera rocks cluster apparently define a secondary trend in the FeO_t vs. SiO_2 diagram of Fig. S8-3b, pointing towards the cumulate composition (e.g., towards sample B.837.5 in Smellie et al.[9]). This may indicate minor cumulate assimilation or crystal accumulation in these magmas.

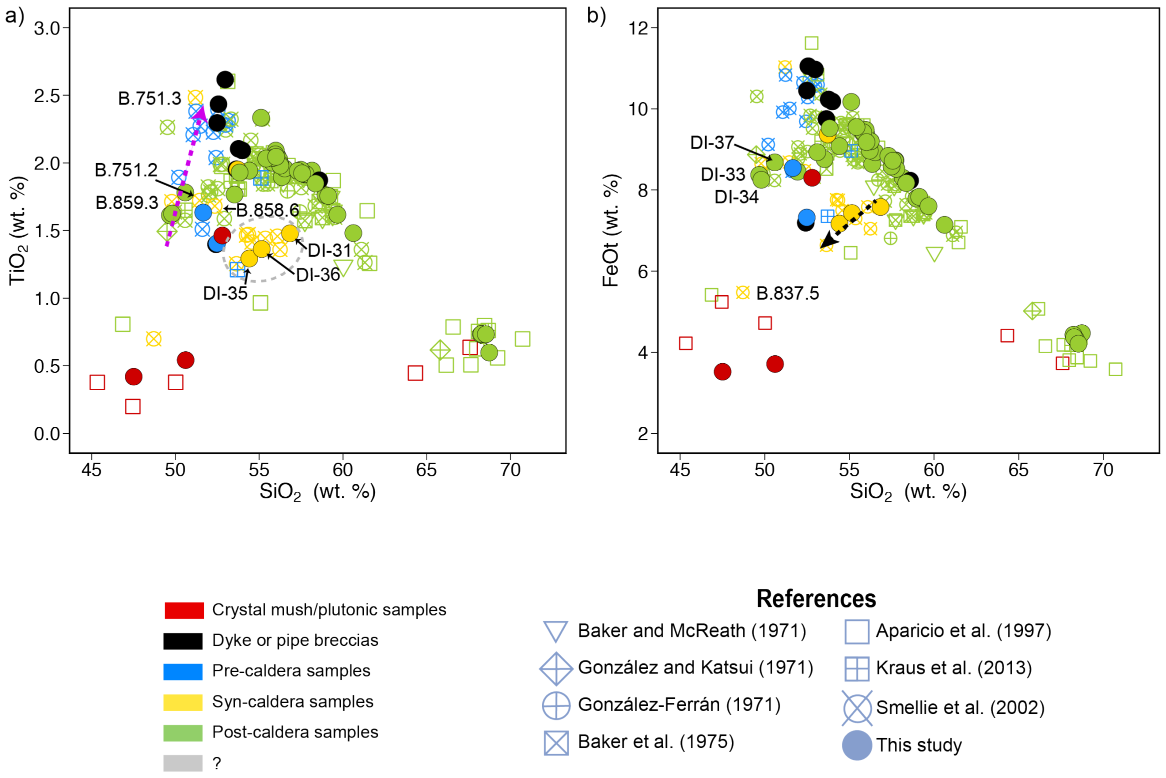

Fig. S8-2: TiO_2 (left) and FeO_t (right) vs. SiO_2 content Harker Diagrams for the studied DI rocks (see Supplementary Materials 1-2 for details on composition and exact latitude-longitude coordinates of the rock samples). Major element contents are normalized to 100 wt.% in an anhydrous base. Purple and black dashed arrows suggest trends of potential magma differentiation. This figure was generated with RStudio Version 1.0.143 (https://www.rstudio.com/) using ggplot2 package Version 2.1.9000 (http://www.ggplot2.org), a plotting system for R. Final layout of this figure was achieved using Adobe Illustrator CC 2015.3.1 (Copyright © 1987–2016 Adobe Systems Incorporated and its licensors).

Post-caldera stage

The first magmas erupted after the caldera-forming eruption, mainly represented as diluted pyroclastic density currents deposited over the OCTF (e.g., DI-33, DI-34, and DI-37), are amongst the most primitive analysed in this suite (Fig. S5-2 and S8-2b). P-T estimates of these magmas (P > 7.5 kbar, > 28.5 km) suggest a direct ascent from the mantle magma source (Fig. S8-1). The time span between the deposition of the syn-caldera deposits and the eruption of these magmas is still uncertain. This hinders the full understanding of genesis of these magmas: (i) magmas coeval to the end of the caldera-forming event may either represent the most primitive of the deepest basic magmas or unrelated magmas that triggered the caldera-forming eruption favoured by the opening and depressurization of the plumbing system during eruption; (ii) magmas ejected after a significant time, imply a direct ascent without stagnation in intermediate depths through the formation of new ascent paths outside the reservoir areas, or ascent through inactive reservoirs (e.g., collapsed during caldera formation or significantly solidified).

Magma compositions and P-T estimates of juvenile samples from the late post-caldera stage, including historical eruptions, demonstrate the current existence of a complex magmatic system beneath DI (Fig. S8-1). Our results, in line with Smellie et al.[9] indicate that the wide compositional range in the relatively small volume eruptions would reflect the existence of multiple small compositionally stratified feeding magma chambers and a larger melt accumulation zone at depth that replenishes them.

P-T calculations of collected dyke samples (e.g., DI-7, DI-9) and of other juvenile fragments belonging to different recent post-caldera eruptions (e.g., the 1970 eruption, DI-44, DI-45) suggest that erupted magma can either rise directly from the deep magma source at the crust-mantle boundary R1 or be supplied by diverse small magma chambers

at distinct shallow depths (R3–R6) (Fig. S8-1). In this sense, magma stagnation in shallower reservoirs within a cooler country rock (e.g., R5 or R6) promotes faster and larger differentiation, thus generating the most evolved magma compositions in the DI system (e.g., Cross Hill: DI-39OBS / DI-39POM / DI-41). In addition, juvenile samples from eruption(s) that occurred around Crater Lake area, on the southern slope of Mt. Kirkwood (e.g., DI-1INF, DI-4SUP), point out the existence of a magma source located at similar depths as R2. Considering that this reservoir (R2) was supposed to be fully or partially destroyed during the caldera-forming event, we interpret that, since the caldera collapsed, new reservoir(s) (R2′) have formed. In the latter case, these would be located at similar depths as R2.

Our conceptual model for the magmatic system under DI is coherent with the one proposed by Baker et al.[21]. They suggested that the 1967, 1969 and 1970 eruptions (~ 0.2 km^3 combined volume) were fed by a small volume of magma, probably emplaced into the upper part of the ring-fault system in a dike-like body, thus favouring relatively rapid cooling and differentiation. The wide compositional ranges of the 1967 and 1970 eruptions, which depict approximately linear trends in binary diagrams, are interpreted as related to magma mingling and mixing, as well as to reservoir stratification[21] (Supplementary Material 5). In contrast to magma variability during the 1967 and 1970 eruptions, some tuff cone clusters extending laterally a few kilometres emitted compositionally very restricted magmas[9], indicating that compositionally more homogeneous magma reservoirs are also present in the system.

Fractional crystallization modelling with rhyolite-MELTS

Modelling with rhyolite-MELTS software v.1.2.0 [1-3] was performed to test consistency of trends observed in the DI suite with magma differentiation through fractional crystallization processes. Results also provide information on the most likely H_2O content, P, and fO_2 conditions under which magmas evolved. Differentiation by fractional crystallization of starting compositions in the mafic (e.g., B.751.5a from Smellie et al [9]) and intermediate (e.g., DI-4sup) areas was modelled for a range of initial H_2O content of 0–1.25 wt.%, pressure at 1–5 kbar, and with both fO_2 fixed (0 to 2 log units) and free relative to the Quartz-Fayalite-Magnetite (QFM) buffer. Starting compositions were chosen within the mafic and intermediate areas of the differentiation trends, with no elements at outlier positions.

Main results indicate that tholeiitic major element trends are consistent with evolution through fractional crystallization of basaltic melts with an initial 0.5–0.75 wt.% H_2O under fO_2 conditions of 0 to 1 log units (QFM) at pressures from 2 to 5 kbar (Fig. S8-3). Results for the first section of the trend (up to 56 wt.% SiO_2) indicate that under the same initial H_2O content and fO_2, maximum TiO_2 enrichment is controlled by pressure (Fig. S8-3). At an initial 0.5 wt. % H_2O and fO_2 = 1 (QFM), TiO_2 enrichments similar to those of the most evolved post-caldera samples (2 wt.% TiO_2 at 55 wt.% SiO_2, e.g., DI-4SUP, DI-26) are produced by differentiation at 2-3 kbar (depths of the shallowest magma bodies, R2′ -R4). Higher TiO_2 enrichments (2.6 wt.% TiO_2 at 53 wt.% SiO_2, e.g., DI-7, DI-9) correspond to the samples at higher pressures of c. 5 kbar (depth of R1).

Differentiation through 57 wt.% fractional crystallization of sample B.751.5a[9] with an initial 0.5 wt.% H_2O at 3 kbar and fO_2 = 1 log unit matches the compositions of samples in the middle compositional range of post-caldera rocks (e.g., DI-4SUP and DI-

26) (Table S8-1). The relative difference between the observed DI-4SUP and modelled composition is below 10% for all major elements except Na_2O. The modelled fractionating assemblage consists of clinopyroxenes (Cpx), plagioclases (Pl), and spinels (Spl).

The most evolved section of the differentiation trend (> 56 wt.% SiO_2) has been modelled using both starting compositions of B.751.5a and DI-4SUP. In this case, significant differences in Al_2O and CaO contents are mostly related to the fractionation of two Cpx (Ca-rich and Ca-poor) in the model compared to the fractionation of only one Ca-rich Cpx phase, and to the inefficient Pl fractionation in natural rocks. Differentiation degrees matching those of sample OG. 226 from González and Katsui[22] are obtained through 82.7 wt.% fractional crystallization of sample B.751.5a (H_2O=0.5 wt.%, P=3 kbar, initial fO_2 = 1) and 61.4 wt.% fractional crystallization of DI-4SUP (H_2O=1 wt.%, P=2 kbar, initial fO_2 = 1). Relative differences between modelled and natural compositions are provided in Table S8-1. Modelled fractionating mineral assemblage for samples B.751.5a and DI-4SUP consists of Cpx, Pl, oxides (opaques and Spl) and late apatite (Apt) and olivine (Ol) for the former, and Cpx, Pl, rhombohedral oxides, Spl, and minor Apt for the later.

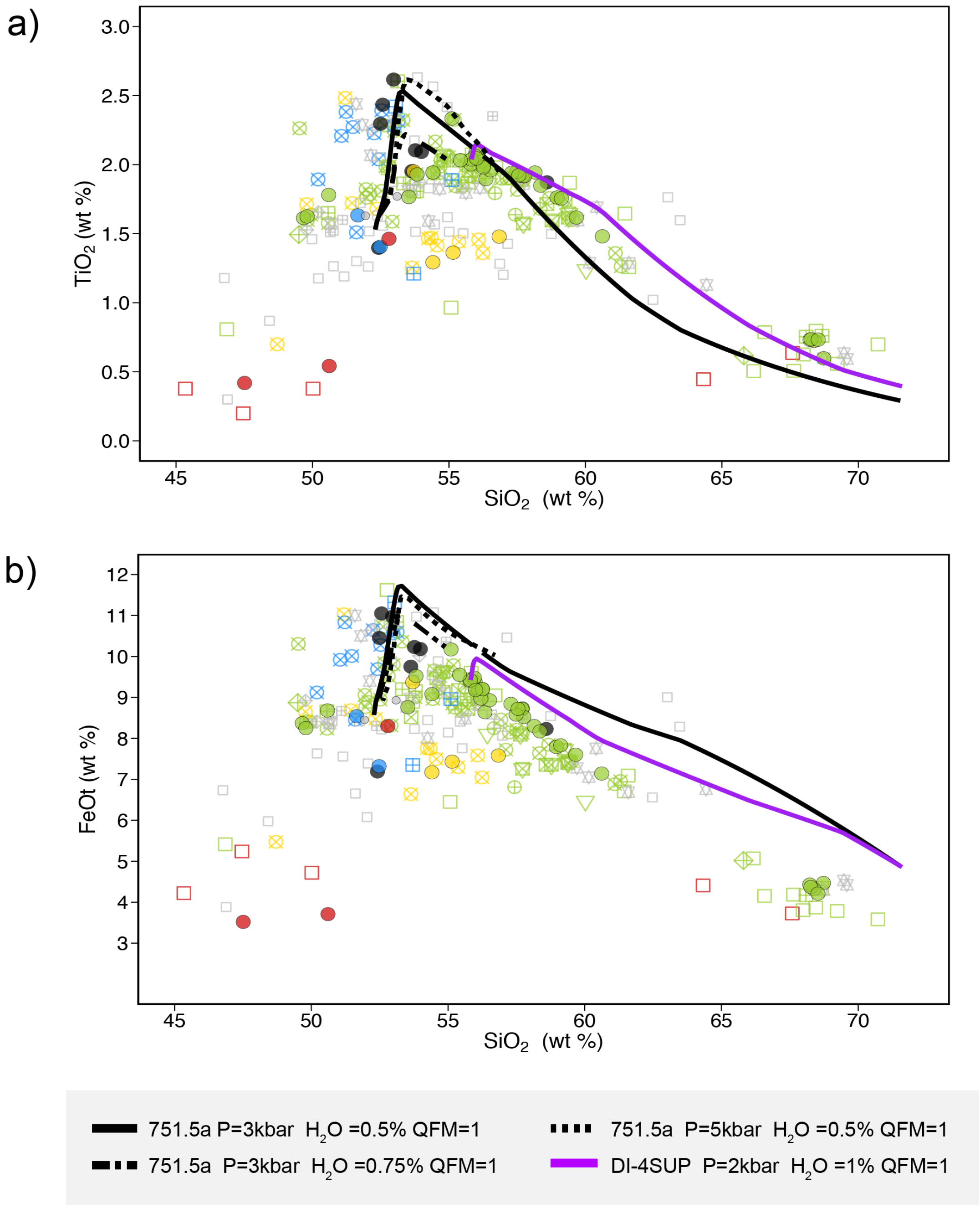

Fig. S8-3: TiO_2 and FeO_t vs. SiO_2 diagrams highlighting the thermodynamic modelling results of representative samples (see text for details). Generated with RStudio Version 1.0.143 (https://www.rstudio.com/) using ggplot2 package Version 2.1.9000 (http://www.ggplot2.org). Final layout with Adobe Illustrator CC 2015.3.1 (Copyright © 1987–2016 Adobe Systems Incorporated and its licensors). Legend as per Fig. S8-2.

	A. First Section (< 56 wt.% SiO_2)				B. Second Section (> 56 wt.% SiO_2)						
	Start c.	Compared c.	Modelled c.	Rel. Err.	Starting compositions		Compared c.	Modelled compositions and relative error			
	**B.751.5a**	**DI-4SUP**			**B.751.5a**	**DI-4DUP**	**OG- 226**	**from B.751.5a**	**rel. Err.**	**from DI-4SUP**	**rel. Err.**
**SiO_2**	52.48	56.12	56.38	0.46	52.48	56.12	66.87	68.67	2.68	69.12	3.35
**TiO_2**	1.54	2.05	2.14	4.34	1.54	2.05	0.63	0.49	-21.85	0.62	-1.78
**Al_2O_3**	17.01	16.07	14.5	-9.85	17.01	16.07	15.68	9.16	-41.59	9.85	- 37.17
**FeO_t**	8.6	9.47	10.36	9.39	8.6	9.47	5.1	6.55	28.44	6.03	18.31
**MgO**	6.13	3.48	3.39	-2.42	6.13	3.48	0.64	0.66	3.5	0.87	36.81
**CaO**	9.92	6.94	6.48	-6.72	9.92	6.94	2.42	3.93	62.23	4.52	86.51
**Na_2O**	3.96	5.05	5.98	18.37	3.96	5.05	6.94	8.73	25.86	7	0.86
**K_2O**	0.35	0.82	0.79	-3.45	0.35	0.82	1.73	1.81	5.07	2	15.54

Table S8-1: Major element results of fractional crystallization modelling using MELTS normalized to anhydrous 100 wt.%. For the first section of differentiation trend (A) modelled composition was obtained after 57.0 wt.% fractional crystallization of sample B.751.5a from Smellie et al.[9] with initial conditions H_2O=0.5 wt.%, P=3 kbar, fO_2= 1 log unit above QFM. For the second section of differentiation trend (B) modelled compositions were obtained after: i) 82.7 wt.% fractional crystallization of sample B.751.5a with initial conditions H_2O=0.5 wt.%, P=3 kbar, fO_2= 1 log unit above QFM; and ii) 61.4 fractional crystallization of sample DI-4SUP with initial conditions H_2O=1, P=2 kbar, fO_2= 1 log unit above QFM. In all cases fO_2 was not constrained during modelling.

Linear Discriminant Analysis

Methodology

Linear Discriminant Analysis (LDA)[23,24] was performed on whole rock major element compositions of our own rock samples (Supplementary Material 1) to evaluate the consistency of the proposed conceptual model for DI's magmatic system in terms of geochemical composition (Fig. S8-1). For this, we first assigned each of the analyzed samples to one of the defined magmatic reservoirs (i.e., Deep mantle source –M, Crust-Mantle boundary magmatic source R1, or shallower reservoirs R2-R6) (Supplementary Material 1).

Given a matrix of *cases* × *descriptors* (i.e., in our study, rock samples × major elements concentrations), LDA is a supervised classification method that uses expert-defined groups (i.e., the magmatic sources defined in this study: M, R1–R6) in a sub-set of cases ("training subset") to calculate a linear transformation of the descriptors to maximize discrimination among groups. This transform is subsequently applied to all data, which are then classified into the groups. The classification is evaluated using a "testing subset" (a set of samples for which the grouping is known but that have not been included in the training subset), or, if data are scarce, by cross-validation[23].

LDA relies on an expertise (the definition of groups) that is external to the—in our case, geochemical—data to calculate components that are tuned to discriminate in accordance to a previously defined qualitative model. As a machine-learning method, LDA is very sensitive to the correctness and typicality of group adscriptions in the training phase and we have used this characteristic to check the consistency of our conceptual grouping. In particular, with scarce samples, few erroneous adscriptions or

atypical cases in the training phase inflate the within-group variances and impact the resulting transformation and classification. A first run of LDA can be used to point out incorrectly ascribed or atypical samples within their respective groups. Once user-defined adscriptions in the training set are certain, LDA can be used to assess whether the grouping defined by the training set is consistent in terms of the descriptor variables for the rest of samples.

A first LDA run and its resulting classification (using cross-validation) pointed out those samples that were not classified to the initially guessed reservoir; we ran LDA again without those samples to eliminate potentially erroneous initial adscriptions and atypical cases from the calculation of the LD components. We projected discarded samples onto the LD space and classified them into their nearest class. All samples were plotted in the plane defined by the first two LD components and we used this ordination and the average composition of the classes to assess the geochemical consistency of the proposed model and to discuss the implications of both clustered and isolated samples.

Results

LDA brings into evidence the general consistency of the proposed model of magma reservoirs in terms of major element geochemistry (Fig. S8-4). All suggested reservoirs but R3 have a distinct composition and are ordered from Mantle to R6 along the major LD axis. R5 samples have the most distinct composition, with sample DI-24 (R6) being closer, but still clearly not clustered. This provides solid evidence for the actual existence of the proposed R6 reservoir.

Samples initially guessed to belong to R3 are actually classified as either R2' or R4, and most of them are intermediate between the core of the R4 and R2' groups. This

clearly indicates the compositional similarity between R3 and R4, both reservoirs most probable repetitively fed by R2'. The wide compositional ranges of the 1967 and 1970 eruptive materials (all assigned to R4), interpreted as related to magma mingling and mixing, and reservoir stratification[21] (Supplementary Material 5) is also reflected in the LDA. The arrival of R2' material to the shallower chambers (e.g. R4) is supported also by the fact that, for some specific samples (e.g. DI-56), P-T estimates indicate a deeper magma provenance in the depth range of R2'.

Four Mantle and two R1 samples are also intermediate between the Mantle and R1 groups, with samples DI-66, DI-16B, DI-52, and DI-12, initially presumed to be of mantle origin, but actually classified as R1. This is consistent with the proposed deep position of the R1 reservoir.

Some individual samples act as outliers, not being close to any of the groups. Sample DI-70 (presumed to be R1) and DI-67 (presumed to be Mantle) are classified as Mantle but lie very far up from the Mantle centroid in the LDA plot (Fig. S8-4). These samples have higher Al_2O_3 and CaO concentrations than the average Mantle samples, with SiO_2 concentrations being, respectively, higher and lower than the average Mantle samples (Supplementary Material 1). Sample DI-26 (presumed to be R3) is classified by the LDA as R1 (Fig. S8-4). However, the sample lies far down the R1 centroid, with higher concentrations of SiO_2, Na_2O, K_2O, and P_2O_5 than the average R1 samples (Supplementary Material 1).

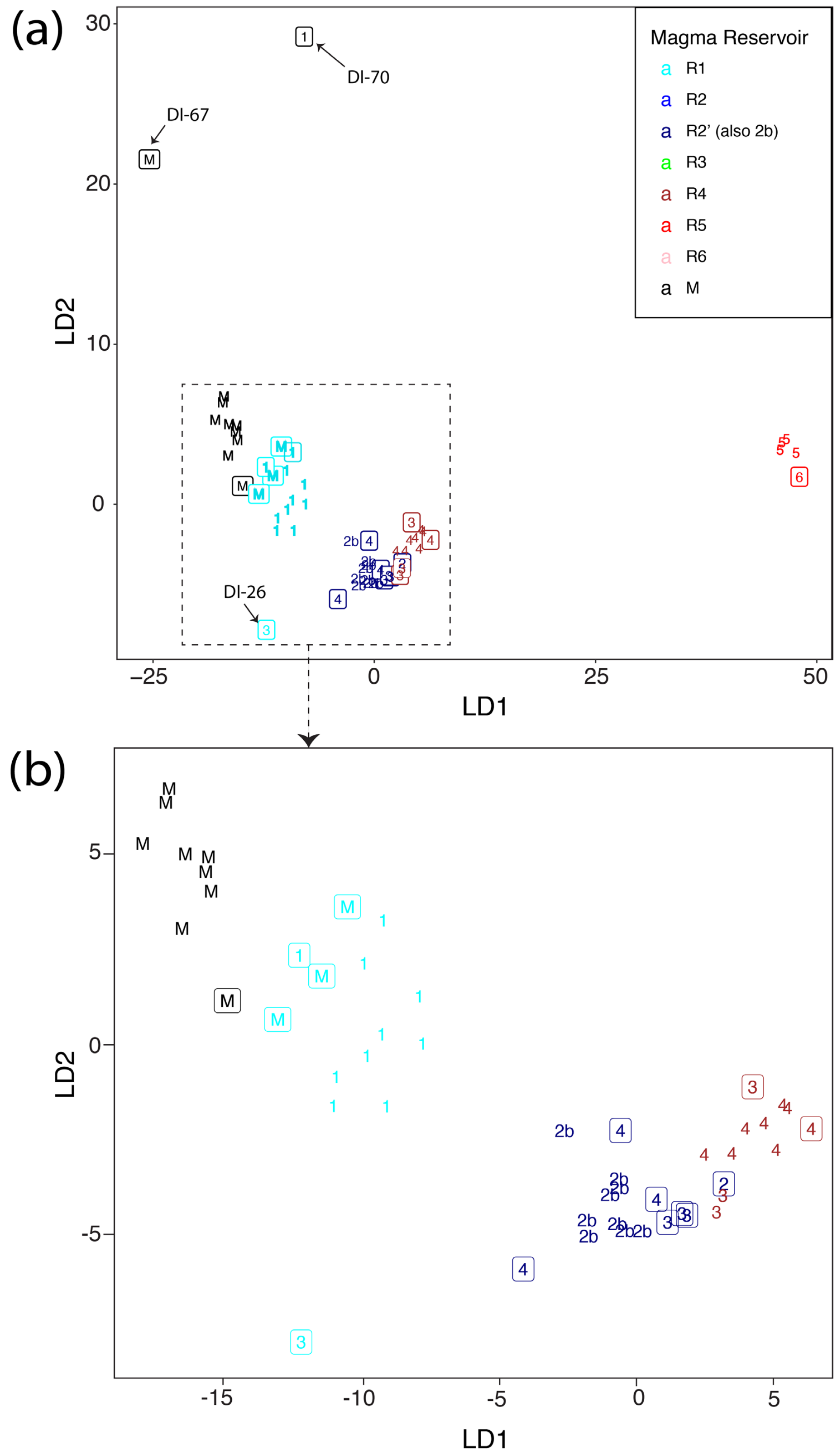

Fig. S8-4: Ordering of samples on the plane defined by the first two LD components. Labels correspond to Magma Reservoirs according to the proposed model. Colors correspond to the LDA classification. Samples within squares are those not having been included in the calculation of LD components but projected on them and attributed to their closest class.

References

1 Ghiorso, M. S. & Sack, R. O. Chemical mass transfer in magmatic processes IV. A revised and internally consistent thermodynamic model for the interpolation and extrapolation of liquid-solid equilibria in magmatic systems at elevated temperatures and pressures. *Contributions to Mineralogy and Petrology* **119**, 197-212, doi:10.1007/bf00307281 (1995).

2 Asimow, P. D. & Ghiorso, M. S. Algorithmic modifications extending MELTS to calculate subsolidus phase relations. *American Mineralogist* **83**, 1127-1132, doi:10.2138/am-1998-9-1022 (1998).

3 Gualda, G. A. R., Ghiorso, M. S., Lemons, R. V. & Carley, T. L. Rhyolite-MELTS: a Modified Calibration of MELTS Optimized for Silica-rich, Fluid-bearing Magmatic Systems. *Journal of Petrology*, doi:10.1093/petrology/egr080 (2012).

4 Christeson, G. L., Barker, D. H. N., Austin, J. A. & Dalziel, I. W. D. Deep crustal structure of Bransfield Strait: Initiation of a back arc basin by rift reactivation and propagation. *Journal of Geophysical Research: Solid Earth* **108**, doi:doi:10.1029/2003JB002468 (2003).

5 Dañobeitia, J. J. & Canales, J. P. Magmatic underplating in the Canary Archipelago. *Journal of Volcanology and Geothermal Research* **103**, 27-41, doi:10.1016/S0377-0273(00)00214-6 (2000).

6 Klügel, A., Hansteen, T. H. & Galipp, K. Magma storage and underplating beneath Cumbre Vieja volcano, La Palma (Canary Islands). *Earth and Planetary Science Letters* **236**, 211-226 (2005).

7 Lodge, A., Nippress, S. E. J., Rietbrock, A., García-Yeguas, A. & Ibáñez, J. M. Evidence for magmatic underplating and partial melt beneath the Canary Islands derived using teleseismic receiver functions. *Physics of the Earth and Planetary Interiors* **212–213**, 44-54, doi:10.1016/j.pepi.2012.09.004 (2012).

8 Martí, J., Villaseñor, A., Geyer, A., López, C. & Tryggvason, A. Stress barriers controlling lateral migration of magma revealed by seismic tomography. **7**, 40757, doi:10.1038/srep40757 (2017).

9 Smellie, J. L. *et al*. Geology and geomorphology of Deception Island. *BAS GEOMAP Series, Sheets 6-A and 6-B, 1:25 000*,78pp. with accompanying maps, (British Antarctic Survey, Cambridge, 2002).

10 Grad, M., Guterch, A. & Janik, T. Seismic structure of the lithosphere across the zone of subducted Drake plate under the Antarctic plate, West Antarctica. *Geophysical Journal International* **115**, 586-600, doi:10.1111/j.1365-246X.1993.tb01209.x (1993).

11 Grad, M., Guterch, A. & Sroda, P. Upper crustal structure of Deception Island area, Bransfield Strait, West Antarctica. *Antarctic Science* **4**, 469-476, doi:10.1017/S0954102092000683 (1992).

12 Grad, M., Shiobara, H., Janik, T., Guterch, A. & Shimamura, H. Crustal model of the Bransfield Rift, West Antarctica, from detailed OBS refraction experiments.

Geophysical Journal International **130**, 506-518, doi:10.1111/j.1365-246X.1997.tb05665.x (1997).

13 Muller, J. R., Ito, G. & Martel, S. J. Effects of volcano loading on dike propagation in an eastic half-space. *Journal of Geophysical Research* **106**, 11101-11113, doi:10.1029/2000JB900461 (2001).

14 Aramaki, S. Formation of the Aira caldera, southern Kyushu, 22.000 years ago. *Journal of Geophysical Research* **89**, 8485-8501, doi:10.1029/JB089iB10p08485 (1984).

15 Sparks, S. R. J., Sigurdsson, H. & Wilson, L. Magma mixing: a mechanism for triggering acid explosive eruptions. *Nature* **267**, 315-318, doi:10.1038/267315a0 (1977).

16 Pallister, J. S. *et al*. Magma mixing at Pinatubo volcano: petrographic and chemical evidence from the 1991 deposits., in *Fire and Mud: Eruptions and Lahars of Mount Pinatubo, Philippines* (eds C.G. Newhall & R.S. Punongbayan) 687–731 (PHIVOLCS, University of Washington Press, 1996).

17 Smellie, J. L., Hofstetter, A. & Troll, G. Fluorine and boron geochemistry of an ensialic marginal basin volcano: Deception Island, Bransfield Strait, Antarctica. *Journal of Volcanology and Geothermal Research* **49**, 255-267, doi:10.1016/0377-0273(92)90017-8 (1992).

18 Folch, A., Codina, R. & Martí, J. Numerical modeling of magma withdrawal during explosive calder-forming eruptions. *Journal of Geophysical Research* **106**, 16163-16175, doi:10.1029/2001JB000181 (2001).

19 Martí, J., Folch, A., Macedonio, G. & Neri, A. Pressure evolution during caldera forming eruptions. *Earth and Planetary Science Letters* **175**, 275-287, doi:10.1016/S0012-821X(99)00296-4 (2000).

20 Folch, A. & Martí, J. Time-dependent chamber and vent conditions during explosive caldera-forming eruptions. *Earth and Planetary Science Letters* **280**, 246-253, doi:10.1016/j.epsl.2009.01.035 (2009).

21 Baker, P. E., McReath, I., Harvey, M. R., Roobol, M. J. & Davies, T. G. The geology of the south Shetland islands: Volcanic evolution of Deception island. *British Antarctic Survey Scientific Reports* **78**, 81 pp. (1975).

22 González-Ferrán, O. & Katsui, Y. Estudio integral del volcanismo cenozoico superior de las Islas Shetland del Sur, Antartica. *Ser. cient. Inst. antart. chileno* **22**, 123-174 (1971).

23 James, G., Witten, D., Hastie, T. & Tibshirani., R. An Introduction to Statistical Learning with Applications in R. . *Springer Texts in Statistics*,426 pp., (Springer-Verlag New York, 2013).

24 Reimann, C., Filzmoser, P., Garrett, R. G. & Dutter, R. Statistical Data Analysis Explained: Applied Environmental Statistics with R. *Statistical Data Analysis Explained*,343 pp., (John Wiley & Sons, Ltd, 2008).

Deciphering the evolution of Deception Island's magmatic system

A. Geyer (1), A.M. Álvarez-Valero (2), G. Gisbert (3), M. Aulinas (4), D. Hernández-Barreña (2), A. Lobo (1), J. Marti (1)

(1) Institute of Earth Sciences Jaume Almera, ICTJA, CSIC, Lluis Sole i Sabaris s/n, 08028 Barcelona, Spain

(2) Departamento de Geología, Universidad de Salamanca, 37008 Salamanca, Spain

(3) Instituto de Geociencias CSIC-UCM, Severo Ochoa 7, 28040 Madrid, Spain

(4) Departament de Mineralogia, Petrologia i Geologia Aplicada. Universitat de Barcelona, Marti Franques s/n, 08028 Barcelona, Spain

SUPPLEMENTARY MATERIAL 9

Numerical simulations of cooling magma reservoirs

Methodology

Theory and equations

For the sake of simplicity, we consider the modeled magma chamber to be emplaced immediately before it begins cooling[1-4]. The internal temperature distribution of the magma chamber is calculated using the Finite Element (FE) method, by solving the heat transfer equation, assuming as negligible the effect of viscous heating and pressure-volume work:

$$\rho\, Cp\, \frac{\partial T}{\partial t} + \rho\, Cp\, \boldsymbol{u}\, \nabla T = \nabla(k \nabla T) + \boldsymbol{Q} \qquad (1)$$

where the equation parameters refer to density (ρ), specific heat capacity at constant pressure (Cp), temperature (T), time (t), the velocity vector ($\boldsymbol{u}$), thermal conductivity (k) and **Q** contains heat sources other than viscous heating (see Table S9.1 for more details concerning thermal and physical parameters). Since we are dealing here with pure conductive heat transfer (i.e., **u**=0), Equation (1) can be rearranged as follows:

$$\rho\, Cp\, \frac{\partial T}{\partial t} + \nabla(-k \nabla T) = \boldsymbol{Q} \qquad (2)$$

The geometric modelling, mesh discretization and numerical computations were carried out with the COMSOL Multiphysics v5.2a software package (http://www.comsol.com). To simulate the solidifying magma, we use the *heat transfer with phase change* module. The latter allows solving the heat equation after setting the properties of a phase-change material (from liquid to solid) according to the Apparent Heat Capacity formulation (AHC)[5-7]. The AHC formulation is considered to be the best representation of a naturally occurring wide phase-change temperature interval, such as

what happens during magma cooling[1]. The latent heat of crystallization is accounted for by increasing the heat capacity of the material within the phase change temperature range. The reader is referred to the work by Rodriguez et al.[1] for more details on the methodology. A comprehensive report detailing one of the models, which includes all settings within COMSOL (e.g., model properties; physics settings; geometry; mesh), as well as an example of one of the models, are available from the corresponding author upon request (COMSOL Multiphysics commercial software V5.2a or greater, and the *heat transfer with phase change module* are required).

Model geometry and mesh

The performed FE models are axisymmetric and are constructed over a cylindrical coordinate system with positive *z* values related to altitudes above sea level (Fig. S9-1). The magma chamber geometry is oblate with height, *h* and width, *w* (Fig. S9-1a). Taking into account the axial symmetry, the reservoir volume, *V* is calculated as follows:

$$V = \frac{4}{3}\pi\, h\, w^2 \tag{3}$$

The computational domain corresponds to a section of the crust with a 40 km radius stretching to a depth of 30 km below sea level (Fig. S9-1a). The FE mesh consists of about 15,000 linear triangular elements up to 1.5 km in size farther from the magma reservoir, and 10 m in size near the edge of the magma chamber (Fig. S9-1b). Time steps of the performed calculations range from few days up to 3,000 years depending on the

model and the stage of the cooling process. We have checked that the selected time steps at the beginning of the cooling processes do not affect the results obtained.

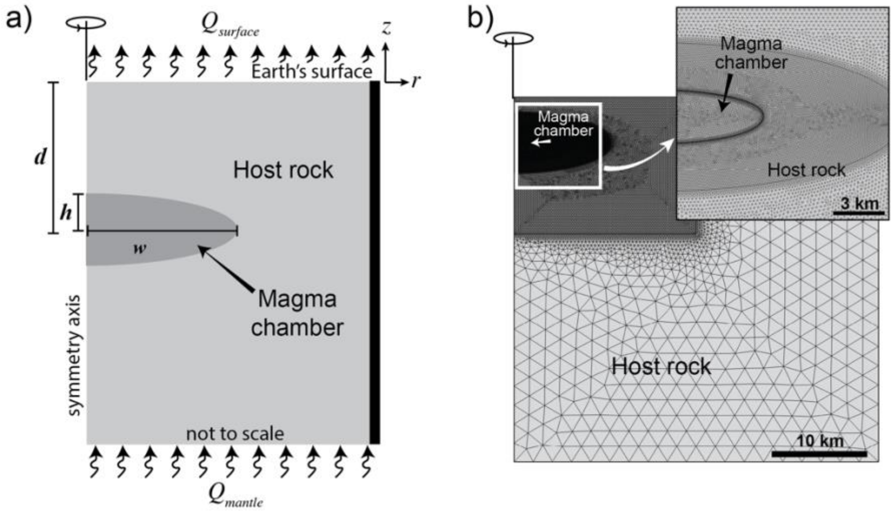

Figure S9-1: (a) Sketch of the numerical model set-up, with applied boundary conditions indicated. (b) Example of the Finite Element mesh used for modeling runs. See Table S9-1 and the text for further explanation of the different parameters. This figure was generated with Adobe Illustrator CC 2015.3.1 (Copyright © 1987–2016 Adobe Systems Incorporated and its licensors).

Table S9.1: Physical and thermal parameters used in the numerical simulations

Symbol	Value	Variable	SI Unit
Cp_{crust}	$342 + 1.774\,T^{*} - 1.25\ \times 10^{-3}\,T^{*2} + 3.2 \times 10^{-7}\,T^{*3}$	Specific heat capacity of crust[3]	J/kg°C
Cp_{melt}	Supplementary Material 1	Specific heat capacity of magma (melt phase)	J/kg°C
Cp_{solid}	Supplementary Material 1	Specific heat capacity of magma (solid phase)	J/kg°C
w	140–2880	Magma chamber width	m
h	30–1150	Magma chamber height	m
κ_{magma}	*f*(T) [2]	Magma thermal diffusivity[8]	m^2/s
κ_{crust}	*f*(T) [2]	Crust thermal diffusivity[8]	m^2/s
k_{magma}	$k_{magma} = \kappa_{magma}\,\rho_{magma}\,Cp_{magma}$	Magma thermal conductivity[8]	W/m°C
k_{crust}	$k_{crust} = \kappa_{crust}\,\rho_{crust}\,Cp_{crust}$	Crust thermal conductivity[8]	W/m°C
L	400	Latent heat of crystallization[3]	kJ/kg
φ	Supplementary Material 1	Melt fraction	-
ρ_{crust}	2650	Crust density	kg/m^3
ρ_{magma}	2550	Average magma density	kg/m^3
θ	1- φ	Solid fraction	-
R	*h/ w,* 0.1-0.8	Magma chamber aspect ratio	-
thermal_grad	30	Crustal thermal gradient	°C/km
T_liquidus	Supplementary Material 1	Magma liquidus temperature	°C
T_solidus	Supplementary Material 1	Magma solidus temperature	°C
T_surface	5	Surface temperature	°C
V	$10^7 – 10^{10}$	Magma chamber volume	m^3

Physical and thermal properties and boundary conditions

The selected starting magmatic compositions for the numerical simulations corresponds to the sample DI-4SUP, the same used for the fractional crystallization models (Supplementary Material 8). The melt (θ) and solid (ϕ) fractions, as well as the thermal properties of the crystallizing magmas, are determined using the rhyolite-MELTS software v.1.2.0 [9-11]. Results for isobaric cooling at 1, 2, and 3 kbar pressure, with fO_2 1 log unit above the Quartz-Fayalite-Magnetite (QFM) oxygen buffer and 1% of H_2O, are reported in Supplementary Material 1.

Using the thermal properties provided by MELTS, we explicitly account for the temperature dependence of thermal diffusivity (κ) and heat capacity (C_P). Incorporating a temperature-dependent diffusivity is critical due to its strong influence on the temperature-dependence of thermal conductivity ($k=\rho \cdot C_P \cdot \kappa$) at high temperatures[12]. The average magma density ρ_{magma} obtained from MELTS is 2550 kg/m^3. For the crustal density ρ_{crust}, we consider an average value of 2650 kg/m^3 [13].

The input temperature profile for the country rock is a typical geothermal gradient of 30°C/km[13]. We assume a relatively uniform lateral temperature profile, and consider the surrounding walls of the country rock to have no heat flux (i.e., the outer edge of the rotationally symmetric model is insulating). The subcrustal mantle heat flow Q_{mantle} = 0.03 W/m^2 and the surface heat flow $Q_{surface}$ = 0.07 W/m^2 are assigned to the bottom and top limits of the computational domain, respectively (Fig. S9-1a) (Table S9-1). For the magma chamber, we assume an initial temperature T_{0_magma} equivalent to the liquidus temperature of the magma (Supplementary Material 1).

Results

We ran a total of 48 simulations considering—from small (V = 0.01 km^3) to large volume (V =10 km^3)—magma chambers of four distinct geometries. Reservoir geometry is defined by the aspect ratio R expressed as h/w (Fig. S9-2). Values of $R \approx 1$ correspond to spherical or quasi-spherical reservoirs and $R << 0.5$ to sills. Magma chambers are emplaced at different depths equivalent to 1, 2, or 3 kbar stagnation pressures. The main results are represented in Figures S9-3 to S9-5.

Reservoir geometry, size, and depth are primary factors controlling the timing of the cooling process. The stagnation depth of the magma reservoir may decrease (for shallower reservoirs) or increase (for deeper reservoirs) the cooling time by several orders of magnitude due to the changes in the host rock temperature (Fig. S9-3).

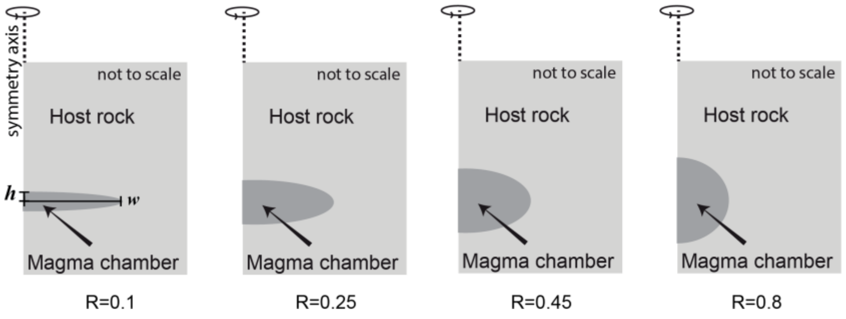

Figure S9-2: Sketch of the different numerical model set-ups considering diverse magma chamber geometries. The reservoir aspect ratio R, defined as h/w, varies from 0.1 (sill-like chamber) to 0.8 (quasi-spherical chamber). This figure was generated with Adobe Illustrator CC 2015.3.1 (Copyright © 1987–2016 Adobe Systems Incorporated and its licensors).

For the same magma geometry and stagnation depth, all magma contained in small reservoirs (i.e., V= 0.01 km^3) may reach solidus temperatures in few hundreds of years (< 500 years) after emplacement (Fig. S9-4). By contrast, magma accumulated in larger reservoirs (i.e., V= 1 km^3) may need from a few thousand to several tens of thousands of

years to be fully crystallized (Fig. S9-4). In the case of reservoirs of similar volume but different shape, sill-like geometries tend to accelerate the cooling process allowing small chambers to fully crystallize in few hundred years (Fig. S9-5 and S9-6).

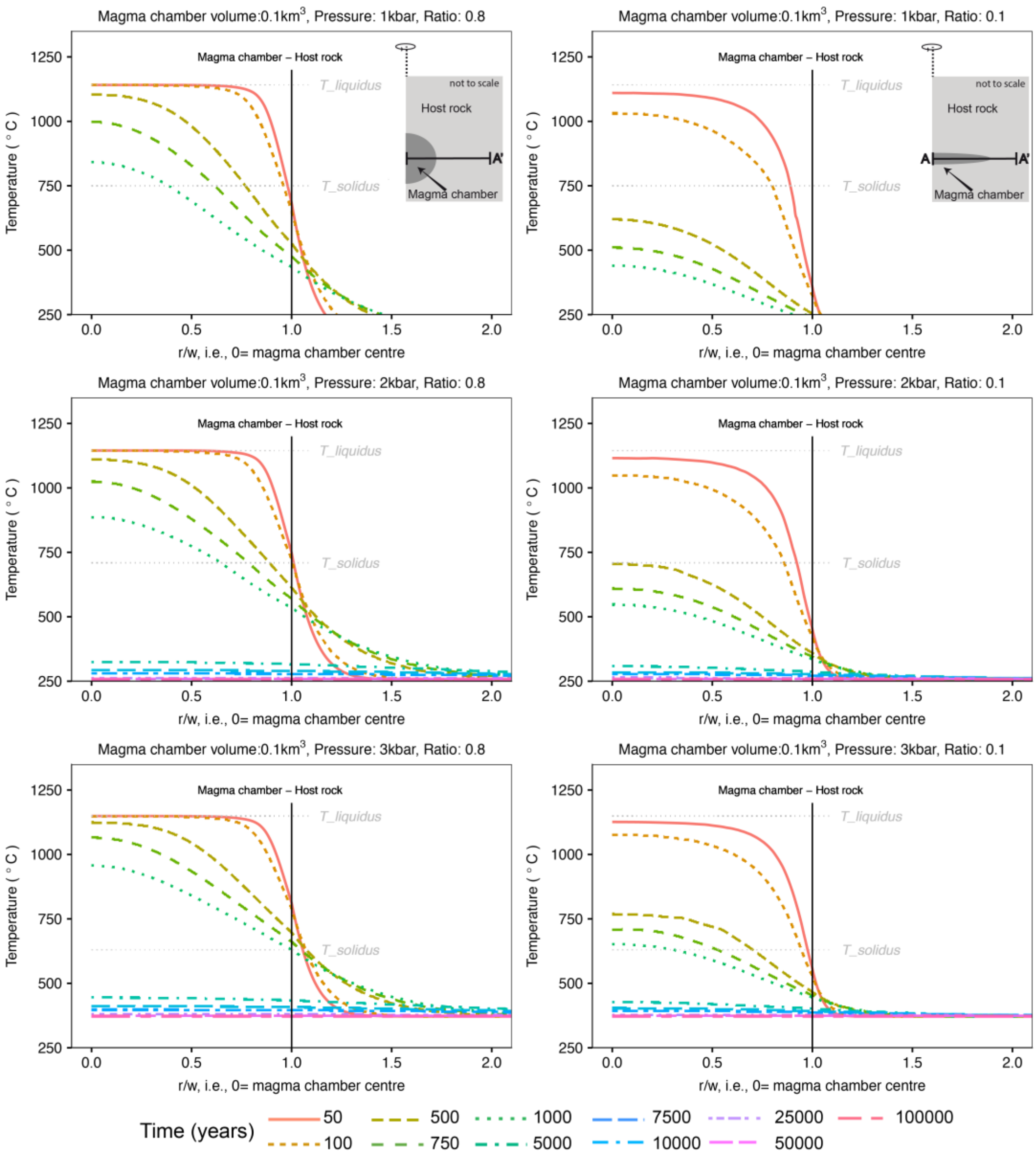

Fig. S9-3: Temperature distribution along a horizontal profile A-A' for those models considering a magma reservoir of V = 0.1 km^3, R= 0.8, or 0.1, and located at different stagnation pressures (P = 1, 2, and 3 kbar). Distance, r along the profile is normalized to the magma chamber width w, i.e., r/w= 1 corresponds to the contact between the magma chamber center and the host rock. Liquidus (T_liquidus) and solidus (T_solidus) temperatures for the magma are also indicated. This figure was generated with RStudio Version 1.0.143 (https://www.rstudio.com/) using ggplot2 package Version 2.1.9000 (http://www.ggplot2.org), a plotting system for R. Final layout of this figure was achieved using Adobe Illustrator CC 2015.3.1 (Copyright © 1987–2016 Adobe Systems Incorporated and its licensors).

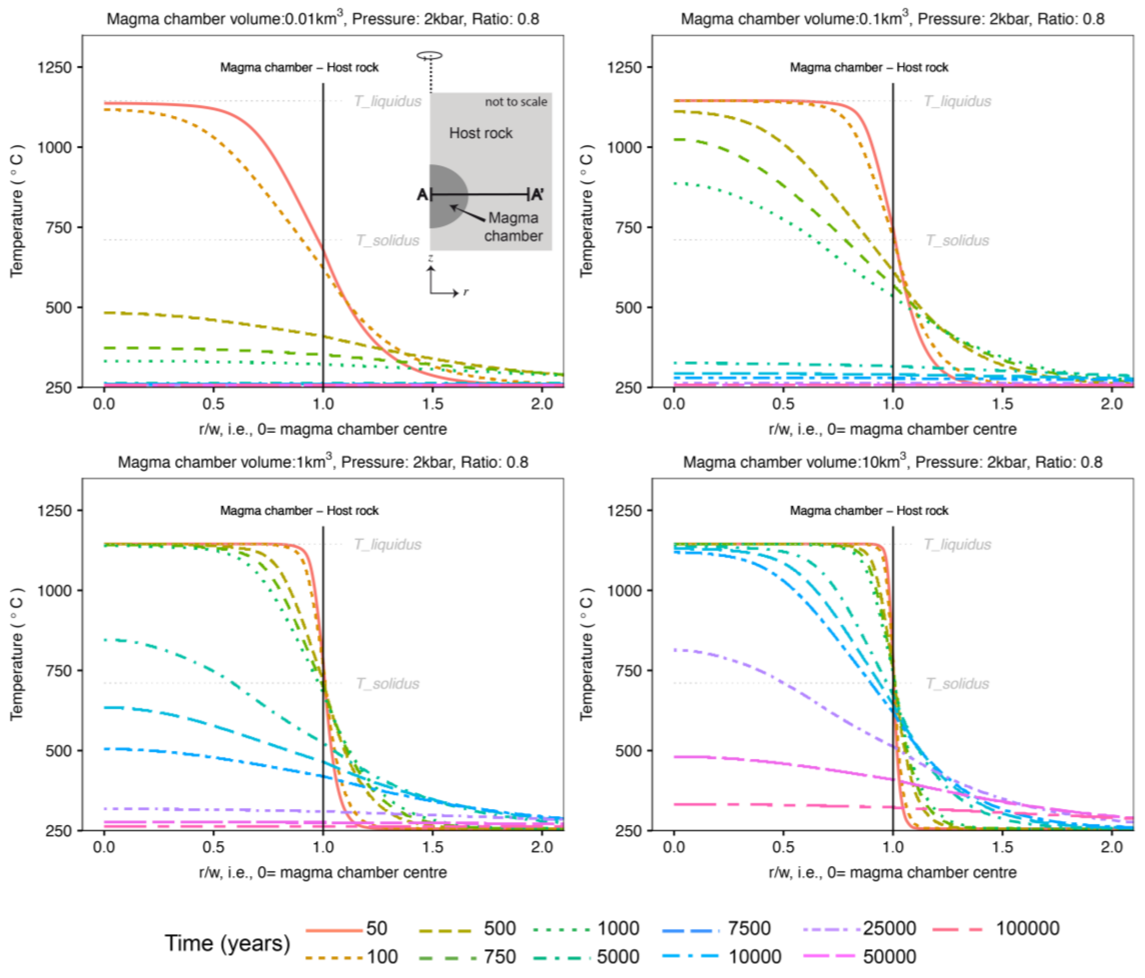

Fig. S9-4: Temperature distribution along a horizontal profile A-A' for those models considering a magma reservoir located at a stagnation pressure of 2 kbar, with R=0.8 and V ranging from 0.01 km^3 to 10 km^3. Distance, r along the profile is normalized to the magma chamber width w, i.e., r/w= 1 corresponds to the contact between the magma chamber center and the host rock. Liquidus (T_liquidus) and solidus (T_solidus) temperatures for the magma are also indicated. This figure was generated with RStudio Version 1.0.143 (https://www.rstudio.com/) using ggplot2 package Version 2.1.9000 (http://www.ggplot2.org), a plotting system for R. Final layout of this figure was achieved using Adobe Illustrator CC 2015.3.1 (Copyright © 1987–2016 Adobe Systems Incorporated and its licensors).

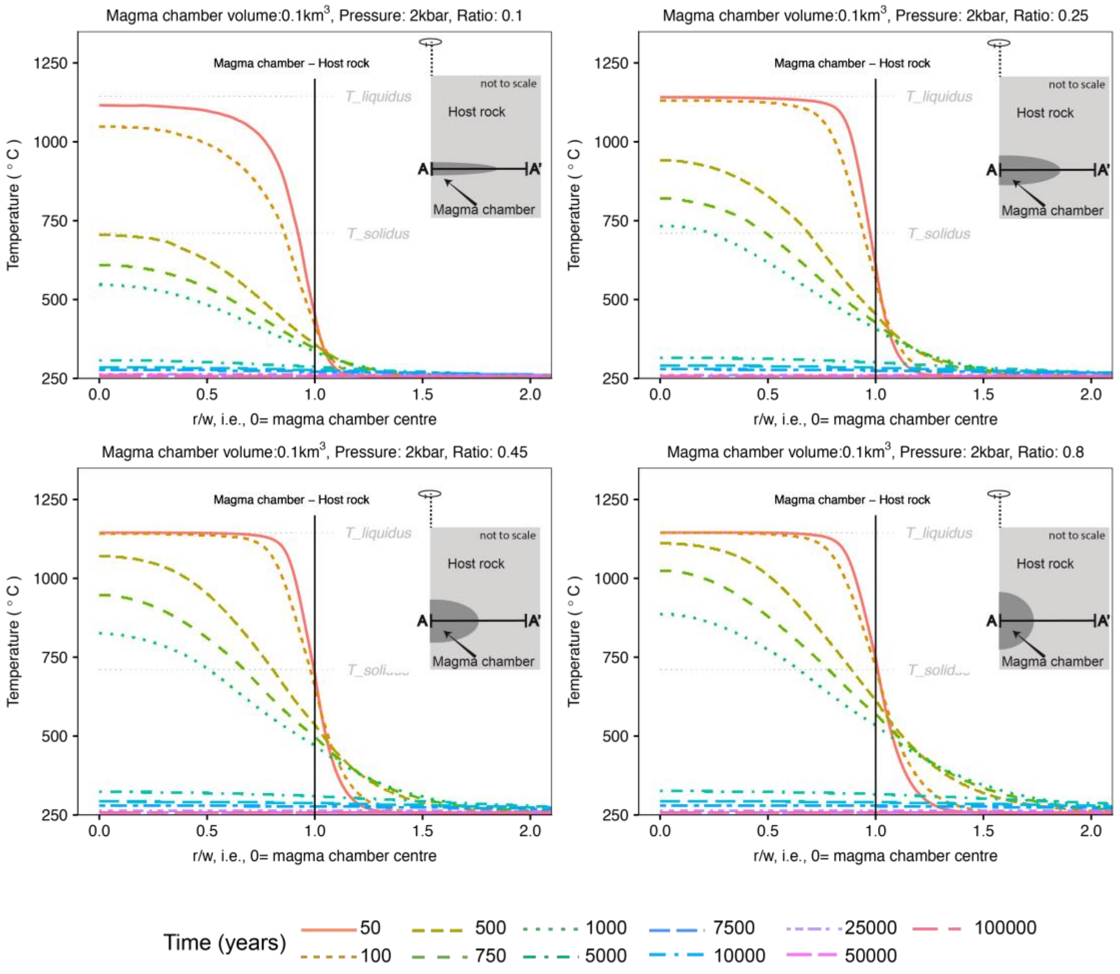

Fig. S9-5: Temperature distribution along a horizontal profile A-A' for those models considering a magma reservoir of V = 0.1 km³ located at a stagnation pressure of 2 kbar, but with different aspect ratios, R. Distance, r along the profile is normalized to the magma chamber width w, i.e., r/w= 1 corresponds to the contact between the magma chamber center and the host rock. Liquidus (T_liquidus) and solidus (T_solidus) temperatures for the magma are also indicated. This figure was generated with RStudio Version 1.0.143 (https://www.rstudio.com/) using ggplot2 package Version 2.1.9000 (http://www.ggplot2.org), a plotting system for R. Final layout of this figure was achieved using Adobe Illustrator CC 2015.3.1 (Copyright © 1987–2016 Adobe Systems Incorporated and its licensors).

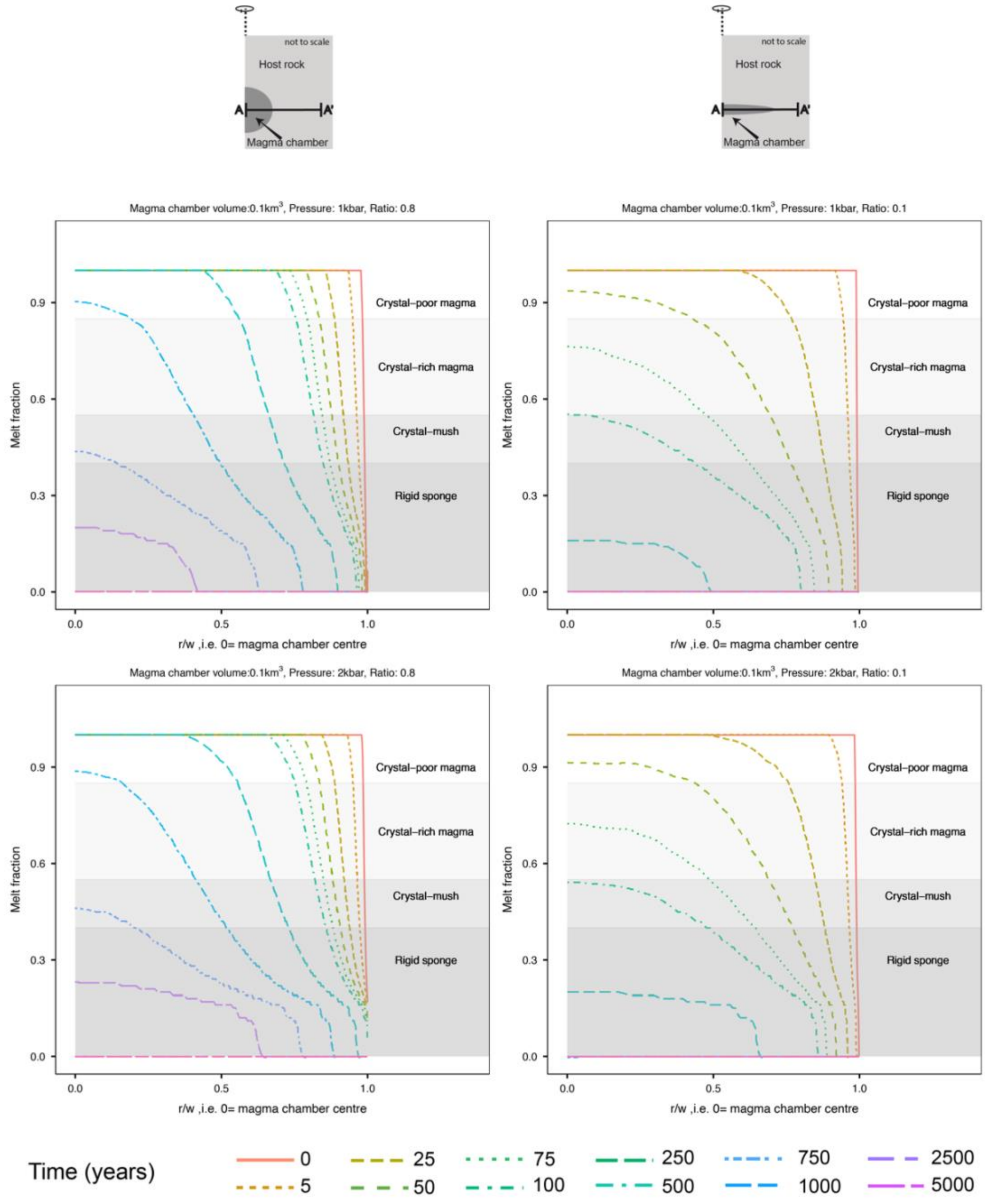

Fig. S9-6: Melt fraction along a horizontal profile A-A' for those models considering a magma reservoir of V = 0.1 km^3, R= 0.8 or 0.1, and located at different stagnation pressures (P= 1 and 2 kbar). Distance, r along the profile is normalized to the magma chamber width w, i.e., r/w= 1 corresponds to the contact between the magma chamber center and the host rock. The definition of the terms crystal-poor and crystal-rich magma, crystal-mush and rigid sponge is based on the crystal content of the magma[14]. This figure was generated with RStudio Version 1.0.143 (https://www.rstudio.com/) using ggplot2 package Version 2.1.9000 (http://www.ggplot2.org), a plotting system for R. Final layout of this figure was achieved using Adobe Illustrator CC 2015.3.1 (Copyright © 1987–2016 Adobe Systems Incorporated and its licensors).

References

1 Rodríguez, C., Geyer, A., Castro, A. & Villaseñor, A. Natural equivalents of thermal gradient experiments. *Journal of Volcanology and Geothermal Research* **298**, 47-58, doi:10.1016/j.jvolgeores.2015.03.021 (2015).

2 Douglas, M. M., Geyer, A., Álvarez-Valero, A. M. & Martí, J. Modeling magmatic accumulations in the upper crust: Metamorphic implications for the country rock. *Journal of Volcanology and Geothermal Research* **319**, 78-92, doi:10.1016/j.jvolgeores.2016.03.008 (2016).

3 Bea, F. Crystallization Dynamics of Granite Magma Chambers in the Absence of Regional Stress: Multiphysics Modeling with Natural Examples. *Journal of Petrology* **51**, 1541-1569, doi:10.1093/petrology/egq028 (2010).

4 Gutiérrez, F. & Parada, M. A. Numerical Modeling of Time-dependent Fluid Dynamics and Differentiation of a Shallow Basaltic Magma Chamber. *Journal of Petrology* **51**, 731-762, doi:10.1093/petrology/egp101 (2010).

5 Canot, E., March, E. & Muhieddine, M. Various Approaches for Solving Problems in Heat Conduction with Phase Change. *International Journal on Finite Volumes* **6**, 1-20 (2009).

6 Hu, H. & Argyropoulos, S. A. Mathematical modelling of solidification and melting: a review. *Modelling and Simulation in Materials Science and Engineering* **4**, 371 (1996).

7 Voller, V. R., Swaminathan, C. R. & Thomas, B. G. Fixed grid techniques for phase change problems: A review. *International Journal for Numerical Methods in Engineering* **30**, 875-898, doi:10.1002/nme.1620300419 (1990).

8 Whittington, A. G., Hofmeister, A. M. & Nabelek, P. I. Temperature-dependent thermal diffusivity of the Earth's crust and implications for magmatism. *Nature* **458**, 319-321, doi:10.1038/nature07818 (2009).

9 Ghiorso, M. S. & Sack, R. O. Chemical mass transfer in magmatic processes IV. A revised and internally consistent thermodynamic model for the interpolation and extrapolation of liquid-solid equilibria in magmatic systems at elevated temperatures and pressures. *Contributions to Mineralogy and Petrology* **119**, 197-212, doi:10.1007/bf00307281 (1995).

10 Asimow, P. D. & Ghiorso, M. S. Algorithmic modifications extending MELTS to calculate subsolidus phase relations. *American Mineralogist* **83**, 1127-1132, doi:10.2138/am-1998-9-1022 (1998).

11 Gualda, G. A. R., Ghiorso, M. S., Lemons, R. V. & Carley, T. L. Rhyolite-MELTS: a Modified Calibration of MELTS Optimized for Silica-rich, Fluid-bearing Magmatic Systems. *Journal of Petrology*, doi:10.1093/petrology/egr080 (2012).

12 Nabelek, P. I., Hofmeister, A. M. & Whittington, A. G. The influence of temperature-dependent thermal diffusivity on the conductive cooling rates of plutons and temperature-time paths in contact aureoles. *Earth and Planetary Science Letters* **317-318**, 157-164, doi:10.1016/j.epsl.2011.11.009 (2012).

13 Turcotte, D. L. & Schubert, G. Geodynamics. second edition edn,456 pp., (Cambridge University Press, Cambridge, 2002).

14 Miller, C. F. Eruptible magma. *Proceedings of the National Academy of Sciences* **113**, 13941-13943, doi:10.1073/pnas.1617105113 (2016).
